# Formation of Ba_3_Nb_0.75_Mn_2.25_O_9_-6H during thermo­chemical reduction of Ba_4_NbMn_3_O_12_-12R

**DOI:** 10.1107/S2056989023003213

**Published:** 2023-04-14

**Authors:** Nicholas A. Strange, Robert T. Bell, James Eujin Park, Kevin H. Stone, Eric N. Coker, David S. Ginley

**Affiliations:** a Stanford Synchrotron Radiation Lightsource, SLAC National Accelerator, Laboratory, Menlo Park, CA 94025, USA; b National Renewable Energy Laboratory, Golden, CO 80401, USA; c Sandia National Laboratories, Albuquerque, NM 87185, USA; Vienna University of Technology, Austria

**Keywords:** crystal structure, powder synchrotron diffraction, complex oxides, hexa­gonal perovskites, solar thermochemical hydrogen production

## Abstract

The resurgence of inter­est in hydrogen-related technologies has stimulated new studies aimed at advancing lesser-developed water-splitting processes, such as solar thermochemical hydrogen production (STCH). Progress in STCH has been largely hindered by a lack of new materials able to efficiently split water at a rate comparable to ceria under identical experimental conditions. BaCe_0.25_Mn_0.75_O_3_ (BCM) recently demonstrated enhanced hydrogen production over ceria and has the potential to further our understanding of two-step thermochemical cycles. A significant feature of the 12R hexa­gonal perovskite structure of BCM is the tendency to, in part, form a 6H polytype at high temperatures and reducing environments (*i.e.*, during the first step of the thermochemical cycle), which may serve to mitigate degradation of the complex oxide. An analogous compound, namely BaNb_0.25_Mn_0.75_O_3_ (BNM) with a 12R structure was synthesized and displays nearly complete conversion to the 6H structure under identical reaction conditions as BCM. The structure of the BNM-6H polytype was determined from Rietveld refinement of synchrotron powder X-ray diffraction data and is presented within the context of the previously established BCM-6H structure.

## Abstract 2

Nearly complete conversion to a Ba_3_Nb_0.75_Mn_2.25_O_9_-6H structure was observed following thermochemical reduction of the parent Ba_4_NbMn_3_O_12_-12R material. The Ba_3_Nb_0.75_Mn_2.25_O_9_-6H structure represents a hexa­gonal perovskite that displays substitution of Mn onto Nb sites in order to satisfy the 3:1 Mn:Nb ratio within the 6H structural motif.

## 1. Chemical context

**Figure 1:**
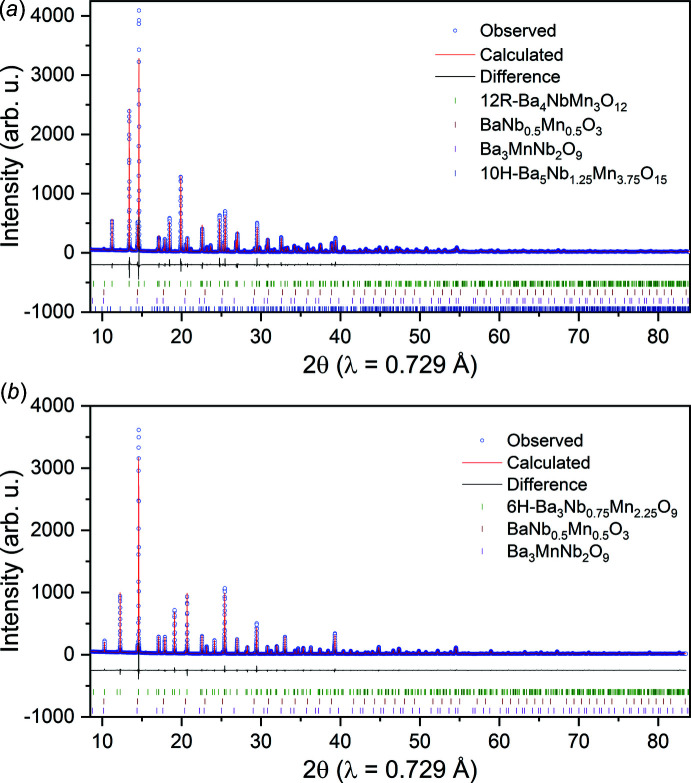
Rietveld refinement plots for the (*a*) pristine BNM (predominantly BNM-12R) and (*b*) reduced BNM samples (predominantly BNM-6H).

Hydrogen production has received considerable attention because of the recent technological advances and initiatives directed towards increasing the yield and reducing the cost of hydrogen production over the next decade. Although electrolysis-based methods of hydrogen production present near-term opportunities for widespread commercialization, incipient methods such as solar thermochemical hydrogen production (STCH) offer higher theoretical efficiencies with the possibility of integration with solar or waste-heat renewable energy sources. Ceria is currently the benchmark material for a direct ‘two-step’ thermochemical cycle. More recently, BaCe_0.25_Mn_0.75_O_3_ (BCM) has demonstrated water-splitting properties commensurate with ceria at lower reaction temperatures (Barcellos *et al.*, 2018 ▸). Known BCM structural phases (BCM-12R, -10H, and -6H polytypes) are related by variations in the [MnO_6_] oligomer size (Fuentes *et al.*, 2004 ▸; Macías *et al.*, 2013 ▸; Strange *et al.*, 2022 ▸). Previous work revealed the presence of a BCM-12R to -6H ([MnO_6_] trimer to dimer) polytypic structural transformation during thermochemical reduction at 1350°C, potentially inhibiting decomposition into simpler refractory oxides at or near water-splitting conditions (Strange *et al.*, 2022 ▸). Compositional analogues to BCM, such as BaNb_0.25_Mn_0.75_O_3_ (BNM) are currently under investigation to determine their water-splitting efficacy and structural behaviors over ranges of environmental conditions. The BNM-12R structure was first reported by Nguyen *et al.* (2019 ▸), where the magnetic behavior of the Mn ions was the primary focus. In the present communication, an alternative pathway was utilized to synthesize the BNM-12R compound with a 92.2 wt% purity as determined by Rietveld refinement of synchrotron powder X-ray diffraction (see Fig. 1 ▸ *a*). The high-purity BNM-12R material was subsequently exposed to low oxygen partial pressure conditions at 1350°C, where the powder exhibited nearly complete conversion to the previously unreported 6H-polytype of BNM (Fig. 1 ▸ *b*), in contrast with BCM-6H, which displayed only partial conversion under identical experimental conditions.

## 2. Structural commentary

**Figure 2:**
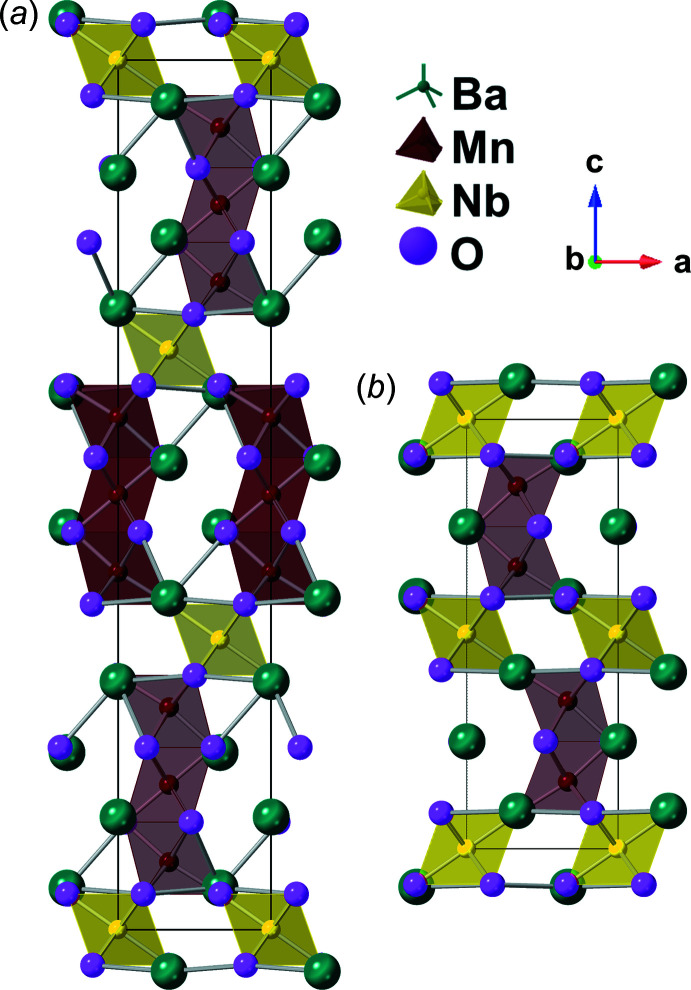
Illustrations of the (*a*) BNM-12R and (*b*) BNM-6H unit cells based on Rietveld structural refinements.

The BNM-12R structure (Fig. 2 ▸ *a*) belongs to a family of hexa­gonal perovskites (Tilley, 2016 ▸) with alternating layers of face-sharing [MnO_6_] trimers separated by ordered corner-sharing [NbO_6_] units and is associated with the Ba_2_NiTeO_6_ structure type (Köhl *et al.*, 1972 ▸). BaO_3_ layers are formed with a cubic–cubic–hexa­gonal–hexa­gonal, (cchh)_3_, stacking. In contrast to the fully stoichiometric BCM-12R structure reported by Fuentes *et al.* (2004 ▸), the analogous fully stoichiometric BNM-12R structure (with a 3:1 ratio of Mn:Nb) requires an average charge of at least +3.667 distributed across the two Mn sites since Nb remains entirely in oxidation state +5 during redox cycles. An average Mn oxidation state of 3.667+ in BNM-12R requires an Mn^4+^:Mn^3+^ cation ratio of 2:1 on the *B* site. This finding suggests that the BNM-12R structure exists with *at minimum* 33% of the Mn cations initially in the 3+ oxidation state. Minor structural impurities consisted primarily of cubic BaNb_0.5_Mn_0.5_O_3_ and (Ba_3_MnNb_2_O_9_)_0.333_ compounds, which contain Mn exclusively in the +3 and +2 oxidation states, respectively. Residual diffraction peaks were indexed to a 10H analogue of BNM (*a* = 5.725, *c* = 23.537 Å), but the observed weight fraction of the species was too low to reliably refine additional parameters of the crystal structure. The three Mn—O bond lengths in BNM-12R are 1.876 (3) Å (6×), 1.935 (3) Å (3×), and 1.968 (3) Å (3×), where the smallest distance corresponds to inter­nal Mn1—O1 bonds within the trimeric unit. For comparison, Mn—O bond length in BaMnO_3_-2H (Cussen & Battle, 2000 ▸), which exhibits Mn^4+^ cations within exclusively face-sharing [MnO_6_] octa­hedra, is 1.904 Å. The Mn—O distances in BNM-12R are also systematically larger than the analogous bond lengths in BCM-12R (1.855, 1.928, and 1.955 Å), which entirely displays Mn^4+^ cations, thereby supporting inherent partial reduction in the as-synthesized BNM-12R compound.

The BNM-6H structure (Fig. 2 ▸ *b*) is also a hexa­gonal perovskite, exhibiting [MnO_6_] dimers, in contrast to the trimers found in BNM-12R, and is of the BaFeO_3-δ_ structure type (Grey *et al.*, 1998 ▸). The dimers in BNM-6H are separated by [NbO_6_] corner-sharing octa­hedra. BaO_3_ units are found with (cch)_2_ stacking. In order to conserve the 3:1 Mn:Nb stoichiometry, partial substitution of Mn onto the Nb sites is necessary. Since the fully stoichiometric BNM-12R structure contains at least 33% Mn^3+^ cations, the formation of BNM-6H must be accompanied in part by the additional oxygen vacancies and further reduction from Mn^4+^ → Mn^3+^. The relative concentrations of structural impurities initially observed in the BNM-12R material were decreased during thermochemical reduction, accounting for only ∼2.4 wt% of the ‘reduced’ material. It is noteworthy that the BNM-10H polytype was no longer detectable following thermochemical reduction. The Mn—O bond lengths within BNM-6H are 1.930 (3) Å (3×), 1.960 (3) Å (3×), and 2.051 (3) Å (3×) and are elongated relative to the BNR-12R [MnO_6_] octa­hedra, establishing further reduction of Mn^4+^ to Mn^3+^. The largest of these values is associated with the (Nb,Mn)—O distance and is dually impacted by Mn and Nb shared occupation and corner sharing [(Nb,Mn)O_6_] octa­hedra. The (Nb,Mn)—O bond length in BNM-6H [2.051 (3) Å] increases only slightly by ∼2.5% relative to the Nb—O distance without occupational disorder [2.001 (3) Å] within BNM-12R during the 12R- to 6H-polytype transformation.

The BNM-12R structure has been investigated for competing ferromagnetic and anti­ferromagnetic inter­actions between neighboring trimers of [MnO_6_] octa­hedra (Nguyen *et al.*, 2019 ▸). The BNM-6H structure discussed herein has substantial substitution of Mn onto Nb sites, potentially leading to a distribution of Mn dimers, penta­mers, and decreasing occurrence of higher *n* chains of 3*n* + 2 [MnO_6_] octa­hedra. The influences of these different length chains of [MnO_6_] octa­hedra on the magnetic properties are a subject of potential inter­est for these materials.

## 3. Database survey

A query was made to the Inorganic Crystal Structural Database (ICSD, version 4.8.0; Zagorac *et al.*, 2019 ▸) to search for related crystal structures within 1% of the reported BNM-6H lattice parameters. Limiting the results to structures with *P*6_3_/*mmc* space-group symmetry, a series of Ba*M*O_3_ (*M* = transition metal) compounds were identified. For many of these structures, the *B*-sites of the *AB*O_3_ compounds are shared between two metals, often with a 2:1 ratio, which satisfies full occupation of atomic sites for *A* and *B* site cations (*i.e*., site mixing is not necessary to achieve the compound stoichiometry). The cation with a larger ionic radius is typically the less abundant species in the 2:1 ratio. The reported BNM-6H structure is one of few structures within this series to display a 3:1 mixing ratio on the *B*-site and is the only structure where mixing is achieved exclusively by the smaller cation (*e.g*., Mn) substituting onto the larger cation (*e.g*., Nb) *B*-site position. In the related BaFe_0.25_Ti_0.75_O_3_ structure, mixing of *B*-site cations is present on both the 2*a* and 4*f* Wyckoff sites. Furthermore, BNM-6H is the only reported structure to display a combination of cations with oxidation states of 5+ (Nb) and 4+/3+ (Mn). The metal–oxygen bond lengths and angles among these Ba*M*O_3_ structures are nominally equivalent (with exception to non-stoichiometric states). The shorter cation bonds with oxygen are slightly less than 2 Å, whereas the longer cation bonds with oxygen are slightly greater than 2 Å. The O—Ba—O and O—*M*—O bond angles reside near their respective ideal values.

## 4. Synthesis and crystallization

*Materials*

Barium nitrate [Ba(NO_3_)_2_, Alfa Aesar ACS, 99+%], niobium(V) oxide (Nb_2_O_5_, Alfa Aesar, 99.9%), manganese(II) nitrate tetra­hydrate [Mn(NO_3_)_2_·4H_2_O, Alfa Aesar, 98%] and anhydrous citric acid (Fisher Scientific, certified ACS) were purchased from their respective vendors and used as received.

*Synthesis of BaNb_0.25_Mn_0.75_O_3_-12R*

For the synthesis of BaNb_0.25_Mn_0.75_O_3_-12R (5 g scale reaction), barium nitrate (5.2319 g), niobium (V) oxide (0.6651 g), manganese (II) nitrate tetra­hydrate (3.7686 g), and anhydrous citric acid (6.3101 g) at a molar ratio of 1:0.25:0.75:1.5 were suspended in ∼25 ml of deionized water. Most of the water was evaporated on a hot plate while stirring until a viscous liquid was obtained. This was dried at 110°C in air overnight, ground into a powder, then self-combusted on a hot plate. The resulting powder was ground, calcined at 800°C (5°C min^−1^) in air for 12 h, then sintered at 1300°C (10°C min^−1^) for another 12 h with inter­mediate grinding. The calcination temperature was determined from the previously reported solid-state synthesis method (Nguyen *et al.*, 2019 ▸).

*Thermogravimetric Analysis*

**Figure 3:**
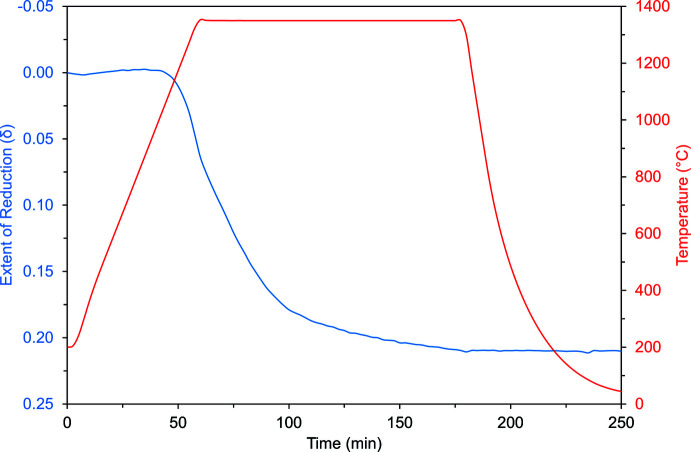
Thermogravimetric analysis data for BNM-12R thermochemically reduced in argon gas at 1350°C.

The thermogravimetric analysis experiment was performed using a Netzsch STA 449 F1 Jupiter thermal analyzer under gas flow rates of 100 ml min^−1^. Baseline correction was performed on an empty crucible with no sample. Argon gas (Matheson, UHP grade) and air (Matheson, ultra-zero grade) were used as received. Before reduction, the as-synthesized Ba_4_NbMn_3_O_12_-12R powder was initially redox-cycled three times. For this, the sample was heated to 1350°C (10°C min^−1^) under Ar, held isothermally for 30 minutes, cooled to 400°C (25°C min^−1^), then held isothermally for 30 minutes (see Fig. 3 ▸). With the gas changed to a mixture of air (80%) and Ar (20%), the sample was heated to 1100°C (10°C min^−1^), held isothermally for 30 minutes, then cooled to 200°C (25°C min^−1^). For repeated cycles, the sample was re-weighed between runs.

The redox-cycled Ba_4_NbMn_3_O_12_-12R was reduced with an oxidation step included in the TGA experiment prior to the reduction to determine the mass reference point at which the sample was fully oxidized. For this, the sample was heated under air to 1100°C (20°C min^−1^), held isothermally for 30 minutes, cooled to 200°C (20°C min^−1^), then held isothermally for 30 minutes. For the reduction, the sample was heated under Ar to 1350°C (20°C min^−1^), held isothermally for 2 h, cooled to 50°C (50°C min^−1^), then held isothermally for 30 minutes. The atmosphere was then changed to air to ensure no mass gain, indicating oxidation, was observed. The thermogram shown is baseline-corrected with the initial oxidation step (for mass reference point) omitted.

## 5. Refinement

Synchrotron powder X-ray diffraction (SPXRD) measurements were performed at the Stanford Synchrotron Radiation Lightsource (SSRL) beam line 2-1 on two BNM materials. The as-synthesized BNM powder, referred to as ‘pristine BNM’, targeted a pure BNM-12R phase. The second sample, referred to as ‘reduced BNM’, was produced by thermochemically reducing the pristine BNM powder as detailed in the thermogravimetric analysis sub-section above. The two BNM samples were prepared in 0.5 mm (0.01 mm wall thickness) glass capillaries. XRD data was acquired using a Pilatus 100K hybrid photon counting detector with portrait orientation. Two-dimensional detector images were normalized to incident beam intensity, stitched, and integrated into one-dimensional diffraction data using a Python script developed at SSRL, specifically for beam line 2-1. Crystal data, data collection and structure refinement details are summarized in Table 1 ▸.

Structural refinements were performed using the Rietveld method in *TOPAS-Academic*, version 7 (Coelho, 2018 ▸). Refinements were performed on the two sets of SPXRD data, with structural parameters associated with their primary structural phases detailed in Table 1 ▸. Thermal displacement parameters and fractional positions were constrained to be equal for atoms occupying the same crystallographic sites. The reduced BNM refinement (*i.e*., BNM-6H) was initially performed by constraining the Mn and Nb occupation of the (0,0,0) site to 1, while allowing the individual occupations to vary. The refinement did not significantly change the composition and subsequent refinements assumed the final 0.75:0.25 Nb:Mn occupations. No restraints were necessary in either structural refinement. The complete weight fractions of primary and secondary structural phases (generated from the Rietveld refinements) are presented for both pristine BNM and reduced BNM in Table 2 ▸. The associated impurity phases are a cubic perovskite-type BaNb_0.5_Mn_0.5_O_3_ (Filipev & Fesenko, 1961 ▸), double perovskite (Ba_3_MnNb_2_O_9_)_0.333_ (Xin *et al.*, 2018 ▸) and a BNM-10H variant within the family of hexa­gonal perovskite polytypes (Macías *et al.*, 2013 ▸).

## Supplementary Material

Crystal structure: contains datablock(s) global, 6H-barium_niobium_manganese_oxide, barium_niobiumV_manganeseIII_oxide, barium_niobiumV_manganeseII_oxide, 12R-barium_niobium_manganese_oxide, barium_niobiumV_manganeseIII_oxide_2, 10H-barium_niobium_manganese_oxide, barium_niobiumV_manganeseII_oxide_2. DOI: 10.1107/S2056989023003213/wm5677sup1.cif

CIF for 6H barium niobium manganese oxide from TOPAS output. DOI: 10.1107/S2056989023003213/wm5677sup2.txt

CIF for 12R barium niobium manganese oxide from TOPAS output. DOI: 10.1107/S2056989023003213/wm5677sup3.txt

CCDC references: 2253240, 2253241, 2253243, 2253244, 2253245, 2253246, 2255313

Additional supporting information:  crystallographic information; 3D view; checkCIF report

## Figures and Tables

**Table 1:** Experimental details

	Ba_4_NbMn_3_O_12_	Ba_6_Nb_1.5_Mn_4.5_O_18_
Crystal data		
*M*_r_	999.02	1498.53
Crystal system, space group	Hexagonal, *R* 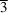 *m*	Hexagonal, *P*6_3_/*m* *m* *c*
Temperature (K)	300	300
*a*, *c* (Å)	5.73256 (3), 28.16620 (19)	5.743123 (13), 14.09930 (5)
*V* (Å^3^)	801.60 (1)	402.74 (1)
*Z*	3	1
Radiation type	Synchrotron X-ray, λ = 0.729277 Å	Synchrotron X-ray, λ = 0.729277 Å
μ (mm^−1^)	20.22	20.12
Specimen shape, size (mm)	Cylinder, 1 × 0.5	Cylinder, 1 × 0.5

Data collection		
Diffractometer	SSRL beam line 2-1	SSRL beam line 2-1
Specimen mounting	Glass capillary	Glass capillary
Data collection mode	Transmission	Transmission
Scan method	Step	Step
2θ values (°)	2θ_min_ = 8.5 2θ_max_ = 84 2θ_step_ = 0.005	2θ_min_ = 5 2θ_max_ = 83.5 2θ_step_ = 0.005

Refinement		
*R* factors and goodness of fit	*R*_p_ = 0.062, *R*_wp_ = 0.087, *R*_exp_ = 0.167, χ^2^ = 0.273	*R*_p_ = 0.043, *R*_wp_ = 0.059, *R*_exp_ = 0.191, χ^2^ = 0.096
No. of parameters	36	30

Computer programs: *spec X-ray Diffraction Software* (Certified Scientific Software, 2017 ▸), *TOPAS-Academic* (Coelho, 2018 ▸), SSRL BL 2-1 Python integration script (local program), *CrystalMaker* (Palmer, 2014 ▸), and *publCIF* (westrip, 2010 ▸).

**Table 2:** Weight fractions of structural phases determined from Rietveld refinements of pristine BNM and reduced BNM XRD data

Structural Phase	Space Group	Pristine BNM	Reduced BNM
Ba_4_NbMn_3_O_12_-12R	*R* 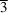 *m*	92.20%	0.00%
Ba_5_Nb_1.25_Mn_3.75_O_15_-10H	*P*6_3_/*mmc*	0.83%	0.00%
Ba_3_Nb_0.75_Mn_2.25_O_9_-6H	*P*6_3_ */mmc*	0.00%	97.69%
BaNb_0.5_Mn_0.5_O_3_	*Pm* 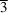 *m*	5.20%	1.95%
(Ba_3_MnNb_2_O_9_)_0.333_	*Fm* 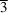 *m*	1.78%	0.35%

## supplementary crystallographic information

### (6H-barium_niobium_manganese_oxide) Crystal data

**Table:**

Ba_6_Nb_1.5_Mn_4.5_O_18_	*Z* = 1
*M**_r_* = 1498.53	*D*_x_ = 6.179 (1) Mg m^−^^3^
Hexagonal, *P*6_3_/*m**m**c*	Synchrotron X-ray radiation, λ = 0.729277 Å
*a* = 5.743123 (13) Å	µ = 20.12 mm^−^^1^
*c* = 14.09930 (5) Å	*T* = 300 K
*V* = 402.74 (1) Å^3^	cylinder, 1 × 0.5 mm

### (6H-barium_niobium_manganese_oxide) Data collection

**Table:**

SSRL beam line 2-1 diffractometer	Scan method: step
Specimen mounting: Glass capillary	2θ_min_ = 5°, 2θ_max_ = 83.5°, 2θ_step_ = 0.005°
Data collection mode: transmission	

### (6H-barium_niobium_manganese_oxide) Refinement

**Table:**

*R*_p_ = 0.043	30 parameters
*R*_wp_ = 0.059	0 restraints
*R*_exp_ = 0.191	

### (6H-barium_niobium_manganese_oxide) Fractional atomic coordinates and isotropic or equivalent isotropic displacement parameters (Å^2^)

**Table:**

	*x*	*y*	*z*	*B*_iso_*/*B*_eq_	Occ. (<1)
Ba1	0	0	0.25	0.93 (3)	
Ba2	0.333333	0.666667	0.08847 (3)	0.93 (3)	
Mn1	0.333333	0.666667	0.65603 (7)	0.44 (3)	
Nb1	0	0	0	0.44 (3)	0.75
Mn2	0	0	0	0.44 (3)	0.25
O1	0.5215 (4)	1.0430 (8)	0.25	1.68 (5)	
O2	0.1690 (3)	0.3380 (6)	0.58332 (19)	1.68 (5)	

### (6H-barium_niobium_manganese_oxide) Geometric parameters (Å, º)

**Table:**

Ba1—O1^i^	2.8795 (4)	Nb1—O2^xxv^	2.051 (3)
Ba1—O1^ii^	2.8795 (4)	Nb1—O2^vii^	2.051 (3)
Ba1—O1^iii^	2.8795 (4)	Mn2—Nb1	0.00000
Ba1—O1^iv^	2.8795 (4)	Mn2—O2^xvii^	2.051 (3)
Ba1—O1^v^	2.8795 (4)	Mn2—O2^xxiv^	2.051 (3)
Ba1—O1^vi^	2.8795 (4)	Mn2—O2^ix^	2.051 (3)
Ba1—O2^vii^	2.889 (3)	Mn2—O2^xi^	2.051 (3)
Ba1—O2^viii^	2.889 (3)	Mn2—O2^xxv^	2.051 (3)
Ba1—O2^ix^	2.889 (3)	Mn2—O2^vii^	2.051 (3)
Ba1—O2^x^	2.889 (3)	O1—Mn1^xvi^	1.960 (3)
Ba1—O2^xi^	2.889 (3)	O1—Mn1^xxii^	1.960 (3)
Ba1—O2^xii^	2.889 (3)	O1—O1^xxvi^	2.501 (7)
Ba2—O2^xiii^	2.8726 (1)	O1—O1^xxvii^	2.501 (7)
Ba2—O2^xiv^	2.8726 (1)	O1—O2^xvi^	2.814 (3)
Ba2—O2^vii^	2.8726 (1)	O1—O2^xxviii^	2.814 (3)
Ba2—O2^xv^	2.8726 (1)	O1—O2^xiv^	2.814 (3)
Ba2—O2^xvi^	2.8726 (1)	O1—O2^xxix^	2.814 (3)
Ba2—O2^ix^	2.8726 (1)	O1—Ba1^xxx^	2.8795 (4)
Ba2—O2^ii^	2.922 (3)	O1—Ba1^xxxi^	2.8795 (4)
Ba2—O2^v^	2.922 (3)	O1—Ba2^v^	2.948 (3)
Ba2—O2^xvii^	2.922 (3)	O1—Ba2	2.948 (3)
Ba2—O1^ii^	2.948 (3)	O2—Mn1	1.930 (3)
Ba2—O1^v^	2.948 (3)	O2—Mn2^xxxii^	2.051 (3)
Ba2—O1	2.948 (3)	O2—Nb1^xxxii^	2.051 (3)
Mn1—O2^xviii^	1.930 (3)	O2—O1^xx^	2.814 (3)
Mn1—O2^xix^	1.930 (3)	O2—O1^xxi^	2.814 (3)
Mn1—O2	1.930 (3)	O2—O2^xviii^	2.832 (5)
Mn1—O1^xx^	1.960 (3)	O2—O2^xix^	2.832 (5)
Mn1—O1^xxi^	1.960 (3)	O2—Ba2^xxxii^	2.8726 (1)
Mn1—O1^xxii^	1.960 (3)	O2—Ba2^xxi^	2.8726 (1)
Mn1—Mn1^xxiii^	2.6498 (18)	O2—O2^xii^	2.889 (5)
Nb1—Mn2	0.00000	O2—O2^x^	2.889 (5)
Nb1—O2^xvii^	2.051 (3)	O2—Ba1^xxxii^	2.889 (3)
Nb1—O2^xxiv^	2.051 (3)	O2—O2^xxxiii^	2.912 (5)
Nb1—O2^ix^	2.051 (3)	O2—O2^xxxiv^	2.912 (5)
Nb1—O2^xi^	2.051 (3)	O2—Ba2^v^	2.922 (3)
			
O1^ii^—Ba1—O1^i^	51.48 (16)	O2^xi^—Mn2—Nb1	
O1^iii^—Ba1—O1^ii^	171.48 (16)	O2^xxv^—Mn2—O2^xi^	89.55 (11)
O1^iii^—Ba1—O1^i^	120.000	O2^xxv^—Mn2—O2^ix^	89.55 (11)
O1^iv^—Ba1—O1^iii^	68.52 (16)	O2^xxv^—Mn2—O2^xxiv^	90.45 (11)
O1^iv^—Ba1—O1^ii^	120.000	O2^xxv^—Mn2—O2^xvii^	90.45 (11)
O1^iv^—Ba1—O1^i^	171.48 (16)	O2^xxv^—Mn2—Nb1	
O1^v^—Ba1—O1^iv^	51.48 (16)	O2^vii^—Mn2—O2^xxv^	180.000
O1^v^—Ba1—O1^iii^	120.000	O2^vii^—Mn2—O2^xi^	90.45 (11)
O1^v^—Ba1—O1^ii^	68.52 (16)	O2^vii^—Mn2—O2^ix^	90.45 (11)
O1^v^—Ba1—O1^i^	120.000	O2^vii^—Mn2—O2^xxiv^	89.55 (11)
O1^vi^—Ba1—O1^v^	171.48 (16)	O2^vii^—Mn2—O2^xvii^	89.55 (11)
O1^vi^—Ba1—O1^iv^	120.000	O2^vii^—Mn2—Nb1	
O1^vi^—Ba1—O1^iii^	51.48 (16)	Mn1^xxii^—O1—Mn1^xvi^	85.08 (18)
O1^vi^—Ba1—O1^ii^	120.000	O1^xxvi^—O1—Mn1^xxii^	50.35 (7)
O1^vi^—Ba1—O1^i^	68.52 (16)	O1^xxvi^—O1—Mn1^xvi^	50.35 (7)
O2^vii^—Ba1—O1^vi^	118.74 (5)	O1^xxvii^—O1—O1^xxvi^	60.000
O2^vii^—Ba1—O1^v^	58.39 (5)	O1^xxvii^—O1—Mn1^xxii^	50.35 (7)
O2^vii^—Ba1—O1^iv^	58.39 (5)	O1^xxvii^—O1—Mn1^xvi^	50.35 (7)
O2^vii^—Ba1—O1^iii^	92.48 (5)	O2^xvi^—O1—O1^xxvii^	63.62 (8)
O2^vii^—Ba1—O1^ii^	92.48 (5)	O2^xvi^—O1—O1^xxvi^	93.37 (9)
O2^vii^—Ba1—O1^i^	118.74 (5)	O2^xvi^—O1—Mn1^xxii^	113.61 (15)
O2^viii^—Ba1—O2^vii^	146.18 (5)	O2^xvi^—O1—Mn1^xvi^	43.23 (6)
O2^viii^—Ba1—O1^vi^	58.39 (5)	O2^xxviii^—O1—O2^xvi^	154.27 (18)
O2^viii^—Ba1—O1^v^	118.74 (5)	O2^xxviii^—O1—O1^xxvii^	93.37 (9)
O2^viii^—Ba1—O1^iv^	92.48 (5)	O2^xxviii^—O1—O1^xxvi^	63.62 (8)
O2^viii^—Ba1—O1^iii^	58.39 (5)	O2^xxviii^—O1—Mn1^xxii^	43.23 (6)
O2^viii^—Ba1—O1^ii^	118.74 (5)	O2^xxviii^—O1—Mn1^xvi^	113.61 (15)
O2^viii^—Ba1—O1^i^	92.48 (5)	O2^xiv^—O1—O2^xxviii^	113.25 (10)
O2^ix^—Ba1—O2^viii^	146.18 (5)	O2^xiv^—O1—O2^xvi^	60.41 (12)
O2^ix^—Ba1—O2^vii^	60.51 (9)	O2^xiv^—O1—O1^xxvii^	93.37 (9)
O2^ix^—Ba1—O1^vi^	92.48 (5)	O2^xiv^—O1—O1^xxvi^	63.62 (8)
O2^ix^—Ba1—O1^v^	92.48 (5)	O2^xiv^—O1—Mn1^xxii^	113.61 (15)
O2^ix^—Ba1—O1^iv^	118.74 (5)	O2^xiv^—O1—Mn1^xvi^	43.23 (6)
O2^ix^—Ba1—O1^iii^	118.74 (5)	O2^xxix^—O1—O2^xiv^	154.27 (18)
O2^ix^—Ba1—O1^ii^	58.39 (5)	O2^xxix^—O1—O2^xxviii^	60.41 (12)
O2^ix^—Ba1—O1^i^	58.39 (5)	O2^xxix^—O1—O2^xvi^	113.25 (10)
O2^x^—Ba1—O2^ix^	108.85 (12)	O2^xxix^—O1—O1^xxvii^	63.62 (8)
O2^x^—Ba1—O2^viii^	60.51 (9)	O2^xxix^—O1—O1^xxvi^	93.37 (9)
O2^x^—Ba1—O2^vii^	146.18 (5)	O2^xxix^—O1—Mn1^xxii^	43.23 (6)
O2^x^—Ba1—O1^vi^	92.48 (5)	O2^xxix^—O1—Mn1^xvi^	113.61 (15)
O2^x^—Ba1—O1^v^	92.48 (5)	Ba1^xxx^—O1—O2^xxix^	60.98 (6)
O2^x^—Ba1—O1^iv^	118.74 (5)	Ba1^xxx^—O1—O2^xiv^	121.21 (6)
O2^x^—Ba1—O1^iii^	118.74 (5)	Ba1^xxx^—O1—O2^xxviii^	121.21 (6)
O2^x^—Ba1—O1^ii^	58.39 (5)	Ba1^xxx^—O1—O2^xvi^	60.98 (6)
O2^x^—Ba1—O1^i^	58.39 (5)	Ba1^xxx^—O1—O1^xxvii^	64.26 (8)
O2^xi^—Ba1—O2^x^	146.18 (5)	Ba1^xxx^—O1—O1^xxvi^	124.26 (8)
O2^xi^—Ba1—O2^ix^	60.51 (9)	Ba1^xxx^—O1—Mn1^xxii^	93.14 (5)
O2^xi^—Ba1—O2^viii^	108.85 (12)	Ba1^xxx^—O1—Mn1^xvi^	93.14 (5)
O2^xi^—Ba1—O2^vii^	60.51 (9)	Ba1^xxxi^—O1—Ba1^xxx^	171.48 (16)
O2^xi^—Ba1—O1^vi^	58.39 (5)	Ba1^xxxi^—O1—O2^xxix^	121.21 (6)
O2^xi^—Ba1—O1^v^	118.74 (5)	Ba1^xxxi^—O1—O2^xiv^	60.98 (6)
O2^xi^—Ba1—O1^iv^	92.48 (5)	Ba1^xxxi^—O1—O2^xxviii^	60.98 (6)
O2^xi^—Ba1—O1^iii^	58.39 (5)	Ba1^xxxi^—O1—O2^xvi^	121.21 (6)
O2^xi^—Ba1—O1^ii^	118.74 (5)	Ba1^xxxi^—O1—O1^xxvii^	124.26 (8)
O2^xi^—Ba1—O1^i^	92.48 (5)	Ba1^xxxi^—O1—O1^xxvi^	64.26 (8)
O2^xii^—Ba1—O2^xi^	146.18 (5)	Ba1^xxxi^—O1—Mn1^xxii^	93.14 (5)
O2^xii^—Ba1—O2^x^	60.51 (9)	Ba1^xxxi^—O1—Mn1^xvi^	93.14 (5)
O2^xii^—Ba1—O2^ix^	146.18 (5)	Ba2^v^—O1—Ba1^xxxi^	87.30 (6)
O2^xii^—Ba1—O2^viii^	60.51 (9)	Ba2^v^—O1—Ba1^xxx^	87.30 (6)
O2^xii^—Ba1—O2^vii^	108.85 (12)	Ba2^v^—O1—O2^xxix^	59.75 (4)
O2^xii^—Ba1—O1^vi^	118.74 (5)	Ba2^v^—O1—O2^xiv^	141.86 (12)
O2^xii^—Ba1—O1^v^	58.39 (5)	Ba2^v^—O1—O2^xxviii^	59.75 (4)
O2^xii^—Ba1—O1^iv^	58.39 (5)	Ba2^v^—O1—O2^xvi^	141.86 (12)
O2^xii^—Ba1—O1^iii^	92.48 (5)	Ba2^v^—O1—O1^xxvii^	123.36 (5)
O2^xii^—Ba1—O1^ii^	92.48 (5)	Ba2^v^—O1—O1^xxvi^	123.36 (5)
O2^xii^—Ba1—O1^i^	118.74 (5)	Ba2^v^—O1—Mn1^xxii^	86.88 (3)
O2^xiv^—Ba2—O2^xiii^	119.937 (2)	Ba2^v^—O1—Mn1^xvi^	171.96 (15)
O2^vii^—Ba2—O2^xiv^	176.96 (11)	Ba2—O1—Ba2^v^	101.17 (12)
O2^vii^—Ba2—O2^xiii^	59.06 (13)	Ba2—O1—Ba1^xxxi^	87.30 (6)
O2^xv^—Ba2—O2^vii^	119.937 (2)	Ba2—O1—Ba1^xxx^	87.30 (6)
O2^xv^—Ba2—O2^xiv^	60.90 (13)	Ba2—O1—O2^xxix^	141.86 (12)
O2^xv^—Ba2—O2^xiii^	176.96 (11)	Ba2—O1—O2^xiv^	59.75 (4)
O2^xvi^—Ba2—O2^xv^	119.937 (2)	Ba2—O1—O2^xxviii^	141.86 (12)
O2^xvi^—Ba2—O2^vii^	119.937 (2)	Ba2—O1—O2^xvi^	59.75 (4)
O2^xvi^—Ba2—O2^xiv^	59.06 (13)	Ba2—O1—O1^xxvii^	123.36 (5)
O2^xvi^—Ba2—O2^xiii^	60.90 (13)	Ba2—O1—O1^xxvi^	123.36 (5)
O2^ix^—Ba2—O2^xvi^	176.96 (11)	Ba2—O1—Mn1^xxii^	171.96 (15)
O2^ix^—Ba2—O2^xv^	59.06 (13)	Ba2—O1—Mn1^xvi^	86.88 (3)
O2^ix^—Ba2—O2^vii^	60.90 (13)	Mn2^xxxii^—O2—Mn1	177.14 (13)
O2^ix^—Ba2—O2^xiv^	119.937 (2)	Nb1^xxxii^—O2—Mn2^xxxii^	0.000
O2^ix^—Ba2—O2^xiii^	119.937 (2)	Nb1^xxxii^—O2—Mn1	177.14 (13)
O2^ii^—Ba2—O2^ix^	117.75 (4)	O1^xx^—O2—Nb1^xxxii^	138.09 (13)
O2^ii^—Ba2—O2^xvi^	59.80 (10)	O1^xx^—O2—Mn2^xxxii^	138.09 (13)
O2^ii^—Ba2—O2^xv^	117.75 (4)	O1^xx^—O2—Mn1	44.07 (9)
O2^ii^—Ba2—O2^vii^	88.54 (6)	O1^xxi^—O2—O1^xx^	52.76 (15)
O2^ii^—Ba2—O2^xiv^	88.54 (6)	O1^xxi^—O2—Nb1^xxxii^	138.09 (13)
O2^ii^—Ba2—O2^xiii^	59.80 (10)	O1^xxi^—O2—Mn2^xxxii^	138.09 (13)
O2^v^—Ba2—O2^ii^	57.96 (10)	O1^xxi^—O2—Mn1	44.07 (9)
O2^v^—Ba2—O2^ix^	88.54 (6)	O2^xviii^—O2—O1^xxi^	86.63 (9)
O2^v^—Ba2—O2^xvi^	88.54 (6)	O2^xviii^—O2—O1^xx^	59.80 (6)
O2^v^—Ba2—O2^xv^	59.80 (10)	O2^xviii^—O2—Nb1^xxxii^	135.22 (5)
O2^v^—Ba2—O2^vii^	117.75 (4)	O2^xviii^—O2—Mn2^xxxii^	135.22 (5)
O2^v^—Ba2—O2^xiv^	59.80 (10)	O2^xviii^—O2—Mn1	42.80 (6)
O2^v^—Ba2—O2^xiii^	117.75 (4)	O2^xix^—O2—O2^xviii^	60.000
O2^xvii^—Ba2—O2^v^	57.96 (10)	O2^xix^—O2—O1^xxi^	59.80 (6)
O2^xvii^—Ba2—O2^ii^	57.96 (10)	O2^xix^—O2—O1^xx^	86.63 (9)
O2^xvii^—Ba2—O2^ix^	59.80 (10)	O2^xix^—O2—Nb1^xxxii^	135.22 (5)
O2^xvii^—Ba2—O2^xvi^	117.75 (4)	O2^xix^—O2—Mn2^xxxii^	135.22 (5)
O2^xvii^—Ba2—O2^xv^	88.54 (6)	O2^xix^—O2—Mn1	42.80 (6)
O2^xvii^—Ba2—O2^vii^	59.80 (10)	Ba2^xxxii^—O2—O2^xix^	120.45 (6)
O2^xvii^—Ba2—O2^xiv^	117.75 (4)	Ba2^xxxii^—O2—O2^xviii^	60.47 (6)
O2^xvii^—Ba2—O2^xiii^	88.54 (6)	Ba2^xxxii^—O2—O1^xxi^	115.19 (11)
O1^ii^—Ba2—O2^xvii^	117.56 (7)	Ba2^xxxii^—O2—O1^xx^	62.44 (8)
O1^ii^—Ba2—O2^v^	117.56 (7)	Ba2^xxxii^—O2—Nb1^xxxii^	90.45 (6)
O1^ii^—Ba2—O2^ii^	174.60 (8)	Ba2^xxxii^—O2—Mn2^xxxii^	90.45 (6)
O1^ii^—Ba2—O2^ix^	57.81 (7)	Ba2^xxxii^—O2—Mn1	89.62 (6)
O1^ii^—Ba2—O2^xvi^	124.52 (7)	Ba2^xxi^—O2—Ba2^xxxii^	176.96 (11)
O1^ii^—Ba2—O2^xv^	57.81 (7)	Ba2^xxi^—O2—O2^xix^	60.47 (6)
O1^ii^—Ba2—O2^vii^	91.41 (6)	Ba2^xxi^—O2—O2^xviii^	120.45 (6)
O1^ii^—Ba2—O2^xiv^	91.41 (6)	Ba2^xxi^—O2—O1^xxi^	62.44 (8)
O1^ii^—Ba2—O2^xiii^	124.52 (7)	Ba2^xxi^—O2—O1^xx^	115.19 (11)
O1^v^—Ba2—O1^ii^	66.72 (10)	Ba2^xxi^—O2—Nb1^xxxii^	90.45 (6)
O1^v^—Ba2—O2^xvii^	117.56 (7)	Ba2^xxi^—O2—Mn2^xxxii^	90.45 (6)
O1^v^—Ba2—O2^v^	174.60 (8)	Ba2^xxi^—O2—Mn1	89.62 (6)
O1^v^—Ba2—O2^ii^	117.56 (7)	O2^xii^—O2—Ba2^xxi^	60.95 (6)
O1^v^—Ba2—O2^ix^	91.41 (6)	O2^xii^—O2—Ba2^xxxii^	121.46 (10)
O1^v^—Ba2—O2^xvi^	91.41 (6)	O2^xii^—O2—O2^xix^	90.000
O1^v^—Ba2—O2^xv^	124.52 (7)	O2^xii^—O2—O2^xviii^	120.26 (6)
O1^v^—Ba2—O2^vii^	57.81 (7)	O2^xii^—O2—O1^xxi^	123.34 (9)
O1^v^—Ba2—O2^xiv^	124.52 (7)	O2^xii^—O2—O1^xx^	175.89 (9)
O1^v^—Ba2—O2^xiii^	57.81 (7)	O2^xii^—O2—Nb1^xxxii^	45.22 (5)
O1—Ba2—O1^v^	66.72 (10)	O2^xii^—O2—Mn2^xxxii^	45.22 (5)
O1—Ba2—O1^ii^	66.72 (10)	O2^xii^—O2—Mn1	132.73 (7)
O1—Ba2—O2^xvii^	174.60 (8)	O2^x^—O2—O2^xii^	60.52 (12)
O1—Ba2—O2^v^	117.56 (7)	O2^x^—O2—Ba2^xxi^	121.46 (10)
O1—Ba2—O2^ii^	117.56 (7)	O2^x^—O2—Ba2^xxxii^	60.95 (6)
O1—Ba2—O2^ix^	124.52 (7)	O2^x^—O2—O2^xix^	120.26 (6)
O1—Ba2—O2^xvi^	57.81 (7)	O2^x^—O2—O2^xviii^	90.000
O1—Ba2—O2^xv^	91.41 (6)	O2^x^—O2—O1^xxi^	175.89 (9)
O1—Ba2—O2^vii^	124.52 (7)	O2^x^—O2—O1^xx^	123.34 (9)
O1—Ba2—O2^xiv^	57.81 (7)	O2^x^—O2—Nb1^xxxii^	45.22 (5)
O1—Ba2—O2^xiii^	91.41 (6)	O2^x^—O2—Mn2^xxxii^	45.22 (5)
O2^xix^—Mn1—O2^xviii^	94.39 (12)	O2^x^—O2—Mn1	132.73 (7)
O2—Mn1—O2^xix^	94.39 (12)	Ba1^xxxii^—O2—O2^x^	119.49 (8)
O2—Mn1—O2^xviii^	94.39 (12)	Ba1^xxxii^—O2—O2^xii^	119.49 (8)
O1^xx^—Mn1—O2	92.70 (9)	Ba1^xxxii^—O2—Ba2^xxi^	88.55 (6)
O1^xx^—Mn1—O2^xix^	169.55 (13)	Ba1^xxxii^—O2—Ba2^xxxii^	88.55 (6)
O1^xx^—Mn1—O2^xviii^	92.70 (9)	Ba1^xxxii^—O2—O2^xix^	120.25 (5)
O1^xxi^—Mn1—O1^xx^	79.30 (14)	Ba1^xxxii^—O2—O2^xviii^	120.25 (5)
O1^xxi^—Mn1—O2	92.70 (9)	Ba1^xxxii^—O2—O1^xxi^	60.63 (6)
O1^xxi^—Mn1—O2^xix^	92.70 (9)	Ba1^xxxii^—O2—O1^xx^	60.63 (6)
O1^xxi^—Mn1—O2^xviii^	169.55 (13)	Ba1^xxxii^—O2—Nb1^xxxii^	89.37 (10)
O1^xxii^—Mn1—O1^xxi^	79.30 (14)	Ba1^xxxii^—O2—Mn2^xxxii^	89.37 (10)
O1^xxii^—Mn1—O1^xx^	79.30 (14)	Ba1^xxxii^—O2—Mn1	93.48 (10)
O1^xxii^—Mn1—O2	169.55 (13)	O2^xxxiii^—O2—Ba1^xxxii^	59.75 (5)
O1^xxii^—Mn1—O2^xix^	92.70 (9)	O2^xxxiii^—O2—O2^x^	90.000
O1^xxii^—Mn1—O2^xviii^	92.70 (9)	O2^xxxiii^—O2—O2^xii^	59.74 (6)
Mn1^xxiii^—Mn1—O1^xxii^	47.46 (9)	O2^xxxiii^—O2—Ba2^xxi^	59.55 (6)
Mn1^xxiii^—Mn1—O1^xxi^	47.46 (9)	O2^xxxiii^—O2—Ba2^xxxii^	119.53 (6)
Mn1^xxiii^—Mn1—O1^xx^	47.46 (9)	O2^xxxiii^—O2—O2^xix^	120.000
Mn1^xxiii^—Mn1—O2	122.09 (9)	O2^xxxiii^—O2—O2^xviii^	180.0000 (1)
Mn1^xxiii^—Mn1—O2^xix^	122.09 (9)	O2^xxxiii^—O2—O1^xxi^	93.37 (9)
Mn1^xxiii^—Mn1—O2^xviii^	122.09 (9)	O2^xxxiii^—O2—O1^xx^	120.20 (6)
O2^xvii^—Nb1—Mn2		O2^xxxiii^—O2—Nb1^xxxii^	44.78 (5)
O2^xxiv^—Nb1—O2^xvii^	90.45 (11)	O2^xxxiii^—O2—Mn2^xxxii^	44.78 (5)
O2^xxiv^—Nb1—Mn2		O2^xxxiii^—O2—Mn1	137.20 (6)
O2^ix^—Nb1—O2^xxiv^	180.0000 (1)	O2^xxxiv^—O2—O2^xxxiii^	60.000
O2^ix^—Nb1—O2^xvii^	89.55 (11)	O2^xxxiv^—O2—Ba1^xxxii^	59.75 (5)
O2^ix^—Nb1—Mn2		O2^xxxiv^—O2—O2^x^	59.74 (6)
O2^xi^—Nb1—O2^ix^	90.45 (11)	O2^xxxiv^—O2—O2^xii^	90.000
O2^xi^—Nb1—O2^xxiv^	89.55 (11)	O2^xxxiv^—O2—Ba2^xxi^	119.53 (6)
O2^xi^—Nb1—O2^xvii^	180.0000 (1)	O2^xxxiv^—O2—Ba2^xxxii^	59.55 (6)
O2^xi^—Nb1—Mn2		O2^xxxiv^—O2—O2^xix^	180.0000 (1)
O2^xxv^—Nb1—O2^xi^	89.55 (11)	O2^xxxiv^—O2—O2^xviii^	120.000
O2^xxv^—Nb1—O2^ix^	89.55 (11)	O2^xxxiv^—O2—O1^xxi^	120.20 (6)
O2^xxv^—Nb1—O2^xxiv^	90.45 (11)	O2^xxxiv^—O2—O1^xx^	93.37 (9)
O2^xxv^—Nb1—O2^xvii^	90.45 (11)	O2^xxxiv^—O2—Nb1^xxxii^	44.78 (5)
O2^xxv^—Nb1—Mn2		O2^xxxiv^—O2—Mn2^xxxii^	44.78 (5)
O2^vii^—Nb1—O2^xxv^	180.000	O2^xxxiv^—O2—Mn1	137.20 (6)
O2^vii^—Nb1—O2^xi^	90.45 (11)	Ba2^v^—O2—O2^xxxiv^	118.98 (5)
O2^vii^—Nb1—O2^ix^	90.45 (11)	Ba2^v^—O2—O2^xxxiii^	118.98 (5)
O2^vii^—Nb1—O2^xxiv^	89.55 (11)	Ba2^v^—O2—Ba1^xxxii^	178.44 (12)
O2^vii^—Nb1—O2^xvii^	89.55 (11)	Ba2^v^—O2—O2^x^	59.25 (7)
O2^vii^—Nb1—Mn2		Ba2^v^—O2—O2^xii^	59.25 (7)
O2^xvii^—Mn2—Nb1		Ba2^v^—O2—Ba2^xxi^	91.46 (6)
O2^xxiv^—Mn2—O2^xvii^	90.45 (11)	Ba2^v^—O2—Ba2^xxxii^	91.46 (6)
O2^xxiv^—Mn2—Nb1		Ba2^v^—O2—O2^xix^	61.02 (5)
O2^ix^—Mn2—O2^xxiv^	180.0000 (1)	Ba2^v^—O2—O2^xviii^	61.02 (5)
O2^ix^—Mn2—O2^xvii^	89.55 (11)	Ba2^v^—O2—O1^xxi^	120.71 (10)
O2^ix^—Mn2—Nb1		Ba2^v^—O2—O1^xx^	120.71 (10)
O2^xi^—Mn2—O2^ix^	90.45 (11)	Ba2^v^—O2—Nb1^xxxii^	89.07 (9)
O2^xi^—Mn2—O2^xxiv^	89.55 (11)	Ba2^v^—O2—Mn2^xxxii^	89.07 (9)
O2^xi^—Mn2—O2^xvii^	180.0000 (1)	Ba2^v^—O2—Mn1	88.07 (12)

Symmetry codes: (i) *x*−1, *y*−1, *z*; (ii) −*y*+1, −*x*+1, −*z*+1/2; (iii) −*y*+1, −*x*, −*z*+1/2; (iv) *x*, *y*−1, *z*; (v) −*x*+*y*, −*x*+1, −*z*+1/2; (vi) −*x*+*y*−1, −*x*, −*z*+1/2; (vii) *y*, −*x*+*y*, *z*−1/2; (viii) −*x*, −*y*, −*z*+1; (ix) −*x*, −*x*+*y*, *z*−1/2; (x) *x*−*y*, *x*, −*z*+1; (xi) −*x*, −*y*, *z*−1/2; (xii) *y*, −*x*+*y*, −*z*+1; (xiii) −*x*+1, −*y*+1, *z*−1/2; (xiv) *y*, −*x*+*y*+1, *z*−1/2; (xv) −*x*, −*y*+1, *z*−1/2; (xvi) −*x*+1, −*x*+*y*+1, *z*−1/2; (xvii) −*x*+*y*, *y*, −*z*+1/2; (xviii) −*x*+*y*, −*x*+1, *z*; (xix) −*y*+1, −*x*+1, *z*; (xx) *y*−1, −*x*+*y*, −*z*+1; (xxi) −*x*+1, −*x*+*y*, *z*+1/2; (xxii) −*x*+1, −*y*+2, −*z*+1; (xxiii) −*x*+*y*, −*x*+1, −*z*+3/2; (xxiv) −*x*+*y*, −*x*, −*z*+1/2; (xxv) −*y*, −*x*, −*z*+1/2; (xxvi) −*x*+*y*, −*x*+2, −*z*+1/2; (xxvii) −*y*+2, −*x*+2, −*z*+1/2; (xxviii) *y*, −*x*+*y*+1, −*z*+1; (xxix) *x*−*y*+1, *x*+1, −*z*+1; (xxx) *x*+1, *y*+1, *z*; (xxxi) *x*, *y*+1, *z*; (xxxii) −*x*, −*x*+*y*, *z*+1/2; (xxxiii) −*x*+*y*, −*x*, *z*; (xxxiv) −*y*, −*x*, *z*.

### (barium_niobiumV_manganeseIII_oxide) Crystal data

**Table:**

BaNb0.5Mn0.5O_3_	*V* = 69.01 (1) Å^3^
*M**_r_* = 259.25	*Z* = 1
Cubic, *P**m*3*m*	*D*_x_ = 6.238 (1) Mg m^−^^3^
*a* = 4.10182 (5) Å	*T* = 300 K

### (barium_niobiumV_manganeseIII_oxide) Data collection

**Table:**

SSRL beam line 2-1 diffractometer	

### (barium_niobiumV_manganeseIII_oxide) Fractional atomic coordinates and isotropic or equivalent isotropic displacement parameters (Å^2^)

**Table:**

	*x*	*y*	*z*	*B*_iso_*/*B*_eq_	Occ. (<1)
Ba1	0	0	0	0.1	
Mn1	0.5	0.5	0.5	0.1	0.5
Nb1	0.5	0.5	0.5	0.1	0.5
O1	0.5	0.5	0	0.1	

### (barium_niobiumV_manganeseIII_oxide) Geometric parameters (Å, º)

**Table:**

Ba1—O1^i^	2.9004 (1)	Nb1—O1^xiii^	2.0509 (1)
Ba1—O1^ii^	2.9004 (1)	Nb1—O1^viii^	2.0509 (1)
Ba1—O1^iii^	2.9004 (1)	Nb1—O1	2.0509 (1)
Ba1—O1^iv^	2.9004 (1)	Nb1—O1^xiv^	2.0509 (1)
Ba1—O1^v^	2.9004 (1)	Nb1—O1^vii^	2.0509 (1)
Ba1—O1^vi^	2.9004 (1)	O1—Mn1	2.0509 (1)
Ba1—O1^vii^	2.9004 (1)	O1—Mn1^xv^	2.0509 (1)
Ba1—O1^viii^	2.9004 (1)	O1—Nb1	2.0509 (1)
Ba1—O1	2.9004 (1)	O1—Nb1^xv^	2.0509 (1)
Ba1—O1^ix^	2.9004 (1)	O1—Ba1^xvi^	2.9004 (1)
Ba1—O1^x^	2.9004 (1)	O1—Ba1^xvii^	2.9004 (1)
Ba1—O1^xi^	2.9004 (1)	O1—O1^xviii^	2.9004 (1)
Mn1—Nb1	0.00000	O1—O1^xix^	2.9004 (1)
Mn1—O1^xii^	2.0509 (1)	O1—O1^viii^	2.9004 (1)
Mn1—O1^xiii^	2.0509 (1)	O1—O1^vii^	2.9004 (1)
Mn1—O1^viii^	2.0509 (1)	O1—Ba1^xx^	2.9004 (1)
Mn1—O1	2.0509 (1)	O1—O1^xii^	2.9004 (1)
Mn1—O1^xiv^	2.0509 (1)	O1—O1^xiii^	2.9004 (1)
Mn1—O1^vii^	2.0509 (1)	O1—O1^ii^	2.9004 (1)
Nb1—Mn1	0.00000	O1—O1^i^	2.9004 (1)
Nb1—O1^xii^	2.0509 (1)	O1—Ba1	2.9004 (1)
			
O1^ii^—Ba1—O1^i^	60.000	Ba1^xvi^—O1—Nb1^xv^	90.000
O1^iii^—Ba1—O1^ii^	60.000	Ba1^xvi^—O1—Nb1	90.000
O1^iii^—Ba1—O1^i^	120.000	Ba1^xvi^—O1—Mn1^xv^	90.000
O1^iv^—Ba1—O1^iii^	180.000	Ba1^xvi^—O1—Mn1	90.000
O1^iv^—Ba1—O1^ii^	120.000	Ba1^xvii^—O1—Ba1^xvi^	180.000
O1^iv^—Ba1—O1^i^	60.000	Ba1^xvii^—O1—Nb1^xv^	90.000
O1^v^—Ba1—O1^iv^	120.000	Ba1^xvii^—O1—Nb1	90.000
O1^v^—Ba1—O1^iii^	60.000	Ba1^xvii^—O1—Mn1^xv^	90.000
O1^v^—Ba1—O1^ii^	120.000	Ba1^xvii^—O1—Mn1	90.000
O1^v^—Ba1—O1^i^	180.000	O1^xviii^—O1—Ba1^xvii^	60.000
O1^vi^—Ba1—O1^v^	60.000	O1^xviii^—O1—Ba1^xvi^	120.000
O1^vi^—Ba1—O1^iv^	60.000	O1^xviii^—O1—Nb1^xv^	45.000
O1^vi^—Ba1—O1^iii^	120.000	O1^xviii^—O1—Nb1	135.000
O1^vi^—Ba1—O1^ii^	180.000	O1^xviii^—O1—Mn1^xv^	45.000
O1^vi^—Ba1—O1^i^	120.000	O1^xviii^—O1—Mn1	135.000
O1^vii^—Ba1—O1^vi^	60.000	O1^xix^—O1—O1^xviii^	60.000
O1^vii^—Ba1—O1^v^	90.000	O1^xix^—O1—Ba1^xvii^	120.000
O1^vii^—Ba1—O1^iv^	60.000	O1^xix^—O1—Ba1^xvi^	60.000
O1^vii^—Ba1—O1^iii^	120.000	O1^xix^—O1—Nb1^xv^	45.000
O1^vii^—Ba1—O1^ii^	120.000	O1^xix^—O1—Nb1	135.000
O1^vii^—Ba1—O1^i^	90.000	O1^xix^—O1—Mn1^xv^	45.000
O1^viii^—Ba1—O1^vii^	60.000	O1^xix^—O1—Mn1	135.000
O1^viii^—Ba1—O1^vi^	90.000	O1^viii^—O1—O1^xix^	120.000
O1^viii^—Ba1—O1^v^	60.000	O1^viii^—O1—O1^xviii^	180.000
O1^viii^—Ba1—O1^iv^	120.000	O1^viii^—O1—Ba1^xvii^	120.000
O1^viii^—Ba1—O1^iii^	60.000	O1^viii^—O1—Ba1^xvi^	60.000
O1^viii^—Ba1—O1^ii^	90.000	O1^viii^—O1—Nb1^xv^	135.000
O1^viii^—Ba1—O1^i^	120.000	O1^viii^—O1—Nb1	45.000
O1—Ba1—O1^viii^	60.000	O1^viii^—O1—Mn1^xv^	135.000
O1—Ba1—O1^vii^	60.000	O1^viii^—O1—Mn1	45.000
O1—Ba1—O1^vi^	120.000	O1^vii^—O1—O1^viii^	60.000
O1—Ba1—O1^v^	120.000	O1^vii^—O1—O1^xix^	180.0000 (1)
O1—Ba1—O1^iv^	90.000	O1^vii^—O1—O1^xviii^	120.000
O1—Ba1—O1^iii^	90.000	O1^vii^—O1—Ba1^xvii^	60.000
O1—Ba1—O1^ii^	60.000	O1^vii^—O1—Ba1^xvi^	120.000
O1—Ba1—O1^i^	60.000	O1^vii^—O1—Nb1^xv^	135.000
O1^ix^—Ba1—O1	120.000	O1^vii^—O1—Nb1	45.000
O1^ix^—Ba1—O1^viii^	120.000	O1^vii^—O1—Mn1^xv^	135.000
O1^ix^—Ba1—O1^vii^	180.000	O1^vii^—O1—Mn1	45.000
O1^ix^—Ba1—O1^vi^	120.000	Ba1^xx^—O1—O1^vii^	120.000
O1^ix^—Ba1—O1^v^	90.000	Ba1^xx^—O1—O1^viii^	120.000
O1^ix^—Ba1—O1^iv^	120.000	Ba1^xx^—O1—O1^xix^	60.000
O1^ix^—Ba1—O1^iii^	60.000	Ba1^xx^—O1—O1^xviii^	60.000
O1^ix^—Ba1—O1^ii^	60.000	Ba1^xx^—O1—Ba1^xvii^	90.000
O1^ix^—Ba1—O1^i^	90.000	Ba1^xx^—O1—Ba1^xvi^	90.000
O1^x^—Ba1—O1^ix^	60.000	Ba1^xx^—O1—Nb1^xv^	90.000
O1^x^—Ba1—O1	120.000	Ba1^xx^—O1—Nb1	90.000
O1^x^—Ba1—O1^viii^	180.0000 (1)	Ba1^xx^—O1—Mn1^xv^	90.000
O1^x^—Ba1—O1^vii^	120.000	Ba1^xx^—O1—Mn1	90.000
O1^x^—Ba1—O1^vi^	90.000	O1^xii^—O1—Ba1^xx^	60.000
O1^x^—Ba1—O1^v^	120.000	O1^xii^—O1—O1^vii^	60.000
O1^x^—Ba1—O1^iv^	60.000	O1^xii^—O1—O1^viii^	90.000
O1^x^—Ba1—O1^iii^	120.000	O1^xii^—O1—O1^xix^	120.000
O1^x^—Ba1—O1^ii^	90.000	O1^xii^—O1—O1^xviii^	90.000
O1^x^—Ba1—O1^i^	60.000	O1^xii^—O1—Ba1^xvii^	60.000
O1^xi^—Ba1—O1^x^	60.000	O1^xii^—O1—Ba1^xvi^	120.000
O1^xi^—Ba1—O1^ix^	60.000	O1^xii^—O1—Nb1^xv^	135.000
O1^xi^—Ba1—O1	180.000	O1^xii^—O1—Nb1	45.000
O1^xi^—Ba1—O1^viii^	120.000	O1^xii^—O1—Mn1^xv^	135.000
O1^xi^—Ba1—O1^vii^	120.000	O1^xii^—O1—Mn1	45.000
O1^xi^—Ba1—O1^vi^	60.000	O1^xiii^—O1—O1^xii^	60.000
O1^xi^—Ba1—O1^v^	60.000	O1^xiii^—O1—Ba1^xx^	60.000
O1^xi^—Ba1—O1^iv^	90.000	O1^xiii^—O1—O1^vii^	90.000
O1^xi^—Ba1—O1^iii^	90.000	O1^xiii^—O1—O1^viii^	60.000
O1^xi^—Ba1—O1^ii^	120.000	O1^xiii^—O1—O1^xix^	90.000
O1^xi^—Ba1—O1^i^	120.000	O1^xiii^—O1—O1^xviii^	120.000
O1^xii^—Mn1—Nb1		O1^xiii^—O1—Ba1^xvii^	120.000
O1^xiii^—Mn1—O1^xii^	90.000	O1^xiii^—O1—Ba1^xvi^	60.000
O1^xiii^—Mn1—Nb1		O1^xiii^—O1—Nb1^xv^	135.000
O1^viii^—Mn1—O1^xiii^	90.000	O1^xiii^—O1—Nb1	45.000
O1^viii^—Mn1—O1^xii^	180.0000 (1)	O1^xiii^—O1—Mn1^xv^	135.000
O1^viii^—Mn1—Nb1		O1^xiii^—O1—Mn1	45.000
O1—Mn1—O1^viii^	90.000	O1^ii^—O1—O1^xiii^	120.000
O1—Mn1—O1^xiii^	90.000	O1^ii^—O1—O1^xii^	180.0000 (1)
O1—Mn1—O1^xii^	90.000	O1^ii^—O1—Ba1^xx^	120.000
O1—Mn1—Nb1		O1^ii^—O1—O1^vii^	120.000
O1^xiv^—Mn1—O1	180.000	O1^ii^—O1—O1^viii^	90.000
O1^xiv^—Mn1—O1^viii^	90.000	O1^ii^—O1—O1^xix^	60.000
O1^xiv^—Mn1—O1^xiii^	90.000	O1^ii^—O1—O1^xviii^	90.000
O1^xiv^—Mn1—O1^xii^	90.000	O1^ii^—O1—Ba1^xvii^	120.000
O1^xiv^—Mn1—Nb1		O1^ii^—O1—Ba1^xvi^	60.000
O1^vii^—Mn1—O1^xiv^	90.000	O1^ii^—O1—Nb1^xv^	45.000
O1^vii^—Mn1—O1	90.000	O1^ii^—O1—Nb1	135.000
O1^vii^—Mn1—O1^viii^	90.000	O1^ii^—O1—Mn1^xv^	45.000
O1^vii^—Mn1—O1^xiii^	180.000	O1^ii^—O1—Mn1	135.000
O1^vii^—Mn1—O1^xii^	90.000	O1^i^—O1—O1^ii^	60.000
O1^vii^—Mn1—Nb1		O1^i^—O1—O1^xiii^	180.0000 (1)
O1^xii^—Nb1—Mn1		O1^i^—O1—O1^xii^	120.000
O1^xiii^—Nb1—O1^xii^	90.000	O1^i^—O1—Ba1^xx^	120.000
O1^xiii^—Nb1—Mn1		O1^i^—O1—O1^vii^	90.000
O1^viii^—Nb1—O1^xiii^	90.000	O1^i^—O1—O1^viii^	120.000
O1^viii^—Nb1—O1^xii^	180.0000 (1)	O1^i^—O1—O1^xix^	90.000
O1^viii^—Nb1—Mn1		O1^i^—O1—O1^xviii^	60.000
O1—Nb1—O1^viii^	90.000	O1^i^—O1—Ba1^xvii^	60.000
O1—Nb1—O1^xiii^	90.000	O1^i^—O1—Ba1^xvi^	120.000
O1—Nb1—O1^xii^	90.000	O1^i^—O1—Nb1^xv^	45.000
O1—Nb1—Mn1		O1^i^—O1—Nb1	135.000
O1^xiv^—Nb1—O1	180.000	O1^i^—O1—Mn1^xv^	45.000
O1^xiv^—Nb1—O1^viii^	90.000	O1^i^—O1—Mn1	135.000
O1^xiv^—Nb1—O1^xiii^	90.000	Ba1—O1—O1^i^	60.000
O1^xiv^—Nb1—O1^xii^	90.000	Ba1—O1—O1^ii^	60.000
O1^xiv^—Nb1—Mn1		Ba1—O1—O1^xiii^	120.000
O1^vii^—Nb1—O1^xiv^	90.000	Ba1—O1—O1^xii^	120.000
O1^vii^—Nb1—O1	90.000	Ba1—O1—Ba1^xx^	180.0000 (1)
O1^vii^—Nb1—O1^viii^	90.000	Ba1—O1—O1^vii^	60.000
O1^vii^—Nb1—O1^xiii^	180.000	Ba1—O1—O1^viii^	60.000
O1^vii^—Nb1—O1^xii^	90.000	Ba1—O1—O1^xix^	120.000
O1^vii^—Nb1—Mn1		Ba1—O1—O1^xviii^	120.000
Mn1^xv^—O1—Mn1	180.000	Ba1—O1—Ba1^xvii^	90.000
Nb1—O1—Mn1^xv^	180.000	Ba1—O1—Ba1^xvi^	90.000
Nb1—O1—Mn1	0.000	Ba1—O1—Nb1^xv^	90.000
Nb1^xv^—O1—Nb1	180.000	Ba1—O1—Nb1	90.000
Nb1^xv^—O1—Mn1^xv^	0.000	Ba1—O1—Mn1^xv^	90.000
Nb1^xv^—O1—Mn1	180.000	Ba1—O1—Mn1	90.000

Symmetry codes: (i) −*z*, −*x*+1, −*y*; (ii) −*x*+1, −*z*, −*y*; (iii) *x*, *y*−1, *z*; (iv) *x*−1, *y*, *z*; (v) −*z*, −*x*, −*y*+1; (vi) −*x*, −*z*, −*y*+1; (vii) −*z*, −*x*+1, −*y*+1; (viii) −*x*+1, −*z*, −*y*+1; (ix) −*z*, −*x*, −*y*; (x) −*x*, −*z*, −*y*; (xi) *x*−1, *y*−1, *z*; (xii) −*x*+1, −*z*+1, −*y*+1; (xiii) −*z*+1, −*x*+1, −*y*+1; (xiv) *x*, *y*, *z*+1; (xv) *x*, *y*, *z*−1; (xvi) *x*+1, *y*, *z*; (xvii) *x*, *y*+1, *z*; (xviii) −*x*+1, −*z*+1, −*y*; (xix) −*z*+1, −*x*+1, −*y*; (xx) *x*+1, *y*+1, *z*.

### (barium_niobiumV_manganeseII_oxide) Crystal data

**Table:**

(Ba3MnNb2O_9_)_0.333_	*V* = 559.07 (10) Å^3^
*M**_r_* = 265.58	*Z* = 8
Cubic, *F**m*3*m*	*D*_x_ = 6.310 (1) Mg m^−^^3^
*a* = 8.2380 (5) Å	*T* = 300 K

### (barium_niobiumV_manganeseII_oxide) Data collection

**Table:**

SSRL beam line 2-1 diffractometer	

### (barium_niobiumV_manganeseII_oxide) Fractional atomic coordinates and isotropic or equivalent isotropic displacement parameters (Å^2^)

**Table:**

	*x*	*y*	*z*	*B*_iso_*/*B*_eq_	Occ. (<1)
Ba1	0.25	0.25	0.25	0.48341	
Nb1	0	0	0	0.48341	0.3333
Mn1	0	0	0	0.48341	0.6667
Nb2	0.5	0.5	0.5	0.48341	
O1	0.247	0	0	0.48341	

### (barium_niobiumV_manganeseII_oxide) Geometric parameters (Å, º)

**Table:**

Ba1—O1^i^	2.9127 (2)	Mn1—O1^xiii^	2.0348 (1)
Ba1—O1^ii^	2.9127 (2)	Mn1—O1^xiv^	2.0348 (1)
Ba1—O1^iii^	2.9127 (2)	Nb2—O1^vii^	2.0842 (1)
Ba1—O1^iv^	2.9127 (2)	Nb2—O1^xv^	2.0842 (1)
Ba1—O1^v^	2.9127 (2)	Nb2—O1^viii^	2.0842 (1)
Ba1—O1^vi^	2.9127 (2)	Nb2—O1^xi^	2.0842 (1)
Ba1—O1	2.9127 (2)	Nb2—O1^xvi^	2.0842 (1)
Ba1—O1^vii^	2.9127 (2)	Nb2—O1^xvii^	2.0842 (1)
Ba1—O1^viii^	2.9127 (2)	O1—Nb1	2.0348 (1)
Ba1—O1^ix^	2.9127 (2)	O1—Mn1	2.0348 (1)
Ba1—O1^x^	2.9127 (2)	O1—Nb2^xviii^	2.0842 (1)
Ba1—O1^xi^	2.9127 (2)	O1—O1^ix^	2.8776 (2)
Nb1—Mn1	0.00000	O1—O1^x^	2.8776 (2)
Nb1—O1^ix^	2.0348 (1)	O1—O1^xii^	2.8776 (2)
Nb1—O1^xii^	2.0348 (1)	O1—O1^xiii^	2.8776 (2)
Nb1—O1	2.0348 (1)	O1—Ba1^xix^	2.9127 (2)
Nb1—O1^x^	2.0348 (1)	O1—Ba1^xx^	2.9127 (2)
Nb1—O1^xiii^	2.0348 (1)	O1—Ba1	2.9127 (2)
Nb1—O1^xiv^	2.0348 (1)	O1—Ba1^xxi^	2.9127 (2)
Mn1—Nb1	0.00000	O1—O1^xxii^	2.9475 (2)
Mn1—O1^ix^	2.0348 (1)	O1—O1^xxiii^	2.9475 (2)
Mn1—O1^xii^	2.0348 (1)	O1—O1^vi^	2.9475 (2)
Mn1—O1	2.0348 (1)	O1—O1^ii^	2.9475 (2)
Mn1—O1^x^	2.0348 (1)		
			
O1^ii^—Ba1—O1^i^	59.205	O1^xvi^—Nb2—O1^xi^	90.000
O1^iii^—Ba1—O1^ii^	179.028	O1^xvi^—Nb2—O1^viii^	180.000
O1^iii^—Ba1—O1^i^	119.998	O1^xvi^—Nb2—O1^xv^	90.000
O1^iv^—Ba1—O1^iii^	59.205	O1^xvi^—Nb2—O1^vii^	90.000
O1^iv^—Ba1—O1^ii^	119.998	O1^xvii^—Nb2—O1^xvi^	90.000
O1^iv^—Ba1—O1^i^	60.793	O1^xvii^—Nb2—O1^xi^	180.000
O1^v^—Ba1—O1^iv^	119.998	O1^xvii^—Nb2—O1^viii^	90.000
O1^v^—Ba1—O1^iii^	60.793	O1^xvii^—Nb2—O1^xv^	90.000
O1^v^—Ba1—O1^ii^	119.998	O1^xvii^—Nb2—O1^vii^	90.000
O1^v^—Ba1—O1^i^	179.028	Mn1—O1—Nb1	0.000
O1^vi^—Ba1—O1^v^	59.205	Nb2^xviii^—O1—Mn1	180.000
O1^vi^—Ba1—O1^iv^	179.028	Nb2^xviii^—O1—Nb1	180.000
O1^vi^—Ba1—O1^iii^	119.998	O1^ix^—O1—Nb2^xviii^	135.000
O1^vi^—Ba1—O1^ii^	60.793	O1^ix^—O1—Mn1	45.000
O1^vi^—Ba1—O1^i^	119.998	O1^ix^—O1—Nb1	45.000
O1—Ba1—O1^vi^	60.793	O1^x^—O1—O1^ix^	60.000
O1—Ba1—O1^v^	90.004	O1^x^—O1—Nb2^xviii^	135.000
O1—Ba1—O1^iv^	119.998	O1^x^—O1—Mn1	45.000
O1—Ba1—O1^iii^	119.998	O1^x^—O1—Nb1	45.000
O1—Ba1—O1^ii^	60.793	O1^xii^—O1—O1^x^	90.000
O1—Ba1—O1^i^	90.004	O1^xii^—O1—O1^ix^	60.000
O1^vii^—Ba1—O1	119.998	O1^xii^—O1—Nb2^xviii^	135.000
O1^vii^—Ba1—O1^vi^	90.004	O1^xii^—O1—Mn1	45.000
O1^vii^—Ba1—O1^v^	119.998	O1^xii^—O1—Nb1	45.000
O1^vii^—Ba1—O1^iv^	90.004	O1^xiii^—O1—O1^xii^	60.000
O1^vii^—Ba1—O1^iii^	119.998	O1^xiii^—O1—O1^x^	60.000
O1^vii^—Ba1—O1^ii^	59.205	O1^xiii^—O1—O1^ix^	90.000
O1^vii^—Ba1—O1^i^	59.205	O1^xiii^—O1—Nb2^xviii^	135.000
O1^viii^—Ba1—O1^vii^	60.793	O1^xiii^—O1—Mn1	45.000
O1^viii^—Ba1—O1	179.028	O1^xiii^—O1—Nb1	45.000
O1^viii^—Ba1—O1^vi^	119.998	Ba1^xix^—O1—O1^xiii^	120.397
O1^viii^—Ba1—O1^v^	90.004	Ba1^xix^—O1—O1^xii^	60.397
O1^viii^—Ba1—O1^iv^	59.205	Ba1^xix^—O1—O1^x^	120.397
O1^viii^—Ba1—O1^iii^	59.205	Ba1^xix^—O1—O1^ix^	60.397
O1^viii^—Ba1—O1^ii^	119.998	Ba1^xix^—O1—Nb2^xviii^	89.514
O1^viii^—Ba1—O1^i^	90.004	Ba1^xix^—O1—Mn1	90.486
O1^ix^—Ba1—O1^viii^	119.998	Ba1^xix^—O1—Nb1	90.486
O1^ix^—Ba1—O1^vii^	179.028	Ba1^xx^—O1—Ba1^xix^	179.028
O1^ix^—Ba1—O1	59.205	Ba1^xx^—O1—O1^xiii^	60.397
O1^ix^—Ba1—O1^vi^	90.004	Ba1^xx^—O1—O1^xii^	120.397
O1^ix^—Ba1—O1^v^	60.793	Ba1^xx^—O1—O1^x^	60.397
O1^ix^—Ba1—O1^iv^	90.004	Ba1^xx^—O1—O1^ix^	120.397
O1^ix^—Ba1—O1^iii^	60.793	Ba1^xx^—O1—Nb2^xviii^	89.514
O1^ix^—Ba1—O1^ii^	119.998	Ba1^xx^—O1—Mn1	90.486
O1^ix^—Ba1—O1^i^	119.998	Ba1^xx^—O1—Nb1	90.486
O1^x^—Ba1—O1^ix^	59.205	Ba1—O1—Ba1^xx^	89.996
O1^x^—Ba1—O1^viii^	119.998	Ba1—O1—Ba1^xix^	89.996
O1^x^—Ba1—O1^vii^	119.998	Ba1—O1—O1^xiii^	120.397
O1^x^—Ba1—O1	59.205	Ba1—O1—O1^xii^	120.397
O1^x^—Ba1—O1^vi^	119.998	Ba1—O1—O1^x^	60.397
O1^x^—Ba1—O1^v^	119.998	Ba1—O1—O1^ix^	60.397
O1^x^—Ba1—O1^iv^	60.793	Ba1—O1—Nb2^xviii^	89.514
O1^x^—Ba1—O1^iii^	90.004	Ba1—O1—Mn1	90.486
O1^x^—Ba1—O1^ii^	90.004	Ba1—O1—Nb1	90.486
O1^x^—Ba1—O1^i^	60.793	Ba1^xxi^—O1—Ba1	179.028
O1^xi^—Ba1—O1^x^	179.028	Ba1^xxi^—O1—Ba1^xx^	89.996
O1^xi^—Ba1—O1^ix^	119.998	Ba1^xxi^—O1—Ba1^xix^	89.996
O1^xi^—Ba1—O1^viii^	60.793	Ba1^xxi^—O1—O1^xiii^	60.397
O1^xi^—Ba1—O1^vii^	60.793	Ba1^xxi^—O1—O1^xii^	60.397
O1^xi^—Ba1—O1	119.998	Ba1^xxi^—O1—O1^x^	120.397
O1^xi^—Ba1—O1^vi^	59.205	Ba1^xxi^—O1—O1^ix^	120.397
O1^xi^—Ba1—O1^v^	59.205	Ba1^xxi^—O1—Nb2^xviii^	89.514
O1^xi^—Ba1—O1^iv^	119.998	Ba1^xxi^—O1—Mn1	90.486
O1^xi^—Ba1—O1^iii^	90.004	Ba1^xxi^—O1—Nb1	90.486
O1^xi^—Ba1—O1^ii^	90.004	O1^xxii^—O1—Ba1^xxi^	59.603
O1^xi^—Ba1—O1^i^	119.998	O1^xxii^—O1—Ba1	119.603
O1^ix^—Nb1—Mn1		O1^xxii^—O1—Ba1^xx^	59.603
O1^xii^—Nb1—O1^ix^	90.000	O1^xxii^—O1—Ba1^xix^	119.603
O1^xii^—Nb1—Mn1		O1^xxii^—O1—O1^xiii^	90.000
O1—Nb1—O1^xii^	90.000	O1^xxii^—O1—O1^xii^	120.000
O1—Nb1—O1^ix^	90.000	O1^xxii^—O1—O1^x^	120.000
O1—Nb1—Mn1		O1^xxii^—O1—O1^ix^	180.000
O1^x^—Nb1—O1	90.000	O1^xxii^—O1—Nb2^xviii^	45.000
O1^x^—Nb1—O1^xii^	180.000	O1^xxii^—O1—Mn1	135.000
O1^x^—Nb1—O1^ix^	90.000	O1^xxii^—O1—Nb1	135.000
O1^x^—Nb1—Mn1		O1^xxiii^—O1—O1^xxii^	60.000
O1^xiii^—Nb1—O1^x^	90.000	O1^xxiii^—O1—Ba1^xxi^	59.603
O1^xiii^—Nb1—O1	90.000	O1^xxiii^—O1—Ba1	119.603
O1^xiii^—Nb1—O1^xii^	90.000	O1^xxiii^—O1—Ba1^xx^	119.603
O1^xiii^—Nb1—O1^ix^	180.000	O1^xxiii^—O1—Ba1^xix^	59.603
O1^xiii^—Nb1—Mn1		O1^xxiii^—O1—O1^xiii^	120.000
O1^xiv^—Nb1—O1^xiii^	90.000	O1^xxiii^—O1—O1^xii^	90.000
O1^xiv^—Nb1—O1^x^	90.000	O1^xxiii^—O1—O1^x^	180.000
O1^xiv^—Nb1—O1	180.000	O1^xxiii^—O1—O1^ix^	120.000
O1^xiv^—Nb1—O1^xii^	90.000	O1^xxiii^—O1—Nb2^xviii^	45.000
O1^xiv^—Nb1—O1^ix^	90.000	O1^xxiii^—O1—Mn1	135.000
O1^xiv^—Nb1—Mn1		O1^xxiii^—O1—Nb1	135.000
O1^ix^—Mn1—Nb1		O1^vi^—O1—O1^xxiii^	60.000
O1^xii^—Mn1—O1^ix^	90.000	O1^vi^—O1—O1^xxii^	90.000
O1^xii^—Mn1—Nb1		O1^vi^—O1—Ba1^xxi^	119.603
O1—Mn1—O1^xii^	90.000	O1^vi^—O1—Ba1	59.603
O1—Mn1—O1^ix^	90.000	O1^vi^—O1—Ba1^xx^	119.603
O1—Mn1—Nb1		O1^vi^—O1—Ba1^xix^	59.603
O1^x^—Mn1—O1	90.000	O1^vi^—O1—O1^xiii^	180.000
O1^x^—Mn1—O1^xii^	180.000	O1^vi^—O1—O1^xii^	120.000
O1^x^—Mn1—O1^ix^	90.000	O1^vi^—O1—O1^x^	120.000
O1^x^—Mn1—Nb1		O1^vi^—O1—O1^ix^	90.000
O1^xiii^—Mn1—O1^x^	90.000	O1^vi^—O1—Nb2^xviii^	45.000
O1^xiii^—Mn1—O1	90.000	O1^vi^—O1—Mn1	135.000
O1^xiii^—Mn1—O1^xii^	90.000	O1^vi^—O1—Nb1	135.000
O1^xiii^—Mn1—O1^ix^	180.000	O1^ii^—O1—O1^vi^	60.000
O1^xiii^—Mn1—Nb1		O1^ii^—O1—O1^xxiii^	90.000
O1^xiv^—Mn1—O1^xiii^	90.000	O1^ii^—O1—O1^xxii^	60.000
O1^xiv^—Mn1—O1^x^	90.000	O1^ii^—O1—Ba1^xxi^	119.603
O1^xiv^—Mn1—O1	180.000	O1^ii^—O1—Ba1	59.603
O1^xiv^—Mn1—O1^xii^	90.000	O1^ii^—O1—Ba1^xx^	59.603
O1^xiv^—Mn1—O1^ix^	90.000	O1^ii^—O1—Ba1^xix^	119.603
O1^xiv^—Mn1—Nb1		O1^ii^—O1—O1^xiii^	120.000
O1^xv^—Nb2—O1^vii^	180.000	O1^ii^—O1—O1^xii^	180.0000 (1)
O1^viii^—Nb2—O1^xv^	90.000	O1^ii^—O1—O1^x^	90.000
O1^viii^—Nb2—O1^vii^	90.000	O1^ii^—O1—O1^ix^	120.000
O1^xi^—Nb2—O1^viii^	90.000	O1^ii^—O1—Nb2^xviii^	45.000
O1^xi^—Nb2—O1^xv^	90.000	O1^ii^—O1—Mn1	135.000
O1^xi^—Nb2—O1^vii^	90.000	O1^ii^—O1—Nb1	135.000

Symmetry codes: (i) −*x*+1/2, −*y*, *z*+1/2; (ii) −*y*+1/2, −*z*, −*x*+1/2; (iii) −*y*, −*z*+1/2, −*x*+1/2; (iv) −*y*, −*x*+1/2, −*z*+1/2; (v) −*x*+1/2, −*y*+1/2, *z*; (vi) −*y*+1/2, −*x*+1/2, −*z*; (vii) −*y*+1/2, *x*, −*z*+1/2; (viii) *x*, *y*+1/2, *z*+1/2; (ix) −*y*, *x*, −*z*; (x) −*y*, −*z*, *x*; (xi) −*y*+1/2, −*z*+1/2, *x*; (xii) −*y*, −*z*, −*x*; (xiii) −*y*, −*x*, −*z*; (xiv) −*x*, −*y*, *z*; (xv) −*y*+1/2, −*x*+1, −*z*+1/2; (xvi) −*x*+1, −*y*+1/2, *z*+1/2; (xvii) −*y*+1/2, −*z*+1/2, −*x*+1; (xviii) *x*, *y*−1/2, *z*−1/2; (xix) *z*, *y*, −*x*; (xx) *z*, −*x*, *y*; (xxi) *z*, −*x*, −*y*; (xxii) −*y*+1/2, *x*−1/2, −*z*; (xxiii) −*y*+1/2, −*z*, *x*−1/2.

### (12R-barium_niobium_manganese_oxide) Crystal data

**Table:**

Ba_4_NbMn_3_O_12_	*Z* = 3
*M**_r_* = 999.02	*D*_x_ = 6.209 (1) Mg m^−^^3^
Hexagonal, *R*3*m*	Synchrotron X-ray radiation, λ = 0.729277 Å
*a* = 5.73256 (3) Å	µ = 20.22 mm^−^^1^
*c* = 28.16620 (19) Å	*T* = 300 K
*V* = 801.60 (1) Å^3^	cylinder, 1 × 0.5 mm

### (12R-barium_niobium_manganese_oxide) Data collection

**Table:**

SSRL beam line 2-1 diffractometer	Scan method: step
Specimen mounting: Glass capillary	2θ_min_ = 8.5°, 2θ_max_ = 84°, 2θ_step_ = 0.005°
Data collection mode: transmission	

### (12R-barium_niobium_manganese_oxide) Refinement

**Table:**

*R*_p_ = 0.062	36 parameters
*R*_wp_ = 0.087	0 restraints
*R*_exp_ = 0.167	

### (12R-barium_niobium_manganese_oxide) Fractional atomic coordinates and isotropic or equivalent isotropic displacement parameters (Å^2^)

**Table:**

	*x*	*y*	*z*	*B*_iso_*/*B*_eq_	
Ba1	0	0	0.12960 (3)	0.61 (9)	
Ba2	0	0	0.28541 (3)	0.61 (9)	
Nb3	0	0	0	0.43 (8)	
Mn1	0	0	0.5	0.43 (8)	
Mn5	0	0	0.41098 (7)	0.43 (8)	
O1	0.47778	0.52222	0.12374 (16)	0.65 (10)	
O2	0.50188	0.49812	0.29246 (19)	0.65 (10)	

### (12R-barium_niobium_manganese_oxide) Geometric parameters (Å, º)

**Table:**

Ba1—O1^i^	2.8795 (2)	Mn1—Mn5^xviii^	2.5074 (18)
Ba1—O1^ii^	2.8795 (2)	Mn1—Mn5	2.5074 (18)
Ba1—O1^iii^	2.8795 (2)	Mn5—O1^xvii^	1.935 (3)
Ba1—O1^iv^	2.8795 (2)	Mn5—O1^xv^	1.935 (3)
Ba1—O1	2.8795 (2)	Mn5—O1^xvi^	1.935 (3)
Ba1—O1^v^	2.8795 (2)	Mn5—O2^x^	1.968 (3)
Ba1—O1^vi^	2.932 (4)	Mn5—O2^xi^	1.968 (3)
Ba1—O1^vii^	2.932 (4)	Mn5—O2^ix^	1.968 (3)
Ba1—O1^viii^	2.932 (4)	Mn5—Mn1	2.5074 (18)
Ba1—O2^viii^	2.987 (5)	O1—Mn1^xix^	1.876 (3)
Ba1—O2^vii^	2.987 (5)	O1—Mn5^xix^	1.935 (3)
Ba1—O2^vi^	2.987 (5)	O1—O1^xx^	2.4842 (1)
Ba2—O1^viii^	2.842 (4)	O1—O1^iii^	2.4842 (1)
Ba2—O1^vii^	2.842 (4)	O1—O1^viii^	2.811 (8)
Ba2—O1^vi^	2.842 (4)	O1—O1^xxi^	2.811 (8)
Ba2—O2^i^	2.8732 (5)	O1—O2^xxi^	2.812 (6)
Ba2—O2^iii^	2.8732 (5)	O1—O2^viii^	2.812 (6)
Ba2—O2^iv^	2.8732 (5)	O1—Ba2^vii^	2.842 (4)
Ba2—O2^ii^	2.8732 (5)	O1—Ba1	2.8795 (2)
Ba2—O2^v^	2.8732 (5)	O1—Ba1^xxii^	2.8795 (2)
Ba2—O2	2.8732 (5)	O1—Ba1^vii^	2.932 (4)
Ba2—O2^ix^	3.009 (5)	O2—Mn5^x^	1.968 (3)
Ba2—O2^x^	3.009 (5)	O2—Nb3^xxiii^	2.001 (3)
Ba2—O2^xi^	3.009 (5)	O2—O1^xxi^	2.812 (6)
Nb3—O2^vii^	2.001 (3)	O2—O1^viii^	2.812 (6)
Nb3—O2^vi^	2.001 (3)	O2—O2^xi^	2.825 (9)
Nb3—O2^xii^	2.001 (3)	O2—O2^xxiv^	2.825 (9)
Nb3—O2^xiii^	2.001 (3)	O2—O2^ii^	2.8340 (1)
Nb3—O2^viii^	2.001 (3)	O2—O2^xxv^	2.8340 (1)
Nb3—O2^xiv^	2.001 (3)	O2—Ba2^xxii^	2.8732 (5)
Mn1—O1^ix^	1.876 (3)	O2—Ba2	2.8732 (5)
Mn1—O1^xv^	1.876 (3)	O2—O2^iii^	2.8986 (1)
Mn1—O1^x^	1.876 (3)	O2—O2^xx^	2.8986 (1)
Mn1—O1^xvi^	1.876 (3)	O2—Ba1^vii^	2.987 (5)
Mn1—O1^xvii^	1.876 (3)	O2—Ba2^x^	3.009 (5)
Mn1—O1^xi^	1.876 (3)		
			
O1^ii^—Ba1—O1^i^	51.107 (4)	O1^xvi^—Mn5—O1^xvii^	79.89 (16)
O1^iii^—Ba1—O1^ii^	119.674 (13)	O2^x^—Mn5—O1^xvi^	92.18 (12)
O1^iii^—Ba1—O1^i^	169.02 (8)	O2^x^—Mn5—O1^xv^	169.60 (18)
O1^iv^—Ba1—O1^iii^	68.674 (5)	O2^x^—Mn5—O1^xvii^	92.18 (12)
O1^iv^—Ba1—O1^ii^	169.02 (8)	O2^xi^—Mn5—O2^x^	94.85 (19)
O1^iv^—Ba1—O1^i^	119.674 (13)	O2^xi^—Mn5—O1^xvi^	92.18 (12)
O1—Ba1—O1^iv^	119.674 (13)	O2^xi^—Mn5—O1^xv^	92.18 (12)
O1—Ba1—O1^iii^	51.107 (4)	O2^xi^—Mn5—O1^xvii^	169.60 (18)
O1—Ba1—O1^ii^	68.674 (5)	O2^ix^—Mn5—O2^xi^	94.85 (19)
O1—Ba1—O1^i^	119.674 (13)	O2^ix^—Mn5—O2^x^	94.85 (19)
O1^v^—Ba1—O1	169.02 (8)	O2^ix^—Mn5—O1^xvi^	169.60 (18)
O1^v^—Ba1—O1^iv^	51.107 (4)	O2^ix^—Mn5—O1^xv^	92.18 (12)
O1^v^—Ba1—O1^iii^	119.674 (13)	O2^ix^—Mn5—O1^xvii^	92.18 (12)
O1^v^—Ba1—O1^ii^	119.674 (13)	Mn1—Mn5—O2^ix^	121.75 (14)
O1^v^—Ba1—O1^i^	68.674 (5)	Mn1—Mn5—O2^xi^	121.75 (14)
O1^vi^—Ba1—O1^v^	57.86 (14)	Mn1—Mn5—O2^x^	121.75 (14)
O1^vi^—Ba1—O1	124.82 (4)	Mn1—Mn5—O1^xvi^	47.85 (11)
O1^vi^—Ba1—O1^iv^	57.86 (14)	Mn1—Mn5—O1^xv^	47.85 (11)
O1^vi^—Ba1—O1^iii^	95.34 (7)	Mn1—Mn5—O1^xvii^	47.85 (11)
O1^vi^—Ba1—O1^ii^	124.82 (4)	Mn5^xix^—O1—Mn1^xix^	82.28 (4)
O1^vi^—Ba1—O1^i^	95.34 (7)	O1^xx^—O1—Mn5^xix^	50.06 (8)
O1^vii^—Ba1—O1^vi^	67.29 (9)	O1^xx^—O1—Mn1^xix^	48.54 (8)
O1^vii^—Ba1—O1^v^	95.34 (7)	O1^iii^—O1—O1^xx^	60.000
O1^vii^—Ba1—O1	95.34 (7)	O1^iii^—O1—Mn5^xix^	50.06 (8)
O1^vii^—Ba1—O1^iv^	124.82 (4)	O1^iii^—O1—Mn1^xix^	48.54 (8)
O1^vii^—Ba1—O1^iii^	124.82 (4)	O1^viii^—O1—O1^iii^	63.78 (8)
O1^vii^—Ba1—O1^ii^	57.86 (14)	O1^viii^—O1—O1^xx^	90.000
O1^vii^—Ba1—O1^i^	57.86 (14)	O1^viii^—O1—Mn5^xix^	112.84 (4)
O1^viii^—Ba1—O1^vii^	67.29 (9)	O1^viii^—O1—Mn1^xix^	41.46 (8)
O1^viii^—Ba1—O1^vi^	67.29 (9)	O1^xxi^—O1—O1^viii^	52.44 (17)
O1^viii^—Ba1—O1^v^	124.82 (4)	O1^xxi^—O1—O1^iii^	90.000
O1^viii^—Ba1—O1	57.86 (14)	O1^xxi^—O1—O1^xx^	63.78 (8)
O1^viii^—Ba1—O1^iv^	95.34 (7)	O1^xxi^—O1—Mn5^xix^	112.84 (4)
O1^viii^—Ba1—O1^iii^	57.86 (14)	O1^xxi^—O1—Mn1^xix^	41.46 (8)
O1^viii^—Ba1—O1^ii^	95.34 (7)	O2^xxi^—O1—O1^xxi^	115.60 (8)
O1^viii^—Ba1—O1^i^	124.82 (4)	O2^xxi^—O1—O1^viii^	152.56 (3)
O2^viii^—Ba1—O1^viii^	107.01 (8)	O2^xxi^—O1—O1^iii^	94.227 (9)
O2^viii^—Ba1—O1^vii^	144.91 (4)	O2^xxi^—O1—O1^xx^	63.78 (6)
O2^viii^—Ba1—O1^vi^	144.91 (4)	O2^xxi^—O1—Mn5^xix^	44.39 (8)
O2^viii^—Ba1—O1^v^	113.78 (10)	O2^xxi^—O1—Mn1^xix^	111.88 (7)
O2^viii^—Ba1—O1	57.24 (10)	O2^viii^—O1—O2^xxi^	62.06 (14)
O2^viii^—Ba1—O1^iv^	89.66 (8)	O2^viii^—O1—O1^xxi^	152.56 (3)
O2^viii^—Ba1—O1^iii^	57.24 (10)	O2^viii^—O1—O1^viii^	115.60 (8)
O2^viii^—Ba1—O1^ii^	89.66 (8)	O2^viii^—O1—O1^iii^	63.78 (6)
O2^viii^—Ba1—O1^i^	113.78 (10)	O2^viii^—O1—O1^xx^	94.227 (9)
O2^vii^—Ba1—O2^viii^	56.64 (9)	O2^viii^—O1—Mn5^xix^	44.39 (8)
O2^vii^—Ba1—O1^viii^	144.91 (4)	O2^viii^—O1—Mn1^xix^	111.88 (7)
O2^vii^—Ba1—O1^vii^	107.01 (8)	Ba2^vii^—O1—O2^viii^	61.09 (10)
O2^vii^—Ba1—O1^vi^	144.91 (4)	Ba2^vii^—O1—O2^xxi^	61.09 (10)
O2^vii^—Ba1—O1^v^	89.66 (8)	Ba2^vii^—O1—O1^xxi^	144.544 (16)
O2^vii^—Ba1—O1	89.66 (8)	Ba2^vii^—O1—O1^viii^	144.544 (16)
O2^vii^—Ba1—O1^iv^	113.78 (10)	Ba2^vii^—O1—O1^iii^	124.85 (5)
O2^vii^—Ba1—O1^iii^	113.78 (10)	Ba2^vii^—O1—O1^xx^	124.85 (5)
O2^vii^—Ba1—O1^ii^	57.24 (10)	Ba2^vii^—O1—Mn5^xix^	89.14 (16)
O2^vii^—Ba1—O1^i^	57.24 (10)	Ba2^vii^—O1—Mn1^xix^	171.42 (17)
O2^vi^—Ba1—O2^vii^	56.64 (9)	Ba1—O1—Ba2^vii^	89.57 (6)
O2^vi^—Ba1—O2^viii^	56.64 (9)	Ba1—O1—O2^viii^	63.30 (7)
O2^vi^—Ba1—O1^viii^	144.91 (4)	Ba1—O1—O2^xxi^	125.24 (15)
O2^vi^—Ba1—O1^vii^	144.91 (4)	Ba1—O1—O1^xxi^	114.20 (18)
O2^vi^—Ba1—O1^vi^	107.01 (8)	Ba1—O1—O1^viii^	61.997 (16)
O2^vi^—Ba1—O1^v^	57.24 (10)	Ba1—O1—O1^iii^	64.4466 (18)
O2^vi^—Ba1—O1	113.78 (10)	Ba1—O1—O1^xx^	124.337 (3)
O2^vi^—Ba1—O1^iv^	57.24 (10)	Ba1—O1—Mn5^xix^	95.47 (6)
O2^vi^—Ba1—O1^iii^	89.66 (8)	Ba1—O1—Mn1^xix^	91.24 (7)
O2^vi^—Ba1—O1^ii^	113.78 (10)	Ba1^xxii^—O1—Ba1	169.02 (8)
O2^vi^—Ba1—O1^i^	89.66 (8)	Ba1^xxii^—O1—Ba2^vii^	89.57 (6)
O1^vii^—Ba2—O1^viii^	69.71 (10)	Ba1^xxii^—O1—O2^viii^	125.24 (15)
O1^vi^—Ba2—O1^vii^	69.71 (10)	Ba1^xxii^—O1—O2^xxi^	63.30 (7)
O1^vi^—Ba2—O1^viii^	69.71 (10)	Ba1^xxii^—O1—O1^xxi^	61.997 (16)
O2^i^—Ba2—O1^vi^	92.73 (8)	Ba1^xxii^—O1—O1^viii^	114.20 (18)
O2^i^—Ba2—O1^vii^	58.93 (11)	Ba1^xxii^—O1—O1^iii^	124.337 (3)
O2^i^—Ba2—O1^viii^	128.62 (11)	Ba1^xxii^—O1—O1^xx^	64.4466 (18)
O2^iii^—Ba2—O2^i^	172.0 (2)	Ba1^xxii^—O1—Mn5^xix^	95.47 (6)
O2^iii^—Ba2—O1^vi^	92.73 (8)	Ba1^xxii^—O1—Mn1^xix^	91.24 (7)
O2^iii^—Ba2—O1^vii^	128.62 (11)	Ba1^vii^—O1—Ba1^xxii^	84.66 (7)
O2^iii^—Ba2—O1^viii^	58.93 (11)	Ba1^vii^—O1—Ba1	84.66 (7)
O2^iv^—Ba2—O2^iii^	59.098 (11)	Ba1^vii^—O1—Ba2^vii^	98.934 (14)
O2^iv^—Ba2—O2^i^	119.53 (3)	Ba1^vii^—O1—O2^viii^	140.71 (3)
O2^iv^—Ba2—O1^vi^	58.93 (11)	Ba1^vii^—O1—O2^xxi^	140.71 (3)
O2^iv^—Ba2—O1^vii^	128.62 (11)	Ba1^vii^—O1—O1^xxi^	60.14 (13)
O2^iv^—Ba2—O1^viii^	92.73 (8)	Ba1^vii^—O1—O1^viii^	60.14 (13)
O2^ii^—Ba2—O2^iv^	172.0 (2)	Ba1^vii^—O1—O1^iii^	123.65 (5)
O2^ii^—Ba2—O2^iii^	119.53 (3)	Ba1^vii^—O1—O1^xx^	123.65 (5)
O2^ii^—Ba2—O2^i^	60.586 (11)	Ba1^vii^—O1—Mn5^xix^	171.93 (16)
O2^ii^—Ba2—O1^vi^	128.62 (11)	Ba1^vii^—O1—Mn1^xix^	89.64 (17)
O2^ii^—Ba2—O1^vii^	58.93 (11)	Nb3^xxiii^—O2—Mn5^x^	176.62 (11)
O2^ii^—Ba2—O1^viii^	92.73 (8)	O1^xxi^—O2—Nb3^xxiii^	139.11 (11)
O2^v^—Ba2—O2^ii^	119.53 (3)	O1^xxi^—O2—Mn5^x^	43.44 (10)
O2^v^—Ba2—O2^iv^	60.586 (11)	O1^viii^—O2—O1^xxi^	52.43 (12)
O2^v^—Ba2—O2^iii^	119.53 (3)	O1^viii^—O2—Nb3^xxiii^	139.11 (11)
O2^v^—Ba2—O2^i^	59.098 (11)	O1^viii^—O2—Mn5^x^	43.44 (10)
O2^v^—Ba2—O1^vi^	58.93 (11)	O2^xi^—O2—O1^viii^	123.61 (7)
O2^v^—Ba2—O1^vii^	92.73 (8)	O2^xi^—O2—O1^xxi^	175.43 (12)
O2^v^—Ba2—O1^viii^	128.62 (11)	O2^xi^—O2—Nb3^xxiii^	45.09 (9)
O2—Ba2—O2^v^	172.0 (2)	O2^xi^—O2—Mn5^x^	132.47 (10)
O2—Ba2—O2^ii^	59.098 (11)	O2^xxiv^—O2—O2^xi^	60.2 (2)
O2—Ba2—O2^iv^	119.53 (3)	O2^xxiv^—O2—O1^viii^	175.43 (12)
O2—Ba2—O2^iii^	60.586 (11)	O2^xxiv^—O2—O1^xxi^	123.61 (7)
O2—Ba2—O2^i^	119.53 (3)	O2^xxiv^—O2—Nb3^xxiii^	45.09 (9)
O2—Ba2—O1^vi^	128.62 (11)	O2^xxiv^—O2—Mn5^x^	132.47 (10)
O2—Ba2—O1^vii^	92.73 (8)	O2^ii^—O2—O2^xxiv^	90.000
O2—Ba2—O1^viii^	58.93 (11)	O2^ii^—O2—O2^xi^	59.89 (11)
O2^ix^—Ba2—O2	114.91 (6)	O2^ii^—O2—O1^viii^	94.227 (9)
O2^ix^—Ba2—O2^v^	57.34 (15)	O2^ii^—O2—O1^xxi^	121.03 (7)
O2^ix^—Ba2—O2^ii^	86.91 (9)	O2^ii^—O2—Nb3^xxiii^	44.91 (9)
O2^ix^—Ba2—O2^iv^	86.91 (9)	O2^ii^—O2—Mn5^x^	137.43 (10)
O2^ix^—Ba2—O2^iii^	114.91 (6)	O2^xxv^—O2—O2^ii^	60.000
O2^ix^—Ba2—O2^i^	57.34 (15)	O2^xxv^—O2—O2^xxiv^	59.89 (11)
O2^ix^—Ba2—O1^vi^	116.17 (7)	O2^xxv^—O2—O2^xi^	90.000
O2^ix^—Ba2—O1^vii^	116.17 (7)	O2^xxv^—O2—O1^viii^	121.03 (7)
O2^ix^—Ba2—O1^viii^	172.49 (9)	O2^xxv^—O2—O1^xxi^	94.227 (9)
O2^x^—Ba2—O2^ix^	57.58 (10)	O2^xxv^—O2—Nb3^xxiii^	44.91 (9)
O2^x^—Ba2—O2	86.91 (9)	O2^xxv^—O2—Mn5^x^	137.43 (10)
O2^x^—Ba2—O2^v^	86.91 (9)	Ba2^xxii^—O2—O2^xxv^	60.451 (5)
O2^x^—Ba2—O2^ii^	114.91 (6)	Ba2^xxii^—O2—O2^ii^	120.293 (5)
O2^x^—Ba2—O2^iv^	57.34 (15)	Ba2^xxii^—O2—O2^xxiv^	63.755 (18)
O2^x^—Ba2—O2^iii^	57.34 (15)	Ba2^xxii^—O2—O2^xi^	124.0 (2)
O2^x^—Ba2—O2^i^	114.91 (6)	Ba2^xxii^—O2—O1^viii^	112.40 (15)
O2^x^—Ba2—O1^vi^	116.17 (7)	Ba2^xxii^—O2—O1^xxi^	59.98 (7)
O2^x^—Ba2—O1^vii^	172.49 (9)	Ba2^xxii^—O2—Nb3^xxiii^	92.58 (5)
O2^x^—Ba2—O1^viii^	116.17 (7)	Ba2^xxii^—O2—Mn5^x^	87.60 (7)
O2^xi^—Ba2—O2^x^	57.58 (10)	Ba2—O2—Ba2^xxii^	172.0 (2)
O2^xi^—Ba2—O2^ix^	57.58 (10)	Ba2—O2—O2^xxv^	120.293 (5)
O2^xi^—Ba2—O2	57.34 (15)	Ba2—O2—O2^ii^	60.451 (5)
O2^xi^—Ba2—O2^v^	114.91 (6)	Ba2—O2—O2^xxiv^	124.0 (2)
O2^xi^—Ba2—O2^ii^	57.34 (15)	Ba2—O2—O2^xi^	63.755 (18)
O2^xi^—Ba2—O2^iv^	114.91 (6)	Ba2—O2—O1^viii^	59.98 (7)
O2^xi^—Ba2—O2^iii^	86.91 (9)	Ba2—O2—O1^xxi^	112.40 (15)
O2^xi^—Ba2—O2^i^	86.91 (9)	Ba2—O2—Nb3^xxiii^	92.58 (5)
O2^xi^—Ba2—O1^vi^	172.49 (9)	Ba2—O2—Mn5^x^	87.60 (7)
O2^xi^—Ba2—O1^vii^	116.17 (7)	O2^iii^—O2—Ba2	59.707 (5)
O2^xi^—Ba2—O1^viii^	116.17 (7)	O2^iii^—O2—Ba2^xxii^	119.549 (5)
O2^vi^—Nb3—O2^vii^	90.19 (18)	O2^iii^—O2—O2^xxv^	180.000
O2^xii^—Nb3—O2^vi^	89.81 (18)	O2^iii^—O2—O2^ii^	120.000
O2^xii^—Nb3—O2^vii^	180.000	O2^iii^—O2—O2^xxiv^	120.11 (11)
O2^xiii^—Nb3—O2^xii^	90.19 (18)	O2^iii^—O2—O2^xi^	90.000
O2^xiii^—Nb3—O2^vi^	180.000	O2^iii^—O2—O1^viii^	58.97 (7)
O2^xiii^—Nb3—O2^vii^	89.81 (18)	O2^iii^—O2—O1^xxi^	85.773 (9)
O2^viii^—Nb3—O2^xiii^	89.81 (18)	O2^iii^—O2—Nb3^xxiii^	135.09 (9)
O2^viii^—Nb3—O2^xii^	89.81 (18)	O2^iii^—O2—Mn5^x^	42.57 (10)
O2^viii^—Nb3—O2^vi^	90.19 (18)	O2^xx^—O2—O2^iii^	60.000
O2^viii^—Nb3—O2^vii^	90.19 (18)	O2^xx^—O2—Ba2	119.549 (5)
O2^xiv^—Nb3—O2^viii^	180.0000 (1)	O2^xx^—O2—Ba2^xxii^	59.707 (5)
O2^xiv^—Nb3—O2^xiii^	90.19 (18)	O2^xx^—O2—O2^xxv^	120.000
O2^xiv^—Nb3—O2^xii^	90.19 (18)	O2^xx^—O2—O2^ii^	180.000
O2^xiv^—Nb3—O2^vi^	89.81 (18)	O2^xx^—O2—O2^xxiv^	90.000
O2^xiv^—Nb3—O2^vii^	89.81 (18)	O2^xx^—O2—O2^xi^	120.11 (11)
O1^xv^—Mn1—O1^ix^	97.07 (16)	O2^xx^—O2—O1^viii^	85.773 (9)
O1^x^—Mn1—O1^xv^	180.0000 (1)	O2^xx^—O2—O1^xxi^	58.97 (7)
O1^x^—Mn1—O1^ix^	82.93 (16)	O2^xx^—O2—Nb3^xxiii^	135.09 (9)
O1^xvi^—Mn1—O1^x^	97.07 (16)	O2^xx^—O2—Mn5^x^	42.57 (10)
O1^xvi^—Mn1—O1^xv^	82.93 (16)	Ba1^vii^—O2—O2^xx^	118.32 (5)
O1^xvi^—Mn1—O1^ix^	180.000	Ba1^vii^—O2—O2^iii^	118.32 (5)
O1^xvii^—Mn1—O1^xvi^	82.93 (16)	Ba1^vii^—O2—Ba2	86.89 (10)
O1^xvii^—Mn1—O1^x^	97.07 (16)	Ba1^vii^—O2—Ba2^xxii^	86.89 (10)
O1^xvii^—Mn1—O1^xv^	82.93 (16)	Ba1^vii^—O2—O2^xxv^	61.68 (5)
O1^xvii^—Mn1—O1^ix^	97.07 (16)	Ba1^vii^—O2—O2^ii^	61.68 (5)
O1^xi^—Mn1—O1^xvii^	180.0000 (1)	Ba1^vii^—O2—O2^xxiv^	121.56 (7)
O1^xi^—Mn1—O1^xvi^	97.07 (16)	Ba1^vii^—O2—O2^xi^	121.56 (7)
O1^xi^—Mn1—O1^x^	82.93 (16)	Ba1^vii^—O2—O1^viii^	59.46 (11)
O1^xi^—Mn1—O1^xv^	97.07 (16)	Ba1^vii^—O2—O1^xxi^	59.46 (11)
O1^xi^—Mn1—O1^ix^	82.93 (16)	Ba1^vii^—O2—Nb3^xxiii^	91.92 (8)
Mn5^xviii^—Mn1—O1^xi^	49.87 (11)	Ba1^vii^—O2—Mn5^x^	91.46 (19)
Mn5^xviii^—Mn1—O1^xvii^	130.13 (11)	Ba2^x^—O2—Ba1^vii^	179.43 (12)
Mn5^xviii^—Mn1—O1^xvi^	130.13 (11)	Ba2^x^—O2—O2^xx^	61.21 (5)
Mn5^xviii^—Mn1—O1^x^	49.87 (11)	Ba2^x^—O2—O2^iii^	61.21 (5)
Mn5^xviii^—Mn1—O1^xv^	130.13 (11)	Ba2^x^—O2—Ba2	93.09 (9)
Mn5^xviii^—Mn1—O1^ix^	49.87 (11)	Ba2^x^—O2—Ba2^xxii^	93.09 (9)
Mn5—Mn1—Mn5^xviii^	180.000	Ba2^x^—O2—O2^xxv^	118.79 (5)
Mn5—Mn1—O1^xi^	130.13 (11)	Ba2^x^—O2—O2^ii^	118.79 (5)
Mn5—Mn1—O1^xvii^	49.87 (11)	Ba2^x^—O2—O2^xxiv^	58.91 (16)
Mn5—Mn1—O1^xvi^	49.87 (11)	Ba2^x^—O2—O2^xi^	58.91 (16)
Mn5—Mn1—O1^x^	130.13 (11)	Ba2^x^—O2—O1^viii^	120.05 (4)
Mn5—Mn1—O1^xv^	49.87 (11)	Ba2^x^—O2—O1^xxi^	120.05 (4)
Mn5—Mn1—O1^ix^	130.13 (11)	Ba2^x^—O2—Nb3^xxiii^	88.65 (19)
O1^xv^—Mn5—O1^xvii^	79.89 (16)	Ba2^x^—O2—Mn5^x^	87.97 (8)
O1^xvi^—Mn5—O1^xv^	79.89 (16)		

Symmetry codes: (i) −*x*+*y*, −*x*, *z*; (ii) −*y*+1, *x*−*y*, *z*; (iii) −*x*+*y*, −*x*+1, *z*; (iv) −*y*, *x*−*y*, *z*; (v) *x*−1, *y*−1, *z*; (vi) *x*−*y*−1/3, −*y*+1/3, −*z*+1/3; (vii) −*x*+2/3, −*y*+1/3, −*z*+1/3; (viii) *y*−1/3, −*x*+*y*+1/3, −*z*+1/3; (ix) *y*−2/3, −*x*+*y*−1/3, −*z*+2/3; (x) −*x*+1/3, −*y*+2/3, −*z*+2/3; (xi) *x*−*y*+1/3, −*y*+2/3, −*z*+2/3; (xii) *x*−2/3, *y*−1/3, *z*−1/3; (xiii) −*x*+*y*+1/3, −*x*+2/3, *z*−1/3; (xiv) −*y*+1/3, *x*−*y*−1/3, *z*−1/3; (xv) *x*−1/3, *y*−2/3, *z*+1/3; (xvi) −*y*+2/3, *x*−*y*+1/3, *z*+1/3; (xvii) −*x*+*y*−1/3, −*x*+1/3, *z*+1/3; (xviii) −*x*, −*y*, −*z*+1; (xix) *x*+1/3, *y*+2/3, *z*−1/3; (xx) −*y*+1, *x*−*y*+1, *z*; (xxi) *x*−*y*+2/3, −*y*+4/3, −*z*+1/3; (xxii) *x*+1, *y*+1, *z*; (xxiii) *x*+2/3, *y*+1/3, *z*+1/3; (xxiv) *y*+1/3, −*x*+*y*+2/3, −*z*+2/3; (xxv) −*x*+*y*+1, −*x*+1, *z*.

### (barium_niobiumV_manganeseIII_oxide_2) Crystal data

**Table:**

BaNb0.5Mn0.5O_3_	*V* = 68.80 (1) Å^3^
*M**_r_* = 259.25	*Z* = 1
Cubic, *P**m*3*m*	*D*_x_ = 6.258 (1) Mg m^−^^3^
*a* = 4.09752 (4) Å	*T* = 300 K

### (barium_niobiumV_manganeseIII_oxide_2) Data collection

**Table:**

SSRL beam line 2-1 diffractometer	

### (barium_niobiumV_manganeseIII_oxide_2) Fractional atomic coordinates and isotropic or equivalent isotropic displacement parameters (Å^2^)

**Table:**

	*x*	*y*	*z*	*B*_iso_*/*B*_eq_	Occ. (<1)
Ba1	0	0	0	0.58 (9)	
Mn1	0.5	0.5	0.5	0.58 (9)	0.5
Nb1	0.5	0.5	0.5	0.58 (9)	0.5
O1	0.5	0.5	0	0.58 (9)	

### (barium_niobiumV_manganeseIII_oxide_2) Geometric parameters (Å, º)

**Table:**

Ba1—O1^i^	2.8974 (1)	Nb1—O1^xiii^	2.0488 (1)
Ba1—O1^ii^	2.8974 (1)	Nb1—O1^viii^	2.0488 (1)
Ba1—O1^iii^	2.8974 (1)	Nb1—O1	2.0488 (1)
Ba1—O1^iv^	2.8974 (1)	Nb1—O1^xiv^	2.0488 (1)
Ba1—O1^v^	2.8974 (1)	Nb1—O1^vii^	2.0488 (1)
Ba1—O1^vi^	2.8974 (1)	O1—Mn1	2.0488 (1)
Ba1—O1^vii^	2.8974 (1)	O1—Nb1	2.0488 (1)
Ba1—O1^viii^	2.8974 (1)	O1—Nb1^xv^	2.0488 (1)
Ba1—O1	2.8974 (1)	O1—Mn1^xv^	2.0488 (1)
Ba1—O1^ix^	2.8974 (1)	O1—Ba1^xvi^	2.8974 (1)
Ba1—O1^x^	2.8974 (1)	O1—Ba1^xvii^	2.8974 (1)
Ba1—O1^xi^	2.8974 (1)	O1—O1^viii^	2.8974 (1)
Mn1—Nb1	0.00000	O1—O1^xviii^	2.8974 (1)
Mn1—O1^xii^	2.0488 (1)	O1—O1^xix^	2.8974 (1)
Mn1—O1^xiii^	2.0488 (1)	O1—O1^vii^	2.8974 (1)
Mn1—O1^viii^	2.0488 (1)	O1—Ba1^xx^	2.8974 (1)
Mn1—O1	2.0488 (1)	O1—O1^xii^	2.8974 (1)
Mn1—O1^xiv^	2.0488 (1)	O1—O1^xiii^	2.8974 (1)
Mn1—O1^vii^	2.0488 (1)	O1—O1^ii^	2.8974 (1)
Nb1—Mn1	0.00000	O1—O1^iv^	2.8974 (1)
Nb1—O1^xii^	2.0488 (1)	O1—Ba1	2.8974 (1)
			
O1^ii^—Ba1—O1^i^	120.000	Ba1^xvi^—O1—Mn1^xv^	90.000
O1^iii^—Ba1—O1^ii^	60.000	Ba1^xvi^—O1—Nb1^xv^	90.000
O1^iii^—Ba1—O1^i^	180.000	Ba1^xvi^—O1—Nb1	90.000
O1^iv^—Ba1—O1^iii^	120.000	Ba1^xvi^—O1—Mn1	90.000
O1^iv^—Ba1—O1^ii^	60.000	Ba1^xvii^—O1—Ba1^xvi^	180.000
O1^iv^—Ba1—O1^i^	60.000	Ba1^xvii^—O1—Mn1^xv^	90.000
O1^v^—Ba1—O1^iv^	180.000	Ba1^xvii^—O1—Nb1^xv^	90.000
O1^v^—Ba1—O1^iii^	60.000	Ba1^xvii^—O1—Nb1	90.000
O1^v^—Ba1—O1^ii^	120.000	Ba1^xvii^—O1—Mn1	90.000
O1^v^—Ba1—O1^i^	120.000	O1^viii^—O1—Ba1^xvii^	120.000
O1^vi^—Ba1—O1^v^	60.000	O1^viii^—O1—Ba1^xvi^	60.000
O1^vi^—Ba1—O1^iv^	120.000	O1^viii^—O1—Mn1^xv^	135.000
O1^vi^—Ba1—O1^iii^	120.000	O1^viii^—O1—Nb1^xv^	135.000
O1^vi^—Ba1—O1^ii^	180.0000 (1)	O1^viii^—O1—Nb1	45.000
O1^vi^—Ba1—O1^i^	60.000	O1^viii^—O1—Mn1	45.000
O1^vii^—Ba1—O1^vi^	60.000	O1^xviii^—O1—O1^viii^	180.000
O1^vii^—Ba1—O1^v^	90.000	O1^xviii^—O1—Ba1^xvii^	60.000
O1^vii^—Ba1—O1^iv^	90.000	O1^xviii^—O1—Ba1^xvi^	120.000
O1^vii^—Ba1—O1^iii^	120.000	O1^xviii^—O1—Mn1^xv^	45.000
O1^vii^—Ba1—O1^ii^	120.000	O1^xviii^—O1—Nb1^xv^	45.000
O1^vii^—Ba1—O1^i^	60.000	O1^xviii^—O1—Nb1	135.000
O1^viii^—Ba1—O1^vii^	60.000	O1^xviii^—O1—Mn1	135.000
O1^viii^—Ba1—O1^vi^	90.000	O1^xix^—O1—O1^xviii^	60.000
O1^viii^—Ba1—O1^v^	60.000	O1^xix^—O1—O1^viii^	120.000
O1^viii^—Ba1—O1^iv^	120.000	O1^xix^—O1—Ba1^xvii^	120.000
O1^viii^—Ba1—O1^iii^	60.000	O1^xix^—O1—Ba1^xvi^	60.000
O1^viii^—Ba1—O1^ii^	90.000	O1^xix^—O1—Mn1^xv^	45.000
O1^viii^—Ba1—O1^i^	120.000	O1^xix^—O1—Nb1^xv^	45.000
O1—Ba1—O1^viii^	60.000	O1^xix^—O1—Nb1	135.000
O1—Ba1—O1^vii^	60.000	O1^xix^—O1—Mn1	135.000
O1—Ba1—O1^vi^	120.000	O1^vii^—O1—O1^xix^	180.0000 (1)
O1—Ba1—O1^v^	120.000	O1^vii^—O1—O1^xviii^	120.000
O1—Ba1—O1^iv^	60.000	O1^vii^—O1—O1^viii^	60.000
O1—Ba1—O1^iii^	90.000	O1^vii^—O1—Ba1^xvii^	60.000
O1—Ba1—O1^ii^	60.000	O1^vii^—O1—Ba1^xvi^	120.000
O1—Ba1—O1^i^	90.000	O1^vii^—O1—Mn1^xv^	135.000
O1^ix^—Ba1—O1	120.000	O1^vii^—O1—Nb1^xv^	135.000
O1^ix^—Ba1—O1^viii^	120.000	O1^vii^—O1—Nb1	45.000
O1^ix^—Ba1—O1^vii^	180.0000 (1)	O1^vii^—O1—Mn1	45.000
O1^ix^—Ba1—O1^vi^	120.000	Ba1^xx^—O1—O1^vii^	120.000
O1^ix^—Ba1—O1^v^	90.000	Ba1^xx^—O1—O1^xix^	60.000
O1^ix^—Ba1—O1^iv^	90.000	Ba1^xx^—O1—O1^xviii^	60.000
O1^ix^—Ba1—O1^iii^	60.000	Ba1^xx^—O1—O1^viii^	120.000
O1^ix^—Ba1—O1^ii^	60.000	Ba1^xx^—O1—Ba1^xvii^	90.000
O1^ix^—Ba1—O1^i^	120.000	Ba1^xx^—O1—Ba1^xvi^	90.000
O1^x^—Ba1—O1^ix^	60.000	Ba1^xx^—O1—Mn1^xv^	90.000
O1^x^—Ba1—O1	120.000	Ba1^xx^—O1—Nb1^xv^	90.000
O1^x^—Ba1—O1^viii^	180.000	Ba1^xx^—O1—Nb1	90.000
O1^x^—Ba1—O1^vii^	120.000	Ba1^xx^—O1—Mn1	90.000
O1^x^—Ba1—O1^vi^	90.000	O1^xii^—O1—Ba1^xx^	60.000
O1^x^—Ba1—O1^v^	120.000	O1^xii^—O1—O1^vii^	60.000
O1^x^—Ba1—O1^iv^	60.000	O1^xii^—O1—O1^xix^	120.000
O1^x^—Ba1—O1^iii^	120.000	O1^xii^—O1—O1^xviii^	90.000
O1^x^—Ba1—O1^ii^	90.000	O1^xii^—O1—O1^viii^	90.000
O1^x^—Ba1—O1^i^	60.000	O1^xii^—O1—Ba1^xvii^	60.000
O1^xi^—Ba1—O1^x^	60.000	O1^xii^—O1—Ba1^xvi^	120.000
O1^xi^—Ba1—O1^ix^	60.000	O1^xii^—O1—Mn1^xv^	135.000
O1^xi^—Ba1—O1	180.000	O1^xii^—O1—Nb1^xv^	135.000
O1^xi^—Ba1—O1^viii^	120.000	O1^xii^—O1—Nb1	45.000
O1^xi^—Ba1—O1^vii^	120.000	O1^xii^—O1—Mn1	45.000
O1^xi^—Ba1—O1^vi^	60.000	O1^xiii^—O1—O1^xii^	60.000
O1^xi^—Ba1—O1^v^	60.000	O1^xiii^—O1—Ba1^xx^	60.000
O1^xi^—Ba1—O1^iv^	120.000	O1^xiii^—O1—O1^vii^	90.000
O1^xi^—Ba1—O1^iii^	90.000	O1^xiii^—O1—O1^xix^	90.000
O1^xi^—Ba1—O1^ii^	120.000	O1^xiii^—O1—O1^xviii^	120.000
O1^xi^—Ba1—O1^i^	90.000	O1^xiii^—O1—O1^viii^	60.000
O1^xii^—Mn1—Nb1		O1^xiii^—O1—Ba1^xvii^	120.000
O1^xiii^—Mn1—O1^xii^	90.000	O1^xiii^—O1—Ba1^xvi^	60.000
O1^xiii^—Mn1—Nb1		O1^xiii^—O1—Mn1^xv^	135.000
O1^viii^—Mn1—O1^xiii^	90.000	O1^xiii^—O1—Nb1^xv^	135.000
O1^viii^—Mn1—O1^xii^	180.000	O1^xiii^—O1—Nb1	45.000
O1^viii^—Mn1—Nb1		O1^xiii^—O1—Mn1	45.000
O1—Mn1—O1^viii^	90.000	O1^ii^—O1—O1^xiii^	120.000
O1—Mn1—O1^xiii^	90.000	O1^ii^—O1—O1^xii^	180.000
O1—Mn1—O1^xii^	90.000	O1^ii^—O1—Ba1^xx^	120.000
O1—Mn1—Nb1		O1^ii^—O1—O1^vii^	120.000
O1^xiv^—Mn1—O1	180.000	O1^ii^—O1—O1^xix^	60.000
O1^xiv^—Mn1—O1^viii^	90.000	O1^ii^—O1—O1^xviii^	90.000
O1^xiv^—Mn1—O1^xiii^	90.000	O1^ii^—O1—O1^viii^	90.000
O1^xiv^—Mn1—O1^xii^	90.000	O1^ii^—O1—Ba1^xvii^	120.000
O1^xiv^—Mn1—Nb1		O1^ii^—O1—Ba1^xvi^	60.000
O1^vii^—Mn1—O1^xiv^	90.000	O1^ii^—O1—Mn1^xv^	45.000
O1^vii^—Mn1—O1	90.000	O1^ii^—O1—Nb1^xv^	45.000
O1^vii^—Mn1—O1^viii^	90.000	O1^ii^—O1—Nb1	135.000
O1^vii^—Mn1—O1^xiii^	180.000	O1^ii^—O1—Mn1	135.000
O1^vii^—Mn1—O1^xii^	90.000	O1^iv^—O1—O1^ii^	60.000
O1^vii^—Mn1—Nb1		O1^iv^—O1—O1^xiii^	180.000
O1^xii^—Nb1—Mn1		O1^iv^—O1—O1^xii^	120.000
O1^xiii^—Nb1—O1^xii^	90.000	O1^iv^—O1—Ba1^xx^	120.000
O1^xiii^—Nb1—Mn1		O1^iv^—O1—O1^vii^	90.000
O1^viii^—Nb1—O1^xiii^	90.000	O1^iv^—O1—O1^xix^	90.000
O1^viii^—Nb1—O1^xii^	180.000	O1^iv^—O1—O1^xviii^	60.000
O1^viii^—Nb1—Mn1		O1^iv^—O1—O1^viii^	120.000
O1—Nb1—O1^viii^	90.000	O1^iv^—O1—Ba1^xvii^	60.000
O1—Nb1—O1^xiii^	90.000	O1^iv^—O1—Ba1^xvi^	120.000
O1—Nb1—O1^xii^	90.000	O1^iv^—O1—Mn1^xv^	45.000
O1—Nb1—Mn1		O1^iv^—O1—Nb1^xv^	45.000
O1^xiv^—Nb1—O1	180.000	O1^iv^—O1—Nb1	135.000
O1^xiv^—Nb1—O1^viii^	90.000	O1^iv^—O1—Mn1	135.000
O1^xiv^—Nb1—O1^xiii^	90.000	Ba1—O1—O1^iv^	60.000
O1^xiv^—Nb1—O1^xii^	90.000	Ba1—O1—O1^ii^	60.000
O1^xiv^—Nb1—Mn1		Ba1—O1—O1^xiii^	120.000
O1^vii^—Nb1—O1^xiv^	90.000	Ba1—O1—O1^xii^	120.000
O1^vii^—Nb1—O1	90.000	Ba1—O1—Ba1^xx^	180.000
O1^vii^—Nb1—O1^viii^	90.000	Ba1—O1—O1^vii^	60.000
O1^vii^—Nb1—O1^xiii^	180.000	Ba1—O1—O1^xix^	120.000
O1^vii^—Nb1—O1^xii^	90.000	Ba1—O1—O1^xviii^	120.000
O1^vii^—Nb1—Mn1		Ba1—O1—O1^viii^	60.000
Nb1—O1—Mn1	0.000	Ba1—O1—Ba1^xvii^	90.000
Nb1^xv^—O1—Nb1	180.000	Ba1—O1—Ba1^xvi^	90.000
Nb1^xv^—O1—Mn1	180.000	Ba1—O1—Mn1^xv^	90.000
Mn1^xv^—O1—Nb1^xv^	0.000	Ba1—O1—Nb1^xv^	90.000
Mn1^xv^—O1—Nb1	180.000	Ba1—O1—Nb1	90.000
Mn1^xv^—O1—Mn1	180.000	Ba1—O1—Mn1	90.000

Symmetry codes: (i) *x*−1, *y*, *z*; (ii) −*x*+1, −*z*, −*y*; (iii) *x*, *y*−1, *z*; (iv) −*z*, −*x*+1, −*y*; (v) −*z*, −*x*, −*y*+1; (vi) −*x*, −*z*, −*y*+1; (vii) −*z*, −*x*+1, −*y*+1; (viii) −*x*+1, −*z*, −*y*+1; (ix) −*z*, −*x*, −*y*; (x) −*x*, −*z*, −*y*; (xi) *x*−1, *y*−1, *z*; (xii) −*x*+1, −*z*+1, −*y*+1; (xiii) −*z*+1, −*x*+1, −*y*+1; (xiv) *x*, *y*, *z*+1; (xv) *x*, *y*, *z*−1; (xvi) *x*+1, *y*, *z*; (xvii) *x*, *y*+1, *z*; (xviii) −*x*+1, −*z*+1, −*y*; (xix) −*z*+1, −*x*+1, −*y*; (xx) *x*+1, *y*+1, *z*.

### (10H-barium_niobium_manganese_oxide) Crystal data

**Table:**

Ba5Nb1.25Mn3.75O_15_	*V* = 668.77 (13) Å^3^
*M**_r_* = 1440.77	*Z* = 1
Hexagonal, *P*6_3_/*m**m**c*	*D*_x_ = 7.155 (1) Mg m^−^^3^
*a* = 5.7331 (4) Å	*T* = 300 K
*c* = 23.494 (3) Å	

### (10H-barium_niobium_manganese_oxide) Data collection

**Table:**

SSRL beam line 2-1 diffractometer	

### (10H-barium_niobium_manganese_oxide) Fractional atomic coordinates and isotropic or equivalent isotropic displacement parameters (Å^2^)

**Table:**

	*x*	*y*	*z*	*B*_iso_*/*B*_eq_	Occ. (<1)
Ba1	0.666667	0.333333	0.25	0.150018	
Ba2	0.666667	0.333333	0.4384	0.150018	
Ba3	0	0	0.3422	0.150018	
Nb4	0	0	0.5	0.157914	
Mn1	0.333333	0.666667	0.3008	0.292140	
Mn2	0.333333	0.666667	0.4046	0.292140	0.875
Nb7	0.333333	0.666667	0.4046	0.292140	0.125
O1	0.1764	0.8075	0.25	0.600072	0.5
O2	0.1773	0.3545	0.4467	0.600072	
O3	0.4817	0.9631	0.351	0.497428	

### (10H-barium_niobium_manganese_oxide) Geometric parameters (Å, º)

**Table:**

Ba1—O1^i^	2.8259 (2)	Mn2—Nb7	0.00000
Ba1—O1^ii^	2.8259 (2)	Mn2—O2	1.8386 (1)
Ba1—O1^iii^	2.8259 (2)	Mn2—O2^xiv^	1.8386 (1)
Ba1—O1^iv^	2.8259 (2)	Mn2—O2^xxv^	1.8386 (1)
Ba1—O1^v^	2.8259 (2)	Mn2—O2^xv^	1.8386 (1)
Ba1—O1^vi^	2.8259 (2)	Mn2—O2^xxiv^	1.8386 (1)
Ba1—O1^vii^	2.9181 (2)	Mn2—O2^xix^	1.8386 (1)
Ba1—O1^viii^	2.9181 (2)	Mn2—O3	1.9370 (1)
Ba1—O1^ix^	2.9181 (2)	Mn2—O3^xxv^	1.9370 (1)
Ba1—O1^x^	2.9181 (2)	Mn2—O3^xiv^	1.9370 (1)
Ba1—O1^xi^	2.9181 (2)	Mn2—O3^xv^	1.9370 (1)
Ba1—O1^xii^	2.9181 (2)	Mn2—O3^xxiv^	1.9370 (1)
Ba1—O3^xiii^	3.0016 (2)	Mn2—O3^xix^	1.9370 (1)
Ba1—O3^xi^	3.0016 (2)	Mn2—Mn1	2.4387 (3)
Ba1—O3^x^	3.0016 (2)	Nb7—Mn2	0.00000
Ba1—O3^xii^	3.0016 (2)	Nb7—O2	1.8386 (1)
Ba1—O3^ix^	3.0016 (2)	Nb7—O2^xiv^	1.8386 (1)
Ba1—O3^viii^	3.0016 (2)	Nb7—O2^xxv^	1.8386 (1)
Ba1—O3^xiv^	3.0016 (2)	Nb7—O2^xv^	1.8386 (1)
Ba1—O3^xv^	3.0016 (2)	Nb7—O2^xxiv^	1.8386 (1)
Ba1—O3^vii^	3.0016 (2)	Nb7—O2^xix^	1.8386 (1)
Ba1—O3^xvi^	3.0016 (2)	Nb7—O3	1.9370 (1)
Ba1—O3^xvii^	3.0016 (2)	Nb7—O3^xxv^	1.9370 (1)
Ba1—O3^xviii^	3.0016 (2)	Nb7—O3^xiv^	1.9370 (1)
Ba2—O3^xvii^	2.7560 (2)	Nb7—O3^xv^	1.9370 (1)
Ba2—O3^xv^	2.7560 (2)	Nb7—O3^xxiv^	1.9370 (1)
Ba2—O3^xviii^	2.7560 (2)	Nb7—O3^xix^	1.9370 (1)
Ba2—O3^xiv^	2.7560 (2)	Nb7—Mn1	2.4387 (3)
Ba2—O3^xi^	2.7560 (2)	O1—O1^xlvii^	0.0923 (1)
Ba2—O3^xiii^	2.7560 (2)	O1—Mn1^viii^	1.9006 (1)
Ba2—O2^xix^	2.8748 (2)	O1—Mn1	1.9006 (1)
Ba2—O2	2.8748 (2)	O1—O1^ix^	2.5146 (2)
Ba2—O2^xx^	2.8748 (2)	O1—O1^viii^	2.5620 (2)
Ba2—O2^xxi^	2.8748 (2)	O1—O1^iii^	2.5620 (2)
Ba2—O2^xxii^	2.8748 (2)	O1—O1^i^	2.6069 (2)
Ba2—O2^xxiii^	2.8748 (2)	O1—O3^xxiv^	2.7729 (3)
Ba2—O2^xxiv^	2.8754 (2)	O1—O3^iii^	2.7729 (3)
Ba2—O2^xxv^	2.8754 (2)	O1—O3^xix^	2.7737 (3)
Ba2—O2^xxvi^	2.8754 (2)	O1—O3^xlvii^	2.7737 (3)
Ba2—O2^vi^	2.8754 (2)	O1—O3^i^	2.8150 (3)
Ba2—O2^xxvii^	2.8754 (2)	O1—O3^xxv^	2.8150 (3)
Ba2—O2^xxviii^	2.8754 (2)	O1—O3^xlviii^	2.8158 (3)
Ba2—O2^xxix^	3.1128 (3)	O1—O3	2.8158 (3)
Ba2—O2^xxx^	3.1128 (3)	O1—Ba1^xlix^	2.8259 (2)
Ba2—O2^xxxi^	3.1128 (3)	O1—Ba3^viii^	2.8371 (2)
Ba2—O2^xxxii^	3.1128 (3)	O1—Ba3^l^	2.8371 (2)
Ba2—O2^xxxiii^	3.1128 (3)	O1—Ba1^l^	2.9181 (2)
Ba2—O2^xxxiv^	3.1128 (3)	O1—O1^li^	3.1263 (2)
Ba3—O1^ix^	2.8371 (2)	O1—O1^lii^	3.1734 (2)
Ba3—O1^iii^	2.8371 (2)	O1—O1^liii^	3.1734 (2)
Ba3—O1^v^	2.8371 (2)	O2—O2^xxv^	0.0006 (1)
Ba3—O1^xi^	2.8371 (2)	O2—Mn2	1.8386 (1)
Ba3—O1^xxxv^	2.8371 (2)	O2—Nb7	1.8386 (1)
Ba3—O1^xxxvi^	2.8371 (2)	O2—Nb4	2.1601 (1)
Ba3—O3^xx^	2.8790 (2)	O2—O2^xix^	2.6843 (2)
Ba3—O3^xiii^	2.8790 (2)	O2—O2^xv^	2.6845 (2)
Ba3—O3^xix^	2.8790 (2)	O2—O2^xxiv^	2.6845 (2)
Ba3—O3^xxxvii^	2.8790 (2)	O2—O2^xiv^	2.6848 (2)
Ba3—O3^xxxviii^	2.8790 (2)	O2—O3^xv^	2.7091 (2)
Ba3—O3^xv^	2.8790 (2)	O2—O3^xix^	2.7094 (2)
Ba3—O3^xxvi^	2.8807 (2)	O2—O3^xiv^	2.7100 (2)
Ba3—O3^xi^	2.8807 (2)	O2—O3^xxiv^	2.7103 (2)
Ba3—O3^xxiv^	2.8807 (2)	O2—Ba2	2.8748 (2)
Ba3—O3^xxxix^	2.8807 (2)	O2—Ba2^xlix^	2.8754 (2)
Ba3—O3^xl^	2.8807 (2)	O2—Ba3	3.0209 (3)
Ba3—O3^xiv^	2.8807 (2)	O2—O2^xxii^	3.0483 (2)
Ba3—O2^xli^	3.0209 (3)	O2—O2^xxvii^	3.0486 (2)
Ba3—O2^xlii^	3.0209 (3)	O2—O2^xli^	3.0486 (2)
Ba3—O2^xxvii^	3.0209 (3)	O2—O2^xlii^	3.0489 (2)
Ba3—O2^xxii^	3.0209 (3)	O2—O2^xxix^	3.0608 (3)
Ba3—O2^xxv^	3.0209 (3)	O2—O2^xliv^	3.0611 (3)
Ba3—O2	3.0209 (3)	O2—O2^xxx^	3.0611 (3)
Nb4—O2^xliii^	2.1601 (1)	O2—O2^xliii^	3.0614 (3)
Nb4—O2	2.1601 (1)	O2—Ba2^xxxiii^	3.1128 (3)
Nb4—O2^xliv^	2.1601 (1)	O3—O3^xxv^	0.0017 (1)
Nb4—O2^xxv^	2.1601 (1)	O3—Mn1	1.8861 (1)
Nb4—O2^xlv^	2.1601 (1)	O3—Nb7	1.9370 (1)
Nb4—O2^xxii^	2.1601 (1)	O3—Mn2	1.9370 (1)
Nb4—O2^xxvii^	2.1601 (1)	O3—O3^xiv^	2.5484 (2)
Nb4—O2^xlvi^	2.1601 (1)	O3—O3^xv^	2.5492 (2)
Nb4—O2^xxix^	2.1601 (1)	O3—O3^xxiv^	2.5492 (2)
Nb4—O2^xxx^	2.1601 (1)	O3—O3^xix^	2.5501 (2)
Nb4—O2^xlii^	2.1601 (1)	O3—O2^xxiv^	2.7091 (2)
Nb4—O2^xli^	2.1601 (1)	O3—O2^xix^	2.7094 (2)
Mn1—O3^xxiv^	1.8861 (1)	O3—O2^xiv^	2.7100 (2)
Mn1—O3^xix^	1.8861 (1)	O3—O2^xv^	2.7103 (2)
Mn1—O3^xiv^	1.8861 (1)	O3—Ba2^l^	2.7560 (2)
Mn1—O3^xv^	1.8861 (1)	O3—O1^viii^	2.7729 (3)
Mn1—O3	1.8861 (1)	O3—O1^xlvii^	2.7737 (3)
Mn1—O3^xxv^	1.8861 (1)	O3—O1^i^	2.8150 (3)
Mn1—O1^viii^	1.9006 (1)	O3—O1	2.8158 (3)
Mn1—O1^i^	1.9006 (1)	O3—Ba3^liv^	2.8790 (2)
Mn1—O1^xlvii^	1.9006 (1)	O3—Ba3^l^	2.8807 (2)
Mn1—O1	1.9006 (1)	O3—Ba1^l^	3.0016 (2)
Mn1—O1^iii^	1.9006 (1)	O3—O3^lv^	3.1830 (2)
Mn1—O1^ix^	1.9006 (1)	O3—O3^lvi^	3.1839 (2)
Mn1—Mn1^viii^	2.3870 (3)	O3—O3^lvii^	3.1839 (2)
Mn1—Nb7	2.4387 (3)	O3—O3^lviii^	3.1848 (2)
Mn1—Mn2	2.4387 (3)		
			
O1^ii^—Ba1—O1^i^	172.835	Nb7—Mn1—O3^xiv^	51.294 (4)
O1^iii^—Ba1—O1^ii^	120.000	Nb7—Mn1—O3^xix^	51.294 (4)
O1^iii^—Ba1—O1^i^	52.835	Nb7—Mn1—O3^xxiv^	51.294 (4)
O1^iv^—Ba1—O1^iii^	172.835	Mn2—Mn1—Nb7	0.000
O1^iv^—Ba1—O1^ii^	67.165	Mn2—Mn1—Mn1^viii^	180.000
O1^iv^—Ba1—O1^i^	120.000	Mn2—Mn1—O1^ix^	128.900 (4)
O1^v^—Ba1—O1^iv^	120.000	Mn2—Mn1—O1^iii^	128.900 (4)
O1^v^—Ba1—O1^iii^	67.165	Mn2—Mn1—O1	128.900 (4)
O1^v^—Ba1—O1^ii^	52.835	Mn2—Mn1—O1^xlvii^	128.900 (4)
O1^v^—Ba1—O1^i^	120.000	Mn2—Mn1—O1^i^	128.900 (4)
O1^vi^—Ba1—O1^v^	172.835	Mn2—Mn1—O1^viii^	128.900 (4)
O1^vi^—Ba1—O1^iv^	52.835	Mn2—Mn1—O3^xxv^	51.294 (4)
O1^vi^—Ba1—O1^iii^	120.000	Mn2—Mn1—O3	51.294 (4)
O1^vi^—Ba1—O1^ii^	120.000	Mn2—Mn1—O3^xv^	51.294 (4)
O1^vi^—Ba1—O1^i^	67.165	Mn2—Mn1—O3^xiv^	51.294 (4)
O1^vii^—Ba1—O1^vi^	119.887	Mn2—Mn1—O3^xix^	51.294 (4)
O1^vii^—Ba1—O1^v^	52.948	Mn2—Mn1—O3^xxiv^	51.294 (4)
O1^vii^—Ba1—O1^iv^	67.052	O2—Mn2—Nb7	
O1^vii^—Ba1—O1^iii^	120.113	O2^xiv^—Mn2—O2	93.792 (5)
O1^vii^—Ba1—O1^ii^	0.1133 (1)	O2^xiv^—Mn2—Nb7	
O1^vii^—Ba1—O1^i^	172.948	O2^xxv^—Mn2—O2^xiv^	93.779 (5)
O1^viii^—Ba1—O1^vii^	173.061	O2^xxv^—Mn2—O2	0.0179 (1)
O1^viii^—Ba1—O1^vi^	67.052	O2^xxv^—Mn2—Nb7	
O1^viii^—Ba1—O1^v^	120.113	O2^xv^—Mn2—O2^xxv^	93.766 (5)
O1^viii^—Ba1—O1^iv^	119.887	O2^xv^—Mn2—O2^xiv^	0.0179 (1)
O1^viii^—Ba1—O1^iii^	52.948	O2^xv^—Mn2—O2	93.779 (5)
O1^viii^—Ba1—O1^ii^	172.948	O2^xv^—Mn2—Nb7	
O1^viii^—Ba1—O1^i^	0.1133 (1)	O2^xxiv^—Mn2—O2^xv^	93.779 (5)
O1^ix^—Ba1—O1^viii^	53.061	O2^xxiv^—Mn2—O2^xxv^	93.792 (5)
O1^ix^—Ba1—O1^vii^	120.000	O2^xxiv^—Mn2—O2^xiv^	93.766 (5)
O1^ix^—Ba1—O1^vi^	120.113	O2^xxiv^—Mn2—O2	93.779 (5)
O1^ix^—Ba1—O1^v^	67.052	O2^xxiv^—Mn2—Nb7	
O1^ix^—Ba1—O1^iv^	172.948	O2^xix^—Mn2—O2^xxiv^	0.0179 (1)
O1^ix^—Ba1—O1^iii^	0.1133 (1)	O2^xix^—Mn2—O2^xv^	93.792 (5)
O1^ix^—Ba1—O1^ii^	119.887	O2^xix^—Mn2—O2^xxv^	93.779 (5)
O1^ix^—Ba1—O1^i^	52.948	O2^xix^—Mn2—O2^xiv^	93.779 (5)
O1^x^—Ba1—O1^ix^	173.061	O2^xix^—Mn2—O2	93.766 (5)
O1^x^—Ba1—O1^viii^	120.000	O2^xix^—Mn2—Nb7	
O1^x^—Ba1—O1^vii^	66.939	O3—Mn2—O2^xix^	91.676 (6)
O1^x^—Ba1—O1^vi^	52.948	O3—Mn2—O2^xxiv^	91.665 (6)
O1^x^—Ba1—O1^v^	119.887	O3—Mn2—O2^xv^	91.713 (6)
O1^x^—Ba1—O1^iv^	0.1133 (1)	O3—Mn2—O2^xxv^	171.9940 (3)
O1^x^—Ba1—O1^iii^	172.948	O3—Mn2—O2^xiv^	91.702 (6)
O1^x^—Ba1—O1^ii^	67.052	O3—Mn2—O2	171.9939 (3)
O1^x^—Ba1—O1^i^	120.113	O3—Mn2—Nb7	
O1^xi^—Ba1—O1^x^	120.000	O3^xxv^—Mn2—O3	0.0509 (1)
O1^xi^—Ba1—O1^ix^	66.939	O3^xxv^—Mn2—O2^xix^	91.713 (6)
O1^xi^—Ba1—O1^viii^	120.000	O3^xxv^—Mn2—O2^xxiv^	91.702 (6)
O1^xi^—Ba1—O1^vii^	53.061	O3^xxv^—Mn2—O2^xv^	91.676 (6)
O1^xi^—Ba1—O1^vi^	172.948	O3^xxv^—Mn2—O2^xxv^	171.9939 (3)
O1^xi^—Ba1—O1^v^	0.1133 (1)	O3^xxv^—Mn2—O2^xiv^	91.665 (6)
O1^xi^—Ba1—O1^iv^	120.113	O3^xxv^—Mn2—O2	171.9940 (3)
O1^xi^—Ba1—O1^iii^	67.052	O3^xxv^—Mn2—Nb7	
O1^xi^—Ba1—O1^ii^	52.948	O3^xiv^—Mn2—O3^xxv^	82.300 (6)
O1^xi^—Ba1—O1^i^	119.887	O3^xiv^—Mn2—O3	82.267 (6)
O1^xii^—Ba1—O1^xi^	173.061	O3^xiv^—Mn2—O2^xix^	91.665 (6)
O1^xii^—Ba1—O1^x^	53.061	O3^xiv^—Mn2—O2^xxiv^	91.676 (6)
O1^xii^—Ba1—O1^ix^	120.000	O3^xiv^—Mn2—O2^xv^	171.9940 (3)
O1^xii^—Ba1—O1^viii^	66.939	O3^xiv^—Mn2—O2^xxv^	91.713 (6)
O1^xii^—Ba1—O1^vii^	120.000	O3^xiv^—Mn2—O2^xiv^	171.9939 (3)
O1^xii^—Ba1—O1^vi^	0.1133 (1)	O3^xiv^—Mn2—O2	91.702 (6)
O1^xii^—Ba1—O1^v^	172.948	O3^xiv^—Mn2—Nb7	
O1^xii^—Ba1—O1^iv^	52.948	O3^xv^—Mn2—O3^xiv^	0.0509 (1)
O1^xii^—Ba1—O1^iii^	119.887	O3^xv^—Mn2—O3^xxv^	82.334 (6)
O1^xii^—Ba1—O1^ii^	120.113	O3^xv^—Mn2—O3	82.300 (6)
O1^xii^—Ba1—O1^i^	67.052	O3^xv^—Mn2—O2^xix^	91.702 (6)
O3^xiii^—Ba1—O1^xii^	120.732 (3)	O3^xv^—Mn2—O2^xxiv^	91.713 (6)
O3^xiii^—Ba1—O1^xi^	56.767 (3)	O3^xv^—Mn2—O2^xv^	171.9939 (3)
O3^xiii^—Ba1—O1^x^	92.1403 (2)	O3^xv^—Mn2—O2^xxv^	91.676 (6)
O3^xiii^—Ba1—O1^ix^	92.1075 (2)	O3^xv^—Mn2—O2^xiv^	171.9940 (3)
O3^xiii^—Ba1—O1^viii^	120.711 (3)	O3^xv^—Mn2—O2	91.665 (6)
O3^xiii^—Ba1—O1^vii^	56.784 (3)	O3^xv^—Mn2—Nb7	
O3^xiii^—Ba1—O1^vi^	120.687 (3)	O3^xxiv^—Mn2—O3^xv^	82.300 (6)
O3^xiii^—Ba1—O1^v^	56.730 (3)	O3^xxiv^—Mn2—O3^xiv^	82.334 (6)
O3^xiii^—Ba1—O1^iv^	92.2096 (2)	O3^xxiv^—Mn2—O3^xxv^	82.267 (6)
O3^xiii^—Ba1—O1^iii^	92.1768 (2)	O3^xxiv^—Mn2—O3	82.300 (6)
O3^xiii^—Ba1—O1^ii^	56.747 (3)	O3^xxiv^—Mn2—O2^xix^	171.9940 (3)
O3^xiii^—Ba1—O1^i^	120.666 (3)	O3^xxiv^—Mn2—O2^xxiv^	171.9939 (3)
O3^xi^—Ba1—O3^xiii^	0.0328 (1)	O3^xxiv^—Mn2—O2^xv^	91.665 (6)
O3^xi^—Ba1—O1^xii^	120.711 (3)	O3^xxiv^—Mn2—O2^xxv^	91.702 (6)
O3^xi^—Ba1—O1^xi^	56.784 (3)	O3^xxiv^—Mn2—O2^xiv^	91.676 (6)
O3^xi^—Ba1—O1^x^	92.1075 (2)	O3^xxiv^—Mn2—O2	91.713 (6)
O3^xi^—Ba1—O1^ix^	92.1403 (2)	O3^xxiv^—Mn2—Nb7	
O3^xi^—Ba1—O1^viii^	120.732 (3)	O3^xix^—Mn2—O3^xxiv^	0.0509 (1)
O3^xi^—Ba1—O1^vii^	56.767 (3)	O3^xix^—Mn2—O3^xv^	82.267 (6)
O3^xi^—Ba1—O1^vi^	120.666 (3)	O3^xix^—Mn2—O3^xiv^	82.300 (6)
O3^xi^—Ba1—O1^v^	56.747 (3)	O3^xix^—Mn2—O3^xxv^	82.300 (6)
O3^xi^—Ba1—O1^iv^	92.1768 (2)	O3^xix^—Mn2—O3	82.334 (6)
O3^xi^—Ba1—O1^iii^	92.2096 (2)	O3^xix^—Mn2—O2^xix^	171.9939 (3)
O3^xi^—Ba1—O1^ii^	56.730 (3)	O3^xix^—Mn2—O2^xxiv^	171.9940 (3)
O3^xi^—Ba1—O1^i^	120.687 (3)	O3^xix^—Mn2—O2^xv^	91.702 (6)
O3^x^—Ba1—O3^xi^	144.339 (3)	O3^xix^—Mn2—O2^xxv^	91.665 (6)
O3^x^—Ba1—O3^xiii^	144.369 (3)	O3^xix^—Mn2—O2^xiv^	91.713 (6)
O3^x^—Ba1—O1^xii^	56.767 (3)	O3^xix^—Mn2—O2	91.676 (6)
O3^x^—Ba1—O1^xi^	120.732 (3)	O3^xix^—Mn2—Nb7	
O3^x^—Ba1—O1^x^	56.784 (3)	Mn1—Mn2—O3^xix^	49.450 (4)
O3^x^—Ba1—O1^ix^	120.711 (3)	Mn1—Mn2—O3^xxiv^	49.450 (4)
O3^x^—Ba1—O1^viii^	92.1075 (2)	Mn1—Mn2—O3^xv^	49.450 (4)
O3^x^—Ba1—O1^vii^	92.1403 (2)	Mn1—Mn2—O3^xiv^	49.450 (4)
O3^x^—Ba1—O1^vi^	56.730 (3)	Mn1—Mn2—O3^xxv^	49.450 (4)
O3^x^—Ba1—O1^v^	120.687 (3)	Mn1—Mn2—O3	49.450 (4)
O3^x^—Ba1—O1^iv^	56.747 (3)	Mn1—Mn2—O2^xix^	122.545 (4)
O3^x^—Ba1—O1^iii^	120.666 (3)	Mn1—Mn2—O2^xxiv^	122.545 (4)
O3^x^—Ba1—O1^ii^	92.2096 (2)	Mn1—Mn2—O2^xv^	122.545 (4)
O3^x^—Ba1—O1^i^	92.1768 (2)	Mn1—Mn2—O2^xxv^	122.545 (4)
O3^xii^—Ba1—O3^x^	0.0328 (1)	Mn1—Mn2—O2^xiv^	122.545 (4)
O3^xii^—Ba1—O3^xi^	144.309 (3)	Mn1—Mn2—O2	122.545 (4)
O3^xii^—Ba1—O3^xiii^	144.339 (3)	Mn1—Mn2—Nb7	
O3^xii^—Ba1—O1^xii^	56.784 (3)	O2—Nb7—Mn2	
O3^xii^—Ba1—O1^xi^	120.711 (3)	O2^xiv^—Nb7—O2	93.792 (5)
O3^xii^—Ba1—O1^x^	56.767 (3)	O2^xiv^—Nb7—Mn2	
O3^xii^—Ba1—O1^ix^	120.732 (3)	O2^xxv^—Nb7—O2^xiv^	93.779 (5)
O3^xii^—Ba1—O1^viii^	92.1403 (2)	O2^xxv^—Nb7—O2	0.0179 (1)
O3^xii^—Ba1—O1^vii^	92.1075 (2)	O2^xxv^—Nb7—Mn2	
O3^xii^—Ba1—O1^vi^	56.747 (3)	O2^xv^—Nb7—O2^xxv^	93.766 (5)
O3^xii^—Ba1—O1^v^	120.666 (3)	O2^xv^—Nb7—O2^xiv^	0.0179 (1)
O3^xii^—Ba1—O1^iv^	56.730 (3)	O2^xv^—Nb7—O2	93.779 (5)
O3^xii^—Ba1—O1^iii^	120.687 (3)	O2^xv^—Nb7—Mn2	
O3^xii^—Ba1—O1^ii^	92.1768 (2)	O2^xxiv^—Nb7—O2^xv^	93.779 (5)
O3^xii^—Ba1—O1^i^	92.2096 (2)	O2^xxiv^—Nb7—O2^xxv^	93.792 (5)
O3^ix^—Ba1—O3^xii^	64.060 (6)	O2^xxiv^—Nb7—O2^xiv^	93.766 (5)
O3^ix^—Ba1—O3^x^	64.041 (6)	O2^xxiv^—Nb7—O2	93.779 (5)
O3^ix^—Ba1—O3^xi^	144.369 (3)	O2^xxiv^—Nb7—Mn2	
O3^ix^—Ba1—O3^xiii^	144.339 (3)	O2^xix^—Nb7—O2^xxiv^	0.0179 (1)
O3^ix^—Ba1—O1^xii^	92.1075 (2)	O2^xix^—Nb7—O2^xv^	93.792 (5)
O3^ix^—Ba1—O1^xi^	92.1403 (2)	O2^xix^—Nb7—O2^xxv^	93.779 (5)
O3^ix^—Ba1—O1^x^	120.711 (3)	O2^xix^—Nb7—O2^xiv^	93.779 (5)
O3^ix^—Ba1—O1^ix^	56.784 (3)	O2^xix^—Nb7—O2	93.766 (5)
O3^ix^—Ba1—O1^viii^	56.767 (3)	O2^xix^—Nb7—Mn2	
O3^ix^—Ba1—O1^vii^	120.732 (3)	O3—Nb7—O2^xix^	91.676 (6)
O3^ix^—Ba1—O1^vi^	92.1768 (2)	O3—Nb7—O2^xxiv^	91.665 (6)
O3^ix^—Ba1—O1^v^	92.2096 (2)	O3—Nb7—O2^xv^	91.713 (6)
O3^ix^—Ba1—O1^iv^	120.666 (3)	O3—Nb7—O2^xxv^	171.9940 (3)
O3^ix^—Ba1—O1^iii^	56.747 (3)	O3—Nb7—O2^xiv^	91.702 (6)
O3^ix^—Ba1—O1^ii^	120.687 (3)	O3—Nb7—O2	171.9939 (3)
O3^ix^—Ba1—O1^i^	56.730 (3)	O3—Nb7—Mn2	
O3^viii^—Ba1—O3^ix^	0.0328 (1)	O3^xxv^—Nb7—O3	0.0509 (1)
O3^viii^—Ba1—O3^xii^	64.079 (6)	O3^xxv^—Nb7—O2^xix^	91.713 (6)
O3^viii^—Ba1—O3^x^	64.060 (6)	O3^xxv^—Nb7—O2^xxiv^	91.702 (6)
O3^viii^—Ba1—O3^xi^	144.339 (3)	O3^xxv^—Nb7—O2^xv^	91.676 (6)
O3^viii^—Ba1—O3^xiii^	144.309 (3)	O3^xxv^—Nb7—O2^xxv^	171.9939 (3)
O3^viii^—Ba1—O1^xii^	92.1403 (2)	O3^xxv^—Nb7—O2^xiv^	91.665 (6)
O3^viii^—Ba1—O1^xi^	92.1075 (2)	O3^xxv^—Nb7—O2	171.9940 (3)
O3^viii^—Ba1—O1^x^	120.732 (3)	O3^xxv^—Nb7—Mn2	
O3^viii^—Ba1—O1^ix^	56.767 (3)	O3^xiv^—Nb7—O3^xxv^	82.300 (6)
O3^viii^—Ba1—O1^viii^	56.784 (3)	O3^xiv^—Nb7—O3	82.267 (6)
O3^viii^—Ba1—O1^vii^	120.711 (3)	O3^xiv^—Nb7—O2^xix^	91.665 (6)
O3^viii^—Ba1—O1^vi^	92.2096 (2)	O3^xiv^—Nb7—O2^xxiv^	91.676 (6)
O3^viii^—Ba1—O1^v^	92.1768 (2)	O3^xiv^—Nb7—O2^xv^	171.9940 (3)
O3^viii^—Ba1—O1^iv^	120.687 (3)	O3^xiv^—Nb7—O2^xxv^	91.713 (6)
O3^viii^—Ba1—O1^iii^	56.730 (3)	O3^xiv^—Nb7—O2^xiv^	171.9939 (3)
O3^viii^—Ba1—O1^ii^	120.666 (3)	O3^xiv^—Nb7—O2	91.702 (6)
O3^viii^—Ba1—O1^i^	56.747 (3)	O3^xiv^—Nb7—Mn2	
O3^xiv^—Ba1—O3^viii^	104.472 (8)	O3^xv^—Nb7—O3^xiv^	0.0509 (1)
O3^xiv^—Ba1—O3^ix^	104.472 (8)	O3^xv^—Nb7—O3^xxv^	82.334 (6)
O3^xiv^—Ba1—O3^xii^	144.339 (3)	O3^xv^—Nb7—O3	82.300 (6)
O3^xiv^—Ba1—O3^x^	144.309 (3)	O3^xv^—Nb7—O2^xix^	91.702 (6)
O3^xiv^—Ba1—O3^xi^	64.079 (6)	O3^xv^—Nb7—O2^xxiv^	91.713 (6)
O3^xiv^—Ba1—O3^xiii^	64.060 (6)	O3^xv^—Nb7—O2^xv^	171.9939 (3)
O3^xiv^—Ba1—O1^xii^	92.1075 (2)	O3^xv^—Nb7—O2^xxv^	91.676 (6)
O3^xiv^—Ba1—O1^xi^	92.1403 (2)	O3^xv^—Nb7—O2^xiv^	171.9940 (3)
O3^xiv^—Ba1—O1^x^	120.711 (3)	O3^xv^—Nb7—O2	91.665 (6)
O3^xiv^—Ba1—O1^ix^	56.784 (3)	O3^xv^—Nb7—Mn2	
O3^xiv^—Ba1—O1^viii^	56.767 (3)	O3^xxiv^—Nb7—O3^xv^	82.300 (6)
O3^xiv^—Ba1—O1^vii^	120.732 (3)	O3^xxiv^—Nb7—O3^xiv^	82.334 (6)
O3^xiv^—Ba1—O1^vi^	92.1768 (2)	O3^xxiv^—Nb7—O3^xxv^	82.267 (6)
O3^xiv^—Ba1—O1^v^	92.2096 (2)	O3^xxiv^—Nb7—O3	82.300 (6)
O3^xiv^—Ba1—O1^iv^	120.666 (3)	O3^xxiv^—Nb7—O2^xix^	171.9940 (3)
O3^xiv^—Ba1—O1^iii^	56.747 (3)	O3^xxiv^—Nb7—O2^xxiv^	171.9939 (3)
O3^xiv^—Ba1—O1^ii^	120.687 (3)	O3^xxiv^—Nb7—O2^xv^	91.665 (6)
O3^xiv^—Ba1—O1^i^	56.730 (3)	O3^xxiv^—Nb7—O2^xxv^	91.702 (6)
O3^xv^—Ba1—O3^xiv^	0.0328 (1)	O3^xxiv^—Nb7—O2^xiv^	91.676 (6)
O3^xv^—Ba1—O3^viii^	104.472 (8)	O3^xxiv^—Nb7—O2	91.713 (6)
O3^xv^—Ba1—O3^ix^	104.472 (8)	O3^xxiv^—Nb7—Mn2	
O3^xv^—Ba1—O3^xii^	144.369 (3)	O3^xix^—Nb7—O3^xxiv^	0.0509 (1)
O3^xv^—Ba1—O3^x^	144.339 (3)	O3^xix^—Nb7—O3^xv^	82.267 (6)
O3^xv^—Ba1—O3^xi^	64.060 (6)	O3^xix^—Nb7—O3^xiv^	82.300 (6)
O3^xv^—Ba1—O3^xiii^	64.041 (6)	O3^xix^—Nb7—O3^xxv^	82.300 (6)
O3^xv^—Ba1—O1^xii^	92.1403 (2)	O3^xix^—Nb7—O3	82.334 (6)
O3^xv^—Ba1—O1^xi^	92.1075 (2)	O3^xix^—Nb7—O2^xix^	171.9939 (3)
O3^xv^—Ba1—O1^x^	120.732 (3)	O3^xix^—Nb7—O2^xxiv^	171.9940 (3)
O3^xv^—Ba1—O1^ix^	56.767 (3)	O3^xix^—Nb7—O2^xv^	91.702 (6)
O3^xv^—Ba1—O1^viii^	56.784 (3)	O3^xix^—Nb7—O2^xxv^	91.665 (6)
O3^xv^—Ba1—O1^vii^	120.711 (3)	O3^xix^—Nb7—O2^xiv^	91.713 (6)
O3^xv^—Ba1—O1^vi^	92.2096 (2)	O3^xix^—Nb7—O2	91.676 (6)
O3^xv^—Ba1—O1^v^	92.1768 (2)	O3^xix^—Nb7—Mn2	
O3^xv^—Ba1—O1^iv^	120.687 (3)	Mn1—Nb7—O3^xix^	49.450 (4)
O3^xv^—Ba1—O1^iii^	56.730 (3)	Mn1—Nb7—O3^xxiv^	49.450 (4)
O3^xv^—Ba1—O1^ii^	120.666 (3)	Mn1—Nb7—O3^xv^	49.450 (4)
O3^xv^—Ba1—O1^i^	56.747 (3)	Mn1—Nb7—O3^xiv^	49.450 (4)
O3^vii^—Ba1—O3^xv^	144.309 (3)	Mn1—Nb7—O3^xxv^	49.450 (4)
O3^vii^—Ba1—O3^xiv^	144.339 (3)	Mn1—Nb7—O3	49.450 (4)
O3^vii^—Ba1—O3^viii^	64.041 (6)	Mn1—Nb7—O2^xix^	122.545 (4)
O3^vii^—Ba1—O3^ix^	64.060 (6)	Mn1—Nb7—O2^xxiv^	122.545 (4)
O3^vii^—Ba1—O3^xii^	64.060 (6)	Mn1—Nb7—O2^xv^	122.545 (4)
O3^vii^—Ba1—O3^x^	64.079 (6)	Mn1—Nb7—O2^xxv^	122.545 (4)
O3^vii^—Ba1—O3^xi^	104.472 (8)	Mn1—Nb7—O2^xiv^	122.545 (4)
O3^vii^—Ba1—O3^xiii^	104.472 (8)	Mn1—Nb7—O2	122.545 (4)
O3^vii^—Ba1—O1^xii^	120.732 (3)	Mn1—Nb7—Mn2	
O3^vii^—Ba1—O1^xi^	56.767 (3)	Mn1^viii^—O1—O1^xlvii^	88.6086 (1)
O3^vii^—Ba1—O1^x^	92.1403 (2)	Mn1—O1—Mn1^viii^	77.799 (8)
O3^vii^—Ba1—O1^ix^	92.1075 (2)	Mn1—O1—O1^xlvii^	88.6086 (1)
O3^vii^—Ba1—O1^viii^	120.711 (3)	O1^ix^—O1—Mn1	48.585 (3)
O3^vii^—Ba1—O1^vii^	56.784 (3)	O1^ix^—O1—Mn1^viii^	48.585 (3)
O3^vii^—Ba1—O1^vi^	120.687 (3)	O1^ix^—O1—O1^xlvii^	120.000
O3^vii^—Ba1—O1^v^	56.730 (3)	O1^viii^—O1—O1^ix^	61.788
O3^vii^—Ba1—O1^iv^	92.2096 (2)	O1^viii^—O1—Mn1	47.625 (3)
O3^vii^—Ba1—O1^iii^	92.1768 (2)	O1^viii^—O1—Mn1^viii^	47.625 (3)
O3^vii^—Ba1—O1^ii^	56.747 (3)	O1^viii^—O1—O1^xlvii^	58.212
O3^vii^—Ba1—O1^i^	120.666 (3)	O1^iii^—O1—O1^viii^	60.000
O3^xvi^—Ba1—O3^vii^	0.0328 (1)	O1^iii^—O1—O1^ix^	1.788
O3^xvi^—Ba1—O3^xv^	144.339 (3)	O1^iii^—O1—Mn1	47.625 (3)
O3^xvi^—Ba1—O3^xiv^	144.369 (3)	O1^iii^—O1—Mn1^viii^	47.625 (3)
O3^xvi^—Ba1—O3^viii^	64.060 (6)	O1^iii^—O1—O1^xlvii^	118.212
O3^xvi^—Ba1—O3^ix^	64.079 (6)	O1^i^—O1—O1^iii^	58.212
O3^xvi^—Ba1—O3^xii^	64.041 (6)	O1^i^—O1—O1^viii^	1.788
O3^xvi^—Ba1—O3^x^	64.060 (6)	O1^i^—O1—O1^ix^	60.000
O3^xvi^—Ba1—O3^xi^	104.472 (8)	O1^i^—O1—Mn1	46.702 (3)
O3^xvi^—Ba1—O3^xiii^	104.472 (8)	O1^i^—O1—Mn1^viii^	46.702 (3)
O3^xvi^—Ba1—O1^xii^	120.711 (3)	O1^i^—O1—O1^xlvii^	60.000
O3^xvi^—Ba1—O1^xi^	56.784 (3)	O3^xxiv^—O1—O1^i^	89.4136 (1)
O3^xvi^—Ba1—O1^x^	92.1075 (2)	O3^xxiv^—O1—O1^iii^	63.571 (3)
O3^xvi^—Ba1—O1^ix^	92.1403 (2)	O3^xxiv^—O1—O1^viii^	90.3387 (1)
O3^xvi^—Ba1—O1^viii^	120.732 (3)	O3^xxiv^—O1—O1^ix^	63.057 (3)
O3^xvi^—Ba1—O1^vii^	56.767 (3)	O3^xxiv^—O1—Mn1	42.718 (3)
O3^xvi^—Ba1—O1^vi^	120.666 (3)	O3^xxiv^—O1—Mn1^viii^	109.879 (6)
O3^xvi^—Ba1—O1^v^	56.747 (3)	O3^xxiv^—O1—O1^xlvii^	116.287 (3)
O3^xvi^—Ba1—O1^iv^	92.1768 (2)	O3^iii^—O1—O3^xxiv^	117.685 (7)
O3^xvi^—Ba1—O1^iii^	92.2096 (2)	O3^iii^—O1—O1^i^	89.4136 (1)
O3^xvi^—Ba1—O1^ii^	56.730 (3)	O3^iii^—O1—O1^iii^	63.571 (3)
O3^xvi^—Ba1—O1^i^	120.687 (3)	O3^iii^—O1—O1^viii^	90.3387 (1)
O3^xvii^—Ba1—O3^xvi^	144.339 (3)	O3^iii^—O1—O1^ix^	63.057 (3)
O3^xvii^—Ba1—O3^vii^	144.369 (3)	O3^iii^—O1—Mn1	109.879 (6)
O3^xvii^—Ba1—O3^xv^	64.060 (6)	O3^iii^—O1—Mn1^viii^	42.718 (3)
O3^xvii^—Ba1—O3^xiv^	64.041 (6)	O3^iii^—O1—O1^xlvii^	116.287 (3)
O3^xvii^—Ba1—O3^viii^	144.339 (3)	O3^xix^—O1—O3^iii^	117.658 (7)
O3^xvii^—Ba1—O3^ix^	144.309 (3)	O3^xix^—O1—O3^xxiv^	0.0317 (1)
O3^xvii^—Ba1—O3^xii^	104.472 (8)	O3^xix^—O1—O1^i^	89.3960 (1)
O3^xvii^—Ba1—O3^x^	104.472 (8)	O3^xix^—O1—O1^iii^	63.539 (3)
O3^xvii^—Ba1—O3^xi^	64.060 (6)	O3^xix^—O1—O1^viii^	90.3218 (1)
O3^xvii^—Ba1—O3^xiii^	64.079 (6)	O3^xix^—O1—O1^ix^	63.025 (3)
O3^xvii^—Ba1—O1^xii^	56.767 (3)	O3^xix^—O1—Mn1	42.700 (3)
O3^xvii^—Ba1—O1^xi^	120.732 (3)	O3^xix^—O1—Mn1^viii^	109.848 (6)
O3^xvii^—Ba1—O1^x^	56.784 (3)	O3^xix^—O1—O1^xlvii^	116.299 (3)
O3^xvii^—Ba1—O1^ix^	120.711 (3)	O3^xlvii^—O1—O3^xix^	117.632 (7)
O3^xvii^—Ba1—O1^viii^	92.1075 (2)	O3^xlvii^—O1—O3^iii^	0.0317 (1)
O3^xvii^—Ba1—O1^vii^	92.1403 (2)	O3^xlvii^—O1—O3^xxiv^	117.658 (7)
O3^xvii^—Ba1—O1^vi^	56.730 (3)	O3^xlvii^—O1—O1^i^	89.3960 (1)
O3^xvii^—Ba1—O1^v^	120.687 (3)	O3^xlvii^—O1—O1^iii^	63.539 (3)
O3^xvii^—Ba1—O1^iv^	56.747 (3)	O3^xlvii^—O1—O1^viii^	90.3218 (1)
O3^xvii^—Ba1—O1^iii^	120.666 (3)	O3^xlvii^—O1—O1^ix^	63.025 (3)
O3^xvii^—Ba1—O1^ii^	92.2096 (2)	O3^xlvii^—O1—Mn1	109.848 (6)
O3^xvii^—Ba1—O1^i^	92.1768 (2)	O3^xlvii^—O1—Mn1^viii^	42.700 (3)
O3^xviii^—Ba1—O3^xvii^	0.0328 (1)	O3^xlvii^—O1—O1^xlvii^	116.299 (3)
O3^xviii^—Ba1—O3^xvi^	144.309 (3)	O3^i^—O1—O3^xlvii^	54.271 (6)
O3^xviii^—Ba1—O3^vii^	144.339 (3)	O3^i^—O1—O3^xix^	149.132 (3)
O3^xviii^—Ba1—O3^xv^	64.079 (6)	O3^i^—O1—O3^iii^	54.259 (6)
O3^xviii^—Ba1—O3^xiv^	64.060 (6)	O3^i^—O1—O3^xxiv^	149.158 (3)
O3^xviii^—Ba1—O3^viii^	144.369 (3)	O3^i^—O1—O1^i^	62.437 (3)
O3^xviii^—Ba1—O3^ix^	144.339 (3)	O3^i^—O1—O1^iii^	89.3998 (1)
O3^xviii^—Ba1—O3^xii^	104.472 (8)	O3^i^—O1—O1^viii^	61.897 (3)
O3^xviii^—Ba1—O3^x^	104.472 (8)	O3^i^—O1—O1^ix^	90.3617 (1)
O3^xviii^—Ba1—O3^xi^	64.041 (6)	O3^i^—O1—Mn1	108.238 (6)
O3^xviii^—Ba1—O3^xiii^	64.060 (6)	O3^i^—O1—Mn1^viii^	41.781 (3)
O3^xviii^—Ba1—O1^xii^	56.784 (3)	O3^i^—O1—O1^xlvii^	62.028 (3)
O3^xviii^—Ba1—O1^xi^	120.711 (3)	O3^xxv^—O1—O3^i^	114.906 (7)
O3^xviii^—Ba1—O1^x^	56.767 (3)	O3^xxv^—O1—O3^xlvii^	149.132 (3)
O3^xviii^—Ba1—O1^ix^	120.732 (3)	O3^xxv^—O1—O3^xix^	54.271 (6)
O3^xviii^—Ba1—O1^viii^	92.1403 (2)	O3^xxv^—O1—O3^iii^	149.158 (3)
O3^xviii^—Ba1—O1^vii^	92.1075 (2)	O3^xxv^—O1—O3^xxiv^	54.259 (6)
O3^xviii^—Ba1—O1^vi^	56.747 (3)	O3^xxv^—O1—O1^i^	62.437 (3)
O3^xviii^—Ba1—O1^v^	120.666 (3)	O3^xxv^—O1—O1^iii^	89.3998 (1)
O3^xviii^—Ba1—O1^iv^	56.730 (3)	O3^xxv^—O1—O1^viii^	61.897 (3)
O3^xviii^—Ba1—O1^iii^	120.687 (3)	O3^xxv^—O1—O1^ix^	90.3617 (1)
O3^xviii^—Ba1—O1^ii^	92.1768 (2)	O3^xxv^—O1—Mn1	41.781 (3)
O3^xviii^—Ba1—O1^i^	92.2096 (2)	O3^xxv^—O1—Mn1^viii^	108.238 (6)
O3^xv^—Ba2—O3^xvii^	70.568 (6)	O3^xxv^—O1—O1^xlvii^	62.028 (3)
O3^xviii^—Ba2—O3^xv^	70.590 (6)	O3^xlviii^—O1—O3^xxv^	114.881 (7)
O3^xviii^—Ba2—O3^xvii^	0.0358 (1)	O3^xlviii^—O1—O3^i^	0.0310 (1)
O3^xiv^—Ba2—O3^xviii^	70.568 (6)	O3^xlviii^—O1—O3^xlvii^	54.282 (6)
O3^xiv^—Ba2—O3^xv^	0.0358 (1)	O3^xlviii^—O1—O3^xix^	149.104 (3)
O3^xiv^—Ba2—O3^xvii^	70.547 (6)	O3^xlviii^—O1—O3^iii^	54.270 (6)
O3^xi^—Ba2—O3^xiv^	70.590 (6)	O3^xlviii^—O1—O3^xxiv^	149.130 (3)
O3^xi^—Ba2—O3^xviii^	70.547 (6)	O3^xlviii^—O1—O1^i^	62.406 (3)
O3^xi^—Ba2—O3^xv^	70.568 (6)	O3^xlviii^—O1—O1^iii^	89.3816 (1)
O3^xi^—Ba2—O3^xvii^	70.568 (6)	O3^xlviii^—O1—O1^viii^	61.866 (3)
O3^xiii^—Ba2—O3^xi^	0.0358 (1)	O3^xlviii^—O1—O1^ix^	90.3441 (1)
O3^xiii^—Ba2—O3^xiv^	70.568 (6)	O3^xlviii^—O1—Mn1	108.208 (6)
O3^xiii^—Ba2—O3^xviii^	70.568 (6)	O3^xlviii^—O1—Mn1^viii^	41.763 (3)
O3^xiii^—Ba2—O3^xv^	70.547 (6)	O3^xlviii^—O1—O1^xlvii^	62.017 (3)
O3^xiii^—Ba2—O3^xvii^	70.590 (6)	O3—O1—O3^xlviii^	114.855 (7)
O2^xix^—Ba2—O3^xiii^	127.886 (3)	O3—O1—O3^xxv^	0.0310 (1)
O2^xix^—Ba2—O3^xi^	127.910 (3)	O3—O1—O3^i^	114.881 (7)
O2^xix^—Ba2—O3^xiv^	57.472 (4)	O3—O1—O3^xlvii^	149.104 (3)
O2^xix^—Ba2—O3^xviii^	94.3146 (5)	O3—O1—O3^xix^	54.282 (6)
O2^xix^—Ba2—O3^xv^	57.492 (4)	O3—O1—O3^iii^	149.130 (3)
O2^xix^—Ba2—O3^xvii^	94.2788 (5)	O3—O1—O3^xxiv^	54.270 (6)
O2—Ba2—O2^xix^	55.6606 (1)	O3—O1—O1^i^	62.406 (3)
O2—Ba2—O3^xiii^	94.2788 (5)	O3—O1—O1^iii^	89.3816 (1)
O2—Ba2—O3^xi^	94.3146 (5)	O3—O1—O1^viii^	61.866 (3)
O2—Ba2—O3^xiv^	57.492 (4)	O3—O1—O1^ix^	90.3441 (1)
O2—Ba2—O3^xviii^	127.910 (3)	O3—O1—Mn1	41.763 (3)
O2—Ba2—O3^xv^	57.472 (4)	O3—O1—Mn1^viii^	108.208 (6)
O2—Ba2—O3^xvii^	127.886 (3)	O3—O1—O1^xlvii^	62.017 (3)
O2^xx^—Ba2—O2	119.5444 (1)	Ba1^xlix^—O1—O3	118.999 (3)
O2^xx^—Ba2—O2^xix^	171.1615 (10)	Ba1^xlix^—O1—O3^xlviii^	118.999 (3)
O2^xx^—Ba2—O3^xiii^	57.492 (4)	Ba1^xlix^—O1—O3^xxv^	118.985 (3)
O2^xx^—Ba2—O3^xi^	57.472 (4)	Ba1^xlix^—O1—O3^i^	118.985 (3)
O2^xx^—Ba2—O3^xiv^	127.910 (3)	Ba1^xlix^—O1—O3^xlvii^	64.821 (3)
O2^xx^—Ba2—O3^xviii^	94.2788 (5)	Ba1^xlix^—O1—O3^xix^	64.821 (3)
O2^xx^—Ba2—O3^xv^	127.886 (3)	Ba1^xlix^—O1—O3^iii^	64.831 (3)
O2^xx^—Ba2—O3^xvii^	94.3146 (5)	Ba1^xlix^—O1—O3^xxiv^	64.831 (3)
O2^xxi^—Ba2—O2^xx^	64.0342 (1)	Ba1^xlix^—O1—O1^i^	123.583
O2^xxi^—Ba2—O2	171.1615 (10)	Ba1^xlix^—O1—O1^iii^	65.371
O2^xxi^—Ba2—O2^xix^	119.5444 (1)	Ba1^xlix^—O1—O1^viii^	125.371
O2^xxi^—Ba2—O3^xiii^	94.3146 (5)	Ba1^xlix^—O1—O1^ix^	63.583
O2^xxi^—Ba2—O3^xi^	94.2788 (5)	Ba1^xlix^—O1—Mn1	94.1773 (2)
O2^xxi^—Ba2—O3^xiv^	127.886 (3)	Ba1^xlix^—O1—Mn1^viii^	94.1773 (2)
O2^xxi^—Ba2—O3^xviii^	57.472 (4)	Ba1^xlix^—O1—O1^xlvii^	176.417
O2^xxi^—Ba2—O3^xv^	127.910 (3)	Ba3^viii^—O1—Ba1^xlix^	88.6187 (1)
O2^xxi^—Ba2—O3^xvii^	57.492 (4)	Ba3^viii^—O1—O3	143.726 (3)
O2^xxii^—Ba2—O2^xxi^	119.5444 (1)	Ba3^viii^—O1—O3^xlviii^	61.273 (6)
O2^xxii^—Ba2—O2^xx^	55.6606 (1)	Ba3^viii^—O1—O3^xxv^	143.716 (3)
O2^xxii^—Ba2—O2	64.0342 (1)	Ba3^viii^—O1—O3^i^	61.242 (6)
O2^xxii^—Ba2—O2^xix^	119.5444 (1)	Ba3^viii^—O1—O3^xlvii^	61.772 (6)
O2^xxii^—Ba2—O3^xiii^	57.472 (4)	Ba3^viii^—O1—O3^xix^	146.449 (3)
O2^xxii^—Ba2—O3^xi^	57.492 (4)	Ba3^viii^—O1—O3^iii^	61.740 (6)
O2^xxii^—Ba2—O3^xiv^	94.3146 (5)	Ba3^viii^—O1—O3^xxiv^	146.437 (3)
O2^xxii^—Ba2—O3^xviii^	127.886 (3)	Ba3^viii^—O1—O1^i^	123.433 (3)
O2^xxii^—Ba2—O3^xv^	94.2788 (5)	Ba3^viii^—O1—O1^iii^	125.211 (3)
O2^xxii^—Ba2—O3^xvii^	127.910 (3)	Ba3^viii^—O1—O1^viii^	122.696 (3)
O2^xxiii^—Ba2—O2^xxii^	171.1615 (10)	Ba3^viii^—O1—O1^ix^	124.557 (3)
O2^xxiii^—Ba2—O2^xxi^	55.6606 (1)	Ba3^viii^—O1—Mn1	168.8848 (1)
O2^xxiii^—Ba2—O2^xx^	119.5444 (1)	Ba3^viii^—O1—Mn1^viii^	91.280 (8)
O2^xxiii^—Ba2—O2	119.5444 (1)	Ba3^viii^—O1—O1^xlvii^	89.0679 (1)
O2^xxiii^—Ba2—O2^xix^	64.0342 (1)	Ba3^l^—O1—Ba3^viii^	99.549 (8)
O2^xxiii^—Ba2—O3^xiii^	127.910 (3)	Ba3^l^—O1—Ba1^xlix^	88.6187 (1)
O2^xxiii^—Ba2—O3^xi^	127.886 (3)	Ba3^l^—O1—O3	61.273 (6)
O2^xxiii^—Ba2—O3^xiv^	94.2788 (5)	Ba3^l^—O1—O3^xlviii^	143.726 (3)
O2^xxiii^—Ba2—O3^xviii^	57.492 (4)	Ba3^l^—O1—O3^xxv^	61.242 (6)
O2^xxiii^—Ba2—O3^xv^	94.3146 (5)	Ba3^l^—O1—O3^i^	143.716 (3)
O2^xxiii^—Ba2—O3^xvii^	57.472 (4)	Ba3^l^—O1—O3^xlvii^	146.449 (3)
O2^xxiv^—Ba2—O2^xxiii^	64.0338 (1)	Ba3^l^—O1—O3^xix^	61.772 (6)
O2^xxiv^—Ba2—O2^xxii^	119.5450 (1)	Ba3^l^—O1—O3^iii^	146.437 (3)
O2^xxiv^—Ba2—O2^xxi^	119.5441 (1)	Ba3^l^—O1—O3^xxiv^	61.740 (6)
O2^xxiv^—Ba2—O2^xx^	171.1624 (10)	Ba3^l^—O1—O1^i^	123.433 (3)
O2^xxiv^—Ba2—O2	55.6611 (1)	Ba3^l^—O1—O1^iii^	125.211 (3)
O2^xxiv^—Ba2—O2^xix^	0.0009 (1)	Ba3^l^—O1—O1^viii^	122.696 (3)
O2^xxiv^—Ba2—O3^xiii^	127.886 (3)	Ba3^l^—O1—O1^ix^	124.557 (3)
O2^xxiv^—Ba2—O3^xi^	127.910 (3)	Ba3^l^—O1—Mn1	91.280 (8)
O2^xxiv^—Ba2—O3^xiv^	57.471 (4)	Ba3^l^—O1—Mn1^viii^	168.8848 (1)
O2^xxiv^—Ba2—O3^xviii^	94.3137 (5)	Ba3^l^—O1—O1^xlvii^	89.0679 (1)
O2^xxiv^—Ba2—O3^xv^	57.491 (4)	Ba1^l^—O1—Ba3^l^	86.8296 (3)
O2^xxiv^—Ba2—O3^xvii^	94.2779 (5)	Ba1^l^—O1—Ba3^viii^	86.8296 (3)
O2^xxv^—Ba2—O2^xxiv^	55.6615 (1)	Ba1^l^—O1—Ba1^xlix^	172.948
O2^xxv^—Ba2—O2^xxiii^	119.5450 (1)	Ba1^l^—O1—O3	63.104 (3)
O2^xxv^—Ba2—O2^xxii^	64.0338 (1)	Ba1^l^—O1—O3^xlviii^	63.104 (3)
O2^xxv^—Ba2—O2^xxi^	171.1624 (10)	Ba1^l^—O1—O3^xxv^	63.113 (3)
O2^xxv^—Ba2—O2^xx^	119.5441 (1)	Ba1^l^—O1—O3^i^	63.113 (3)
O2^xxv^—Ba2—O2	0.0009 (1)	Ba1^l^—O1—O3^xlvii^	117.288 (3)
O2^xxv^—Ba2—O2^xix^	55.6611 (1)	Ba1^l^—O1—O3^xix^	117.288 (3)
O2^xxv^—Ba2—O3^xiii^	94.2779 (5)	Ba1^l^—O1—O3^iii^	117.274 (3)
O2^xxv^—Ba2—O3^xi^	94.3137 (5)	Ba1^l^—O1—O3^xxiv^	117.274 (3)
O2^xxv^—Ba2—O3^xiv^	57.491 (4)	Ba1^l^—O1—O1^i^	63.469
O2^xxv^—Ba2—O3^xviii^	127.910 (3)	Ba1^l^—O1—O1^iii^	121.681
O2^xxv^—Ba2—O3^xv^	57.471 (4)	Ba1^l^—O1—O1^viii^	61.681
O2^xxv^—Ba2—O3^xvii^	127.886 (3)	Ba1^l^—O1—O1^ix^	123.469
O2^xxvi^—Ba2—O2^xxv^	119.5446 (1)	Ba1^l^—O1—Mn1	91.3085 (1)
O2^xxvi^—Ba2—O2^xxiv^	171.1632 (10)	Ba1^l^—O1—Mn1^viii^	91.3085 (1)
O2^xxvi^—Ba2—O2^xxiii^	119.5441 (1)	Ba1^l^—O1—O1^xlvii^	3.469
O2^xxvi^—Ba2—O2^xxii^	55.6611 (1)	O1^li^—O1—Ba1^l^	116.531
O2^xxvi^—Ba2—O2^xxi^	64.0338 (1)	O1^li^—O1—Ba3^l^	56.567 (3)
O2^xxvi^—Ba2—O2^xx^	0.0009 (1)	O1^li^—O1—Ba3^viii^	56.567 (3)
O2^xxvi^—Ba2—O2	119.5450 (1)	O1^li^—O1—Ba1^xlix^	56.417
O2^xxvi^—Ba2—O2^xix^	171.1624 (10)	O1^li^—O1—O3	117.594 (3)
O2^xxvi^—Ba2—O3^xiii^	57.491 (4)	O1^li^—O1—O3^xlviii^	117.594 (3)
O2^xxvi^—Ba2—O3^xi^	57.471 (4)	O1^li^—O1—O3^xxv^	117.563 (3)
O2^xxvi^—Ba2—O3^xiv^	127.910 (3)	O1^li^—O1—O3^i^	117.563 (3)
O2^xxvi^—Ba2—O3^xviii^	94.2779 (5)	O1^li^—O1—O3^xlvii^	90.6040 (1)
O2^xxvi^—Ba2—O3^xv^	127.886 (3)	O1^li^—O1—O3^xix^	90.6040 (1)
O2^xxvi^—Ba2—O3^xvii^	94.3137 (5)	O1^li^—O1—O3^iii^	90.5864 (1)
O2^vi^—Ba2—O2^xxvi^	64.0334 (1)	O1^li^—O1—O3^xxiv^	90.5864 (1)
O2^vi^—Ba2—O2^xxv^	171.1632 (10)	O1^li^—O1—O1^i^	180.000
O2^vi^—Ba2—O2^xxiv^	119.5446 (1)	O1^li^—O1—O1^iii^	121.788
O2^vi^—Ba2—O2^xxiii^	55.6611 (1)	O1^li^—O1—O1^viii^	178.212
O2^vi^—Ba2—O2^xxii^	119.5441 (1)	O1^li^—O1—O1^ix^	120.000
O2^vi^—Ba2—O2^xxi^	0.0009 (1)	O1^li^—O1—Mn1	133.298 (3)
O2^vi^—Ba2—O2^xx^	64.0338 (1)	O1^li^—O1—Mn1^viii^	133.298 (3)
O2^vi^—Ba2—O2	171.1624 (10)	O1^li^—O1—O1^xlvii^	120.000
O2^vi^—Ba2—O2^xix^	119.5450 (1)	O1^lii^—O1—O1^li^	1.443
O2^vi^—Ba2—O3^xiii^	94.3137 (5)	O1^lii^—O1—Ba1^l^	115.087
O2^vi^—Ba2—O3^xi^	94.2779 (5)	O1^lii^—O1—Ba3^l^	55.994 (3)
O2^vi^—Ba2—O3^xiv^	127.886 (3)	O1^lii^—O1—Ba3^viii^	55.994 (3)
O2^vi^—Ba2—O3^xviii^	57.471 (4)	O1^lii^—O1—Ba1^xlix^	57.861
O2^vi^—Ba2—O3^xv^	127.910 (3)	O1^lii^—O1—O3	117.139 (3)
O2^vi^—Ba2—O3^xvii^	57.491 (4)	O1^lii^—O1—O3^xlviii^	117.139 (3)
O2^xxvii^—Ba2—O2^vi^	119.5446 (1)	O1^lii^—O1—O3^xxv^	117.108 (3)
O2^xxvii^—Ba2—O2^xxvi^	55.6615 (1)	O1^lii^—O1—O3^i^	117.108 (3)
O2^xxvii^—Ba2—O2^xxv^	64.0334 (1)	O1^lii^—O1—O3^xlvii^	91.3511 (1)
O2^xxvii^—Ba2—O2^xxiv^	119.5446 (1)	O1^lii^—O1—O3^xix^	91.3511 (1)
O2^xxvii^—Ba2—O2^xxiii^	171.1624 (10)	O1^lii^—O1—O3^iii^	91.3329 (1)
O2^xxvii^—Ba2—O2^xxii^	0.0009 (1)	O1^lii^—O1—O3^xxiv^	91.3329 (1)
O2^xxvii^—Ba2—O2^xxi^	119.5450 (1)	O1^lii^—O1—O1^i^	178.557
O2^xxvii^—Ba2—O2^xx^	55.6611 (1)	O1^lii^—O1—O1^iii^	123.231
O2^xxvii^—Ba2—O2	64.0338 (1)	O1^lii^—O1—O1^viii^	176.769
O2^xxvii^—Ba2—O2^xix^	119.5441 (1)	O1^lii^—O1—O1^ix^	121.443
O2^xxvii^—Ba2—O3^xiii^	57.471 (4)	O1^lii^—O1—Mn1	134.015 (3)
O2^xxvii^—Ba2—O3^xi^	57.491 (4)	O1^lii^—O1—Mn1^viii^	134.015 (3)
O2^xxvii^—Ba2—O3^xiv^	94.3137 (5)	O1^lii^—O1—O1^xlvii^	118.557
O2^xxvii^—Ba2—O3^xviii^	127.886 (3)	O1^liii^—O1—O1^lii^	60.000
O2^xxvii^—Ba2—O3^xv^	94.2779 (5)	O1^liii^—O1—O1^li^	61.443
O2^xxvii^—Ba2—O3^xvii^	127.910 (3)	O1^liii^—O1—Ba1^l^	55.087
O2^xxviii^—Ba2—O2^xxvii^	171.1632 (10)	O1^liii^—O1—Ba3^l^	55.994 (3)
O2^xxviii^—Ba2—O2^vi^	55.6615 (1)	O1^liii^—O1—Ba3^viii^	55.994 (3)
O2^xxviii^—Ba2—O2^xxvi^	119.5446 (1)	O1^liii^—O1—Ba1^xlix^	117.861
O2^xxviii^—Ba2—O2^xxv^	119.5446 (1)	O1^liii^—O1—O3	88.8790 (1)
O2^xxviii^—Ba2—O2^xxiv^	64.0334 (1)	O1^liii^—O1—O3^xlviii^	88.8790 (1)
O2^xxviii^—Ba2—O2^xxiii^	0.0009 (1)	O1^liii^—O1—O3^xxv^	88.8619 (1)
O2^xxviii^—Ba2—O2^xxii^	171.1624 (10)	O1^liii^—O1—O3^i^	88.8619 (1)
O2^xxviii^—Ba2—O2^xxi^	55.6611 (1)	O1^liii^—O1—O3^xlvii^	117.371 (3)
O2^xxviii^—Ba2—O2^xx^	119.5450 (1)	O1^liii^—O1—O3^xix^	117.371 (3)
O2^xxviii^—Ba2—O2	119.5441 (1)	O1^liii^—O1—O3^iii^	117.339 (3)
O2^xxviii^—Ba2—O2^xix^	64.0338 (1)	O1^liii^—O1—O3^xxiv^	117.339 (3)
O2^xxviii^—Ba2—O3^xiii^	127.910 (3)	O1^liii^—O1—O1^i^	118.557
O2^xxviii^—Ba2—O3^xi^	127.886 (3)	O1^liii^—O1—O1^iii^	176.769
O2^xxviii^—Ba2—O3^xiv^	94.2779 (5)	O1^liii^—O1—O1^viii^	116.769
O2^xxviii^—Ba2—O3^xviii^	57.491 (4)	O1^liii^—O1—O1^ix^	178.557
O2^xxviii^—Ba2—O3^xv^	94.3137 (5)	O1^liii^—O1—Mn1	130.615 (3)
O2^xxviii^—Ba2—O3^xvii^	57.471 (4)	O1^liii^—O1—Mn1^viii^	130.615 (3)
O2^xxix^—Ba2—O2^xxviii^	112.345 (4)	O1^liii^—O1—O1^xlvii^	58.557
O2^xxix^—Ba2—O2^xxvii^	61.341 (2)	Mn2—O2—O2^xxv^	89.9911 (1)
O2^xxix^—Ba2—O2^vi^	112.350 (4)	Nb7—O2—Mn2	0.000
O2^xxix^—Ba2—O2^xxvi^	85.5885 (5)	Nb7—O2—O2^xxv^	89.9911 (1)
O2^xxix^—Ba2—O2^xxv^	61.335 (2)	Nb4—O2—Nb7	177.1144 (1)
O2^xxix^—Ba2—O2^xxiv^	85.5779 (5)	Nb4—O2—Mn2	177.1144 (1)
O2^xxix^—Ba2—O2^xxiii^	112.344 (4)	Nb4—O2—O2^xxv^	89.9924 (1)
O2^xxix^—Ba2—O2^xxii^	61.340 (2)	O2^xix^—O2—Nb4	134.888 (3)
O2^xxix^—Ba2—O2^xxi^	112.349 (4)	O2^xix^—O2—Nb7	43.117 (3)
O2^xxix^—Ba2—O2^xx^	85.5876 (5)	O2^xix^—O2—Mn2	43.117 (3)
O2^xxix^—Ba2—O2	61.334 (2)	O2^xix^—O2—O2^xxv^	120.000
O2^xxix^—Ba2—O2^xix^	85.5771 (5)	O2^xv^—O2—O2^xix^	60.011
O2^xxix^—Ba2—O3^xiii^	118.685 (6)	O2^xv^—O2—Nb4	134.871 (3)
O2^xxix^—Ba2—O3^xi^	118.703 (6)	O2^xv^—O2—Nb7	43.111 (3)
O2^xxix^—Ba2—O3^xiv^	118.696 (6)	O2^xv^—O2—Mn2	43.111 (3)
O2^xxix^—Ba2—O3^xviii^	168.0271 (5)	O2^xv^—O2—O2^xxv^	59.989
O2^xxix^—Ba2—O3^xv^	118.678 (6)	O2^xxiv^—O2—O2^xv^	60.000
O2^xxix^—Ba2—O3^xvii^	168.0271 (5)	O2^xxiv^—O2—O2^xix^	0.011
O2^xxx^—Ba2—O2^xxix^	0.0106 (1)	O2^xxiv^—O2—Nb4	134.894 (3)
O2^xxx^—Ba2—O2^xxviii^	112.350 (4)	O2^xxiv^—O2—Nb7	43.111 (3)
O2^xxx^—Ba2—O2^xxvii^	61.335 (2)	O2^xxiv^—O2—Mn2	43.111 (3)
O2^xxx^—Ba2—O2^vi^	112.345 (4)	O2^xxiv^—O2—O2^xxv^	119.989
O2^xxx^—Ba2—O2^xxvi^	85.5779 (5)	O2^xiv^—O2—O2^xxiv^	59.989
O2^xxx^—Ba2—O2^xxv^	61.341 (2)	O2^xiv^—O2—O2^xv^	0.0106 (1)
O2^xxx^—Ba2—O2^xxiv^	85.5885 (5)	O2^xiv^—O2—O2^xix^	60.000
O2^xxx^—Ba2—O2^xxiii^	112.349 (4)	O2^xiv^—O2—Nb4	134.877 (3)
O2^xxx^—Ba2—O2^xxii^	61.334 (2)	O2^xiv^—O2—Nb7	43.104 (3)
O2^xxx^—Ba2—O2^xxi^	112.344 (4)	O2^xiv^—O2—Mn2	43.104 (3)
O2^xxx^—Ba2—O2^xx^	85.5771 (5)	O2^xiv^—O2—O2^xxv^	60.000
O2^xxx^—Ba2—O2	61.340 (2)	O3^xv^—O2—O2^xiv^	88.5752 (1)
O2^xxx^—Ba2—O2^xix^	85.5876 (5)	O3^xv^—O2—O2^xxiv^	60.328 (3)
O2^xxx^—Ba2—O3^xiii^	118.678 (6)	O3^xv^—O2—O2^xv^	88.5811 (1)
O2^xxx^—Ba2—O3^xi^	118.696 (6)	O3^xv^—O2—O2^xix^	60.324 (3)
O2^xxx^—Ba2—O3^xiv^	118.703 (6)	O3^xv^—O2—Nb4	136.534 (3)
O2^xxx^—Ba2—O3^xviii^	168.0271 (5)	O3^xv^—O2—Nb7	45.618 (2)
O2^xxx^—Ba2—O3^xv^	118.685 (6)	O3^xv^—O2—Mn2	45.618 (2)
O2^xxx^—Ba2—O3^xvii^	168.0271 (5)	O3^xv^—O2—O2^xxv^	118.049 (3)
O2^xxxi^—Ba2—O2^xxx^	51.089 (6)	O3^xix^—O2—O3^xv^	56.108 (6)
O2^xxxi^—Ba2—O2^xxix^	51.095 (6)	O3^xix^—O2—O2^xiv^	60.321 (3)
O2^xxxi^—Ba2—O2^xxviii^	85.5779 (5)	O3^xix^—O2—O2^xxiv^	88.5755 (1)
O2^xxxi^—Ba2—O2^xxvii^	85.5885 (5)	O3^xix^—O2—O2^xv^	60.318 (3)
O2^xxxi^—Ba2—O2^vi^	61.335 (2)	O3^xix^—O2—O2^xix^	88.5814 (1)
O2^xxxi^—Ba2—O2^xxvi^	61.341 (2)	O3^xix^—O2—Nb4	136.518 (3)
O2^xxxi^—Ba2—O2^xxv^	112.350 (4)	O3^xix^—O2—Nb7	45.611 (2)
O2^xxxi^—Ba2—O2^xxiv^	112.345 (4)	O3^xix^—O2—Mn2	45.611 (2)
O2^xxxi^—Ba2—O2^xxiii^	85.5771 (5)	O3^xix^—O2—O2^xxv^	61.941 (3)
O2^xxxi^—Ba2—O2^xxii^	85.5876 (5)	O3^xiv^—O2—O3^xix^	56.119 (6)
O2^xxxi^—Ba2—O2^xxi^	61.334 (2)	O3^xiv^—O2—O3^xv^	0.0316 (1)
O2^xxxi^—Ba2—O2^xx^	61.340 (2)	O3^xiv^—O2—O2^xiv^	88.5574 (1)
O2^xxxi^—Ba2—O2	112.349 (4)	O3^xiv^—O2—O2^xxiv^	60.296 (3)
O2^xxxi^—Ba2—O2^xix^	112.344 (4)	O3^xiv^—O2—O2^xv^	88.5633 (1)
O2^xxxi^—Ba2—O3^xiii^	118.703 (6)	O3^xiv^—O2—O2^xix^	60.293 (3)
O2^xxxi^—Ba2—O3^xi^	118.685 (6)	O3^xiv^—O2—Nb4	136.552 (3)
O2^xxxi^—Ba2—O3^xiv^	168.0271 (5)	O3^xiv^—O2—Nb7	45.598 (2)
O2^xxxi^—Ba2—O3^xviii^	118.678 (6)	O3^xiv^—O2—Mn2	45.598 (2)
O2^xxxi^—Ba2—O3^xv^	168.0271 (5)	O3^xiv^—O2—O2^xxv^	118.060 (3)
O2^xxxi^—Ba2—O3^xvii^	118.696 (6)	O3^xxiv^—O2—O3^xiv^	56.130 (6)
O2^xxxii^—Ba2—O2^xxxi^	0.0106 (1)	O3^xxiv^—O2—O3^xix^	0.0316 (1)
O2^xxxii^—Ba2—O2^xxx^	51.083 (6)	O3^xxiv^—O2—O3^xv^	56.119 (6)
O2^xxxii^—Ba2—O2^xxix^	51.089 (6)	O3^xxiv^—O2—O2^xiv^	60.289 (3)
O2^xxxii^—Ba2—O2^xxviii^	85.5885 (5)	O3^xxiv^—O2—O2^xxiv^	88.5577 (1)
O2^xxxii^—Ba2—O2^xxvii^	85.5779 (5)	O3^xxiv^—O2—O2^xv^	60.286 (3)
O2^xxxii^—Ba2—O2^vi^	61.341 (2)	O3^xxiv^—O2—O2^xix^	88.5636 (1)
O2^xxxii^—Ba2—O2^xxvi^	61.335 (2)	O3^xxiv^—O2—Nb4	136.536 (3)
O2^xxxii^—Ba2—O2^xxv^	112.345 (4)	O3^xxiv^—O2—Nb7	45.592 (2)
O2^xxxii^—Ba2—O2^xxiv^	112.350 (4)	O3^xxiv^—O2—Mn2	45.592 (2)
O2^xxxii^—Ba2—O2^xxiii^	85.5876 (5)	O3^xxiv^—O2—O2^xxv^	61.930 (3)
O2^xxxii^—Ba2—O2^xxii^	85.5771 (5)	Ba2—O2—O3^xxiv^	115.080 (4)
O2^xxxii^—Ba2—O2^xxi^	61.340 (2)	Ba2—O2—O3^xiv^	59.051 (2)
O2^xxxii^—Ba2—O2^xx^	61.334 (2)	Ba2—O2—O3^xix^	115.067 (4)
O2^xxxii^—Ba2—O2	112.344 (4)	Ba2—O2—O3^xv^	59.060 (2)
O2^xxxii^—Ba2—O2^xix^	112.349 (4)	Ba2—O2—O2^xiv^	122.0171 (1)
O2^xxxii^—Ba2—O3^xiii^	118.696 (6)	Ba2—O2—O2^xxiv^	62.1803 (1)
O2^xxxii^—Ba2—O3^xi^	118.678 (6)	Ba2—O2—O2^xv^	122.0277 (1)
O2^xxxii^—Ba2—O3^xiv^	168.0271 (5)	Ba2—O2—O2^xix^	62.1697 (1)
O2^xxxii^—Ba2—O3^xviii^	118.685 (6)	Ba2—O2—Nb4	90.5539 (6)
O2^xxxii^—Ba2—O3^xv^	168.0271 (5)	Ba2—O2—Nb7	89.6838 (6)
O2^xxxii^—Ba2—O3^xvii^	118.703 (6)	Ba2—O2—Mn2	89.6838 (6)
O2^xxxiii^—Ba2—O2^xxxii^	51.095 (6)	Ba2—O2—O2^xxv^	175.5807 (5)
O2^xxxiii^—Ba2—O2^xxxi^	51.089 (6)	Ba2^xlix^—O2—Ba2	171.1624 (10)
O2^xxxiii^—Ba2—O2^xxx^	51.089 (6)	Ba2^xlix^—O2—O3^xxiv^	59.041 (2)
O2^xxxiii^—Ba2—O2^xxix^	51.083 (6)	Ba2^xlix^—O2—O3^xiv^	115.070 (4)
O2^xxxiii^—Ba2—O2^xxviii^	61.341 (2)	Ba2^xlix^—O2—O3^xix^	59.050 (2)
O2^xxxiii^—Ba2—O2^xxvii^	112.345 (4)	Ba2^xlix^—O2—O3^xv^	115.057 (4)
O2^xxxiii^—Ba2—O2^vi^	85.5885 (5)	Ba2^xlix^—O2—O2^xiv^	62.1692 (1)
O2^xxxiii^—Ba2—O2^xxvi^	112.350 (4)	Ba2^xlix^—O2—O2^xxiv^	122.0061 (1)
O2^xxxiii^—Ba2—O2^xxv^	85.5779 (5)	Ba2^xlix^—O2—O2^xv^	62.1587 (1)
O2^xxxiii^—Ba2—O2^xxiv^	61.335 (2)	Ba2^xlix^—O2—O2^xix^	122.0167 (1)
O2^xxxiii^—Ba2—O2^xxiii^	61.340 (2)	Ba2^xlix^—O2—Nb4	90.5386 (6)
O2^xxxiii^—Ba2—O2^xxii^	112.344 (4)	Ba2^xlix^—O2—Nb7	89.6661 (6)
O2^xxxiii^—Ba2—O2^xxi^	85.5876 (5)	Ba2^xlix^—O2—Mn2	89.6661 (6)
O2^xxxiii^—Ba2—O2^xx^	112.349 (4)	Ba2^xlix^—O2—O2^xxv^	4.4184 (5)
O2^xxxiii^—Ba2—O2	85.5771 (5)	Ba3—O2—Ba2^xlix^	85.6122 (5)
O2^xxxiii^—Ba2—O2^xix^	61.334 (2)	Ba3—O2—Ba2	85.6222 (5)
O2^xxxiii^—Ba2—O3^xiii^	168.0271 (5)	Ba3—O2—O3^xxiv^	60.059 (6)
O2^xxxiii^—Ba2—O3^xi^	168.0271 (5)	Ba3—O2—O3^xiv^	60.062 (6)
O2^xxxiii^—Ba2—O3^xiv^	118.678 (6)	Ba3—O2—O3^xix^	60.027 (6)
O2^xxxiii^—Ba2—O3^xviii^	118.703 (6)	Ba3—O2—O3^xv^	60.030 (6)
O2^xxxiii^—Ba2—O3^xv^	118.696 (6)	Ba3—O2—O2^xiv^	120.301 (3)
O2^xxxiii^—Ba2—O3^xvii^	118.685 (6)	Ba3—O2—O2^xxiv^	120.311 (3)
O2^xxxiv^—Ba2—O2^xxxiii^	0.0106 (1)	Ba3—O2—O2^xv^	120.297 (3)
O2^xxxiv^—Ba2—O2^xxxii^	51.089 (6)	Ba3—O2—O2^xix^	120.307 (3)
O2^xxxiv^—Ba2—O2^xxxi^	51.083 (6)	Ba3—O2—Nb4	89.793 (8)
O2^xxxiv^—Ba2—O2^xxx^	51.095 (6)	Ba3—O2—Nb7	93.093 (8)
O2^xxxiv^—Ba2—O2^xxix^	51.089 (6)	Ba3—O2—Mn2	93.093 (8)
O2^xxxiv^—Ba2—O2^xxviii^	61.335 (2)	Ba3—O2—O2^xxv^	89.9946 (1)
O2^xxxiv^—Ba2—O2^xxvii^	112.350 (4)	O2^xxii^—O2—Ba3	59.699 (3)
O2^xxxiv^—Ba2—O2^vi^	85.5779 (5)	O2^xxii^—O2—Ba2^xlix^	117.8308 (1)
O2^xxxiv^—Ba2—O2^xxvi^	112.345 (4)	O2^xxii^—O2—Ba2	57.9829 (1)
O2^xxxiv^—Ba2—O2^xxv^	85.5885 (5)	O2^xxii^—O2—O3^xxiv^	119.711 (3)
O2^xxxiv^—Ba2—O2^xxiv^	61.341 (2)	O2^xxii^—O2—O3^xiv^	91.4426 (1)
O2^xxxiv^—Ba2—O2^xxiii^	61.334 (2)	O2^xxii^—O2—O3^xix^	119.679 (3)
O2^xxxiv^—Ba2—O2^xxii^	112.349 (4)	O2^xxii^—O2—O3^xv^	91.4248 (1)
O2^xxxiv^—Ba2—O2^xxi^	85.5771 (5)	O2^xxii^—O2—O2^xiv^	180.000
O2^xxxiv^—Ba2—O2^xx^	112.344 (4)	O2^xxii^—O2—O2^xxiv^	120.011
O2^xxxiv^—Ba2—O2	85.5876 (5)	O2^xxii^—O2—O2^xv^	179.989
O2^xxxiv^—Ba2—O2^xix^	61.340 (2)	O2^xxii^—O2—O2^xix^	120.000
O2^xxxiv^—Ba2—O3^xiii^	168.0271 (5)	O2^xxii^—O2—Nb4	45.123 (3)
O2^xxxiv^—Ba2—O3^xi^	168.0271 (5)	O2^xxii^—O2—Nb7	136.896 (3)
O2^xxxiv^—Ba2—O3^xiv^	118.685 (6)	O2^xxii^—O2—Mn2	136.896 (3)
O2^xxxiv^—Ba2—O3^xviii^	118.696 (6)	O2^xxii^—O2—O2^xxv^	120.000
O2^xxxiv^—Ba2—O3^xv^	118.703 (6)	O2^xxvii^—O2—O2^xxii^	0.0093 (1)
O2^xxxiv^—Ba2—O3^xvii^	118.678 (6)	O2^xxvii^—O2—Ba3	59.696 (3)
O1^iii^—Ba3—O1^ix^	1.8642 (1)	O2^xxvii^—O2—Ba2^xlix^	117.8215 (1)
O1^v^—Ba3—O1^iii^	66.866 (6)	O2^xxvii^—O2—Ba2	57.9922 (1)
O1^v^—Ba3—O1^ix^	68.011 (6)	O2^xxvii^—O2—O3^xxiv^	119.708 (3)
O1^xi^—Ba3—O1^v^	1.8642 (1)	O2^xxvii^—O2—O3^xiv^	91.4478 (1)
O1^xi^—Ba3—O1^iii^	68.011 (6)	O2^xxvii^—O2—O3^xix^	119.677 (3)
O1^xi^—Ba3—O1^ix^	69.115 (7)	O2^xxvii^—O2—O3^xv^	91.4300 (1)
O1^xxxv^—Ba3—O1^xi^	68.011 (6)	O2^xxvii^—O2—O2^xiv^	179.9907 (1)
O1^xxxv^—Ba3—O1^v^	69.115 (7)	O2^xxvii^—O2—O2^xxiv^	120.020
O1^xxxv^—Ba3—O1^iii^	68.011 (6)	O2^xxvii^—O2—O2^xv^	179.980
O1^xxxv^—Ba3—O1^ix^	66.866 (6)	O2^xxvii^—O2—O2^xix^	120.009
O1^xxxvi^—Ba3—O1^xxxv^	1.8642 (1)	O2^xxvii^—O2—Nb4	45.117 (3)
O1^xxxvi^—Ba3—O1^xi^	66.866 (6)	O2^xxvii^—O2—Nb7	136.902 (3)
O1^xxxvi^—Ba3—O1^v^	68.011 (6)	O2^xxvii^—O2—Mn2	136.902 (3)
O1^xxxvi^—Ba3—O1^iii^	69.115 (7)	O2^xxvii^—O2—O2^xxv^	119.991
O1^xxxvi^—Ba3—O1^ix^	68.011 (6)	O2^xli^—O2—O2^xxvii^	60.000
O3^xx^—Ba3—O1^xxxvi^	94.5725 (5)	O2^xli^—O2—O2^xxii^	60.009
O3^xx^—Ba3—O1^xxxv^	96.4367 (4)	O2^xli^—O2—Ba3	59.696 (3)
O3^xx^—Ba3—O1^xi^	58.033 (4)	O2^xli^—O2—Ba2^xlix^	57.9740 (1)
O3^xx^—Ba3—O1^v^	59.001 (4)	O2^xli^—O2—Ba2	117.8396 (1)
O3^xx^—Ba3—O1^iii^	125.578 (2)	O2^xli^—O2—O3^xxiv^	91.4312 (1)
O3^xx^—Ba3—O1^ix^	126.855 (2)	O2^xli^—O2—O3^xiv^	119.710 (3)
O3^xiii^—Ba3—O3^xx^	52.5375 (1)	O2^xli^—O2—O3^xix^	91.4134 (1)
O3^xiii^—Ba3—O1^xxxvi^	125.578 (2)	O2^xli^—O2—O3^xv^	119.678 (3)
O3^xiii^—Ba3—O1^xxxv^	126.855 (2)	O2^xli^—O2—O2^xiv^	119.991
O3^xiii^—Ba3—O1^xi^	59.001 (4)	O2^xli^—O2—O2^xxiv^	179.9801 (1)
O3^xiii^—Ba3—O1^v^	58.033 (4)	O2^xli^—O2—O2^xv^	119.980
O3^xiii^—Ba3—O1^iii^	94.5725 (5)	O2^xli^—O2—O2^xix^	179.9907 (1)
O3^xiii^—Ba3—O1^ix^	96.4367 (4)	O2^xli^—O2—Nb4	45.117 (3)
O3^xix^—Ba3—O3^xiii^	119.4895 (1)	O2^xli^—O2—Nb7	136.877 (3)
O3^xix^—Ba3—O3^xx^	168.9878 (9)	O2^xli^—O2—Mn2	136.877 (3)
O3^xix^—Ba3—O1^xxxvi^	96.4367 (4)	O2^xli^—O2—O2^xxv^	59.991
O3^xix^—Ba3—O1^xxxv^	94.5725 (5)	O2^xlii^—O2—O2^xli^	0.0093 (1)
O3^xix^—Ba3—O1^xi^	126.855 (2)	O2^xlii^—O2—O2^xxvii^	59.991
O3^xix^—Ba3—O1^v^	125.578 (2)	O2^xlii^—O2—O2^xxii^	60.000
O3^xix^—Ba3—O1^iii^	59.001 (4)	O2^xlii^—O2—Ba3	59.693 (3)
O3^xix^—Ba3—O1^ix^	58.033 (4)	O2^xlii^—O2—Ba2^xlix^	57.9833 (1)
O3^xxxvii^—Ba3—O3^xix^	67.1192 (1)	O2^xlii^—O2—Ba2	117.8303 (1)
O3^xxxvii^—Ba3—O3^xiii^	168.9878 (9)	O2^xlii^—O2—O3^xxiv^	91.4364 (1)
O3^xxxvii^—Ba3—O3^xx^	119.4895 (1)	O2^xlii^—O2—O3^xiv^	119.707 (3)
O3^xxxvii^—Ba3—O1^xxxvi^	59.001 (4)	O2^xlii^—O2—O3^xix^	91.4186 (1)
O3^xxxvii^—Ba3—O1^xxxv^	58.033 (4)	O2^xlii^—O2—O3^xv^	119.676 (3)
O3^xxxvii^—Ba3—O1^xi^	125.578 (2)	O2^xlii^—O2—O2^xiv^	120.000
O3^xxxvii^—Ba3—O1^v^	126.855 (2)	O2^xlii^—O2—O2^xxiv^	179.9894 (1)
O3^xxxvii^—Ba3—O1^iii^	96.4367 (4)	O2^xlii^—O2—O2^xv^	119.989
O3^xxxvii^—Ba3—O1^ix^	94.5725 (5)	O2^xlii^—O2—O2^xix^	180.000
O3^xxxviii^—Ba3—O3^xxxvii^	52.5375 (1)	O2^xlii^—O2—Nb4	45.112 (3)
O3^xxxviii^—Ba3—O3^xix^	119.4895 (1)	O2^xlii^—O2—Nb7	136.883 (3)
O3^xxxviii^—Ba3—O3^xiii^	119.4895 (1)	O2^xlii^—O2—Mn2	136.883 (3)
O3^xxxviii^—Ba3—O3^xx^	67.1192 (1)	O2^xlii^—O2—O2^xxv^	60.000
O3^xxxviii^—Ba3—O1^xxxvi^	58.033 (4)	O2^xxix^—O2—O2^xlii^	90.000
O3^xxxviii^—Ba3—O1^xxxv^	59.001 (4)	O2^xxix^—O2—O2^xli^	90.0054 (1)
O3^xxxviii^—Ba3—O1^xi^	94.5725 (5)	O2^xxix^—O2—O2^xxvii^	60.144 (3)
O3^xxxviii^—Ba3—O1^v^	96.4367 (4)	O2^xxix^—O2—O2^xxii^	60.141 (3)
O3^xxxviii^—Ba3—O1^iii^	126.855 (2)	O2^xxix^—O2—Ba3	119.839 (6)
O3^xxxviii^—Ba3—O1^ix^	125.578 (2)	O2^xxix^—O2—Ba2^xlix^	122.777 (2)
O3^xv^—Ba3—O3^xxxviii^	168.9878 (9)	O2^xxix^—O2—Ba2	63.167 (4)
O3^xv^—Ba3—O3^xxxvii^	119.4895 (1)	O2^xxix^—O2—O3^xxiv^	178.1451 (1)
O3^xv^—Ba3—O3^xix^	52.5375 (1)	O2^xxix^—O2—O3^xiv^	122.079 (6)
O3^xv^—Ba3—O3^xiii^	67.1192 (1)	O2^xxix^—O2—O3^xix^	178.1420 (1)
O3^xv^—Ba3—O3^xx^	119.4895 (1)	O2^xxix^—O2—O3^xv^	122.090 (6)
O3^xv^—Ba3—O1^xxxvi^	126.855 (2)	O2^xxix^—O2—O2^xiv^	119.859 (3)
O3^xv^—Ba3—O1^xxxv^	125.578 (2)	O2^xxix^—O2—O2^xxiv^	90.0061 (1)
O3^xv^—Ba3—O1^xi^	96.4367 (4)	O2^xxix^—O2—O2^xv^	119.862 (3)
O3^xv^—Ba3—O1^v^	94.5725 (5)	O2^xxix^—O2—O2^xix^	90.000
O3^xv^—Ba3—O1^iii^	58.033 (4)	O2^xxix^—O2—Nb4	44.888 (3)
O3^xv^—Ba3—O1^ix^	59.001 (4)	O2^xxix^—O2—Nb7	133.044 (3)
O3^xxvi^—Ba3—O3^xv^	119.4920 (1)	O2^xxix^—O2—Mn2	133.044 (3)
O3^xxvi^—Ba3—O3^xxxviii^	67.1171 (1)	O2^xxix^—O2—O2^xxv^	119.859 (3)
O3^xxvi^—Ba3—O3^xxxvii^	119.4877 (1)	O2^xliv^—O2—O2^xxix^	59.727 (6)
O3^xxvi^—Ba3—O3^xix^	168.9911 (9)	O2^xliv^—O2—O2^xlii^	60.138 (3)
O3^xxvi^—Ba3—O3^xiii^	52.5397 (1)	O2^xliv^—O2—O2^xli^	60.135 (3)
O3^xxvi^—Ba3—O3^xx^	0.0033 (1)	O2^xliv^—O2—O2^xxvii^	90.000
O3^xxvi^—Ba3—O1^xxxvi^	94.5692 (5)	O2^xliv^—O2—O2^xxii^	90.0054 (1)
O3^xxvi^—Ba3—O1^xxxv^	96.4334 (4)	O2^xliv^—O2—Ba3	119.830 (6)
O3^xxvi^—Ba3—O1^xi^	58.031 (4)	O2^xliv^—O2—Ba2^xlix^	63.157 (4)
O3^xxvi^—Ba3—O1^v^	58.999 (4)	O2^xliv^—O2—Ba2	122.787 (2)
O3^xxvi^—Ba3—O1^iii^	125.577 (2)	O2^xliv^—O2—O3^xxiv^	122.059 (6)
O3^xxvi^—Ba3—O1^ix^	126.854 (2)	O2^xliv^—O2—O3^xiv^	178.1258 (1)
O3^xi^—Ba3—O3^xxvi^	52.5420 (1)	O2^xliv^—O2—O3^xix^	122.070 (6)
O3^xi^—Ba3—O3^xv^	67.1171 (1)	O2^xliv^—O2—O3^xv^	178.1226 (1)
O3^xi^—Ba3—O3^xxxviii^	119.4920 (1)	O2^xliv^—O2—O2^xiv^	89.9946 (1)
O3^xi^—Ba3—O3^xxxvii^	168.9911 (9)	O2^xliv^—O2—O2^xxiv^	119.858 (3)
O3^xi^—Ba3—O3^xix^	119.4877 (1)	O2^xliv^—O2—O2^xv^	89.9885 (1)
O3^xi^—Ba3—O3^xiii^	0.0033 (1)	O2^xliv^—O2—O2^xix^	119.862 (3)
O3^xi^—Ba3—O3^xx^	52.5397 (1)	O2^xliv^—O2—Nb4	44.883 (3)
O3^xi^—Ba3—O1^xxxvi^	125.577 (2)	O2^xliv^—O2—Nb7	133.027 (3)
O3^xi^—Ba3—O1^xxxv^	126.854 (2)	O2^xliv^—O2—Mn2	133.027 (3)
O3^xi^—Ba3—O1^xi^	58.999 (4)	O2^xliv^—O2—O2^xxv^	60.132 (3)
O3^xi^—Ba3—O1^v^	58.031 (4)	O2^xxx^—O2—O2^xliv^	59.730 (6)
O3^xi^—Ba3—O1^iii^	94.5692 (5)	O2^xxx^—O2—O2^xxix^	0.0093 (1)
O3^xi^—Ba3—O1^ix^	96.4334 (4)	O2^xxx^—O2—O2^xlii^	89.9946 (1)
O3^xxiv^—Ba3—O3^xi^	119.4901 (1)	O2^xxx^—O2—O2^xli^	90.000
O3^xxiv^—Ba3—O3^xxvi^	168.9944 (9)	O2^xxx^—O2—O2^xxvii^	60.135 (3)
O3^xxiv^—Ba3—O3^xv^	52.5397 (1)	O2^xxx^—O2—O2^xxii^	60.132 (3)
O3^xxiv^—Ba3—O3^xxxviii^	119.4877 (1)	O2^xxx^—O2—Ba3	119.830 (6)
O3^xxiv^—Ba3—O3^xxxvii^	67.1171 (1)	O2^xxx^—O2—Ba2^xlix^	122.779 (2)
O3^xxiv^—Ba3—O3^xix^	0.0033 (1)	O2^xxx^—O2—Ba2	63.163 (4)
O3^xxiv^—Ba3—O3^xiii^	119.4920 (1)	O2^xxx^—O2—O3^xxiv^	178.1444 (1)
O3^xxiv^—Ba3—O3^xx^	168.9911 (9)	O2^xxx^—O2—O3^xiv^	122.076 (6)
O3^xxiv^—Ba3—O1^xxxvi^	96.4334 (4)	O2^xxx^—O2—O3^xix^	178.1412 (1)
O3^xxiv^—Ba3—O1^xxxv^	94.5692 (5)	O2^xxx^—O2—O3^xv^	122.087 (6)
O3^xxiv^—Ba3—O1^xi^	126.854 (2)	O2^xxx^—O2—O2^xiv^	119.868 (3)
O3^xxiv^—Ba3—O1^v^	125.577 (2)	O2^xxx^—O2—O2^xxiv^	90.0115 (1)
O3^xxiv^—Ba3—O1^iii^	58.999 (4)	O2^xxx^—O2—O2^xv^	119.872 (3)
O3^xxiv^—Ba3—O1^ix^	58.031 (4)	O2^xxx^—O2—O2^xix^	90.0054 (1)
O3^xxxix^—Ba3—O3^xxiv^	67.1151 (1)	O2^xxx^—O2—Nb4	44.883 (3)
O3^xxxix^—Ba3—O3^xi^	168.9944 (9)	O2^xxx^—O2—Nb7	133.050 (3)
O3^xxxix^—Ba3—O3^xxvi^	119.4901 (1)	O2^xxx^—O2—Mn2	133.050 (3)
O3^xxxix^—Ba3—O3^xv^	119.4877 (1)	O2^xxx^—O2—O2^xxv^	119.862 (3)
O3^xxxix^—Ba3—O3^xxxviii^	52.5397 (1)	O2^xliii^—O2—O2^xxx^	59.733 (6)
O3^xxxix^—Ba3—O3^xxxvii^	0.0033 (1)	O2^xliii^—O2—O2^xliv^	0.0093 (1)
O3^xxxix^—Ba3—O3^xix^	67.1171 (1)	O2^xliii^—O2—O2^xxix^	59.730 (6)
O3^xxxix^—Ba3—O3^xiii^	168.9911 (9)	O2^xliii^—O2—O2^xlii^	60.129 (3)
O3^xxxix^—Ba3—O3^xx^	119.4920 (1)	O2^xliii^—O2—O2^xli^	60.126 (3)
O3^xxxix^—Ba3—O1^xxxvi^	58.999 (4)	O2^xliii^—O2—O2^xxvii^	89.9946 (1)
O3^xxxix^—Ba3—O1^xxxv^	58.031 (4)	O2^xliii^—O2—O2^xxii^	90.000
O3^xxxix^—Ba3—O1^xi^	125.577 (2)	O2^xliii^—O2—Ba3	119.821 (6)
O3^xxxix^—Ba3—O1^v^	126.854 (2)	O2^xliii^—O2—Ba2^xlix^	63.154 (4)
O3^xxxix^—Ba3—O1^iii^	96.4334 (4)	O2^xliii^—O2—Ba2	122.789 (2)
O3^xxxix^—Ba3—O1^ix^	94.5692 (5)	O2^xliii^—O2—O3^xxiv^	122.056 (6)
O3^xl^—Ba3—O3^xxxix^	52.5420 (1)	O2^xliii^—O2—O3^xiv^	178.1251 (1)
O3^xl^—Ba3—O3^xxiv^	119.4901 (1)	O2^xliii^—O2—O3^xix^	122.067 (6)
O3^xl^—Ba3—O3^xi^	119.4901 (1)	O2^xliii^—O2—O3^xv^	178.1218 (1)
O3^xl^—Ba3—O3^xxvi^	67.1151 (1)	O2^xliii^—O2—O2^xiv^	90.000
O3^xl^—Ba3—O3^xv^	168.9911 (9)	O2^xliii^—O2—O2^xxiv^	119.868 (3)
O3^xl^—Ba3—O3^xxxviii^	0.0033 (1)	O2^xliii^—O2—O2^xv^	89.9939 (1)
O3^xl^—Ba3—O3^xxxvii^	52.5397 (1)	O2^xliii^—O2—O2^xix^	119.871 (3)
O3^xl^—Ba3—O3^xix^	119.4920 (1)	O2^xliii^—O2—Nb4	44.877 (3)
O3^xl^—Ba3—O3^xiii^	119.4877 (1)	O2^xliii^—O2—Nb7	133.032 (3)
O3^xl^—Ba3—O3^xx^	67.1171 (1)	O2^xliii^—O2—Mn2	133.032 (3)
O3^xl^—Ba3—O1^xxxvi^	58.031 (4)	O2^xliii^—O2—O2^xxv^	60.129 (3)
O3^xl^—Ba3—O1^xxxv^	58.999 (4)	Ba2^xxxiii^—O2—O2^xliii^	55.505 (6)
O3^xl^—Ba3—O1^xi^	94.5692 (5)	Ba2^xxxiii^—O2—O2^xxx^	55.508 (6)
O3^xl^—Ba3—O1^v^	96.4334 (4)	Ba2^xxxiii^—O2—O2^xliv^	55.496 (6)
O3^xl^—Ba3—O1^iii^	126.854 (2)	Ba2^xxxiii^—O2—O2^xxix^	55.499 (6)
O3^xl^—Ba3—O1^ix^	125.577 (2)	Ba2^xxxiii^—O2—O2^xlii^	115.542 (3)
O3^xiv^—Ba3—O3^xl^	168.9944 (9)	Ba2^xxxiii^—O2—O2^xli^	115.539 (3)
O3^xiv^—Ba3—O3^xxxix^	119.4901 (1)	Ba2^xxxiii^—O2—O2^xxvii^	115.550 (3)
O3^xiv^—Ba3—O3^xxiv^	52.5420 (1)	Ba2^xxxiii^—O2—O2^xxii^	115.547 (3)
O3^xiv^—Ba3—O3^xi^	67.1151 (1)	Ba2^xxxiii^—O2—Ba3	174.2255 (3)
O3^xiv^—Ba3—O3^xxvi^	119.4901 (1)	Ba2^xxxiii^—O2—Ba2^xlix^	94.4115 (5)
O3^xiv^—Ba3—O3^xv^	0.0033 (1)	Ba2^xxxiii^—O2—Ba2	94.4229 (5)
O3^xiv^—Ba3—O3^xxxviii^	168.9911 (9)	Ba2^xxxiii^—O2—O3^xxiv^	124.729 (6)
O3^xiv^—Ba3—O3^xxxvii^	119.4920 (1)	Ba2^xxxiii^—O2—O3^xiv^	124.739 (6)
O3^xiv^—Ba3—O3^xix^	52.5397 (1)	Ba2^xxxiii^—O2—O3^xix^	124.761 (6)
O3^xiv^—Ba3—O3^xiii^	67.1171 (1)	Ba2^xxxiii^—O2—O3^xv^	124.771 (6)
O3^xiv^—Ba3—O3^xx^	119.4877 (1)	Ba2^xxxiii^—O2—O2^xiv^	64.453 (3)
O3^xiv^—Ba3—O1^xxxvi^	126.854 (2)	Ba2^xxxiii^—O2—O2^xxiv^	64.455 (3)
O3^xiv^—Ba3—O1^xxxv^	125.577 (2)	Ba2^xxxiii^—O2—O2^xv^	64.455 (3)
O3^xiv^—Ba3—O1^xi^	96.4334 (4)	Ba2^xxxiii^—O2—O2^xix^	64.458 (3)
O3^xiv^—Ba3—O1^v^	94.5692 (5)	Ba2^xxxiii^—O2—Nb4	84.432 (7)
O3^xiv^—Ba3—O1^iii^	58.031 (4)	Ba2^xxxiii^—O2—Nb7	92.682 (7)
O3^xiv^—Ba3—O1^ix^	58.999 (4)	Ba2^xxxiii^—O2—Mn2	92.682 (7)
O2^xli^—Ba3—O3^xiv^	115.173 (4)	Ba2^xxxiii^—O2—O2^xxv^	89.9947 (1)
O2^xli^—Ba3—O3^xl^	54.614 (3)	Mn1—O3—O3^xxv^	89.9739 (1)
O2^xli^—Ba3—O3^xxxix^	54.608 (3)	Nb7—O3—Mn1	79.257 (8)
O2^xli^—Ba3—O3^xxiv^	88.7756 (8)	Nb7—O3—O3^xxv^	89.9746 (1)
O2^xli^—Ba3—O3^xi^	115.180 (4)	Mn2—O3—Nb7	0.000
O2^xli^—Ba3—O3^xxvi^	88.7865 (8)	Mn2—O3—Mn1	79.257 (8)
O2^xli^—Ba3—O3^xv^	115.170 (4)	Mn2—O3—O3^xxv^	89.9746 (1)
O2^xli^—Ba3—O3^xxxviii^	54.611 (3)	O3^xiv^—O3—Mn2	48.867 (3)
O2^xli^—Ba3—O3^xxxvii^	54.605 (3)	O3^xiv^—O3—Nb7	48.867 (3)
O2^xli^—Ba3—O3^xix^	88.7749 (8)	O3^xiv^—O3—Mn1	47.500 (3)
O2^xli^—Ba3—O3^xiii^	115.177 (4)	O3^xiv^—O3—O3^xxv^	120.000
O2^xli^—Ba3—O3^xx^	88.7857 (8)	O3^xv^—O3—O3^xiv^	0.0335 (1)
O2^xli^—Ba3—O1^xxxvi^	104.145 (8)	O3^xv^—O3—Mn2	48.850 (3)
O2^xli^—Ba3—O1^xxxv^	104.145 (8)	O3^xv^—O3—Nb7	48.850 (3)
O2^xli^—Ba3—O1^xi^	143.173 (3)	O3^xv^—O3—Mn1	47.483 (3)
O2^xli^—Ba3—O1^v^	144.772 (3)	O3^xv^—O3—O3^xxv^	119.967
O2^xli^—Ba3—O1^iii^	144.761 (3)	O3^xxiv^—O3—O3^xv^	60.000
O2^xli^—Ba3—O1^ix^	143.162 (3)	O3^xxiv^—O3—O3^xiv^	60.033
O2^xlii^—Ba3—O2^xli^	0.0109 (1)	O3^xxiv^—O3—Mn2	48.850 (3)
O2^xlii^—Ba3—O3^xiv^	115.180 (4)	O3^xxiv^—O3—Nb7	48.850 (3)
O2^xlii^—Ba3—O3^xl^	54.608 (3)	O3^xxiv^—O3—Mn1	47.483 (3)
O2^xlii^—Ba3—O3^xxxix^	54.614 (3)	O3^xxiv^—O3—O3^xxv^	59.967
O2^xlii^—Ba3—O3^xxiv^	88.7865 (8)	O3^xix^—O3—O3^xxiv^	0.0335 (1)
O2^xlii^—Ba3—O3^xi^	115.173 (4)	O3^xix^—O3—O3^xv^	59.967
O2^xlii^—Ba3—O3^xxvi^	88.7756 (8)	O3^xix^—O3—O3^xiv^	60.000
O2^xlii^—Ba3—O3^xv^	115.177 (4)	O3^xix^—O3—Mn2	48.833 (3)
O2^xlii^—Ba3—O3^xxxviii^	54.605 (3)	O3^xix^—O3—Nb7	48.833 (3)
O2^xlii^—Ba3—O3^xxxvii^	54.611 (3)	O3^xix^—O3—Mn1	47.465 (3)
O2^xlii^—Ba3—O3^xix^	88.7857 (8)	O3^xix^—O3—O3^xxv^	60.000
O2^xlii^—Ba3—O3^xiii^	115.170 (4)	O2^xxiv^—O3—O3^xix^	91.4248 (1)
O2^xlii^—Ba3—O3^xx^	88.7749 (8)	O2^xxiv^—O3—O3^xxiv^	91.4435 (1)
O2^xlii^—Ba3—O1^xxxvi^	104.145 (8)	O2^xxiv^—O3—O3^xv^	61.963 (3)
O2^xlii^—Ba3—O1^xxxv^	104.145 (8)	O2^xxiv^—O3—O3^xiv^	61.951 (3)
O2^xlii^—Ba3—O1^xi^	143.162 (3)	O2^xxiv^—O3—Mn2	42.718 (3)
O2^xlii^—Ba3—O1^v^	144.761 (3)	O2^xxiv^—O3—Nb7	42.718 (3)
O2^xlii^—Ba3—O1^iii^	144.772 (3)	O2^xxiv^—O3—Mn1	108.575 (6)
O2^xlii^—Ba3—O1^ix^	143.173 (3)	O2^xxiv^—O3—O3^xxv^	119.676 (3)
O2^xxvii^—Ba3—O2^xlii^	60.602 (6)	O2^xix^—O3—O2^xxiv^	0.0107 (1)
O2^xxvii^—Ba3—O2^xli^	60.608 (6)	O2^xix^—O3—O3^xix^	91.4186 (1)
O2^xxvii^—Ba3—O3^xiv^	88.7865 (8)	O2^xix^—O3—O3^xxiv^	91.4373 (1)
O2^xxvii^—Ba3—O3^xl^	88.7756 (8)	O2^xix^—O3—O3^xv^	61.952 (3)
O2^xxvii^—Ba3—O3^xxxix^	115.173 (4)	O2^xix^—O3—O3^xiv^	61.941 (3)
O2^xxvii^—Ba3—O3^xxiv^	115.180 (4)	O2^xix^—O3—Mn2	42.712 (3)
O2^xxvii^—Ba3—O3^xi^	54.614 (3)	O2^xix^—O3—Nb7	42.712 (3)
O2^xxvii^—Ba3—O3^xxvi^	54.608 (3)	O2^xix^—O3—Mn1	108.565 (6)
O2^xxvii^—Ba3—O3^xv^	88.7857 (8)	O2^xix^—O3—O3^xxv^	119.679 (3)
O2^xxvii^—Ba3—O3^xxxviii^	88.7749 (8)	O2^xiv^—O3—O2^xix^	59.386 (6)
O2^xxvii^—Ba3—O3^xxxvii^	115.170 (4)	O2^xiv^—O3—O2^xxiv^	59.383 (6)
O2^xxvii^—Ba3—O3^xix^	115.177 (4)	O2^xiv^—O3—O3^xix^	61.940 (3)
O2^xxvii^—Ba3—O3^xiii^	54.611 (3)	O2^xiv^—O3—O3^xxiv^	61.929 (3)
O2^xxvii^—Ba3—O3^xx^	54.605 (3)	O2^xiv^—O3—O3^xv^	91.4239 (1)
O2^xxvii^—Ba3—O1^xxxvi^	143.162 (3)	O2^xiv^—O3—O3^xiv^	91.4426 (1)
O2^xxvii^—Ba3—O1^xxxv^	144.761 (3)	O2^xiv^—O3—Mn2	42.700 (3)
O2^xxvii^—Ba3—O1^xi^	104.145 (8)	O2^xiv^—O3—Nb7	42.700 (3)
O2^xxvii^—Ba3—O1^v^	104.145 (8)	O2^xiv^—O3—Mn1	108.542 (6)
O2^xxvii^—Ba3—O1^iii^	143.173 (3)	O2^xiv^—O3—O3^xxv^	60.293 (3)
O2^xxvii^—Ba3—O1^ix^	144.772 (3)	O2^xv^—O3—O2^xiv^	0.0107 (1)
O2^xxii^—Ba3—O2^xxvii^	0.0109 (1)	O2^xv^—O3—O2^xix^	59.390 (6)
O2^xxii^—Ba3—O2^xlii^	60.608 (6)	O2^xv^—O3—O2^xxiv^	59.386 (6)
O2^xxii^—Ba3—O2^xli^	60.614 (6)	O2^xv^—O3—O3^xix^	61.930 (3)
O2^xxii^—Ba3—O3^xiv^	88.7756 (8)	O2^xv^—O3—O3^xxiv^	61.918 (3)
O2^xxii^—Ba3—O3^xl^	88.7865 (8)	O2^xv^—O3—O3^xv^	91.4177 (1)
O2^xxii^—Ba3—O3^xxxix^	115.180 (4)	O2^xv^—O3—O3^xiv^	91.4364 (1)
O2^xxii^—Ba3—O3^xxiv^	115.173 (4)	O2^xv^—O3—Mn2	42.695 (3)
O2^xxii^—Ba3—O3^xi^	54.608 (3)	O2^xv^—O3—Nb7	42.695 (3)
O2^xxii^—Ba3—O3^xxvi^	54.614 (3)	O2^xv^—O3—Mn1	108.531 (6)
O2^xxii^—Ba3—O3^xv^	88.7749 (8)	O2^xv^—O3—O3^xxv^	60.289 (3)
O2^xxii^—Ba3—O3^xxxviii^	88.7857 (8)	Ba2^l^—O3—O2^xv^	63.468 (6)
O2^xxii^—Ba3—O3^xxxvii^	115.177 (4)	Ba2^l^—O3—O2^xiv^	63.458 (6)
O2^xxii^—Ba3—O3^xix^	115.170 (4)	Ba2^l^—O3—O2^xix^	63.479 (6)
O2^xxii^—Ba3—O3^xiii^	54.605 (3)	Ba2^l^—O3—O2^xxiv^	63.469 (6)
O2^xxii^—Ba3—O3^xx^	54.611 (3)	Ba2^l^—O3—O3^xix^	125.273 (3)
O2^xxii^—Ba3—O1^xxxvi^	143.173 (3)	Ba2^l^—O3—O3^xxiv^	125.260 (3)
O2^xxii^—Ba3—O1^xxxv^	144.772 (3)	Ba2^l^—O3—O3^xv^	125.309 (3)
O2^xxii^—Ba3—O1^xi^	104.145 (8)	Ba2^l^—O3—O3^xiv^	125.295 (3)
O2^xxii^—Ba3—O1^v^	104.145 (8)	Ba2^l^—O3—Mn2	91.285 (8)
O2^xxii^—Ba3—O1^iii^	143.162 (3)	Ba2^l^—O3—Nb7	91.285 (8)
O2^xxii^—Ba3—O1^ix^	144.761 (3)	Ba2^l^—O3—Mn1	170.5417 (1)
O2^xxv^—Ba3—O2^xxii^	60.608 (6)	Ba2^l^—O3—O3^xxv^	89.9821 (1)
O2^xxv^—Ba3—O2^xxvii^	60.614 (6)	O1^viii^—O3—Ba2^l^	143.546 (3)
O2^xxv^—Ba3—O2^xlii^	60.608 (6)	O1^viii^—O3—O2^xv^	150.486 (3)
O2^xxv^—Ba3—O2^xli^	60.602 (6)	O1^viii^—O3—O2^xiv^	150.496 (3)
O2^xxv^—Ba3—O3^xiv^	54.614 (3)	O1^viii^—O3—O2^xix^	114.930 (7)
O2^xxv^—Ba3—O3^xl^	115.173 (4)	O1^viii^—O3—O2^xxiv^	114.939 (7)
O2^xxv^—Ba3—O3^xxxix^	88.7756 (8)	O1^viii^—O3—O3^xix^	90.5864 (1)
O2^xxv^—Ba3—O3^xxiv^	54.608 (3)	O1^viii^—O3—O3^xxiv^	90.6037 (1)
O2^xxv^—Ba3—O3^xi^	88.7865 (8)	O1^viii^—O3—O3^xv^	63.723 (3)
O2^xxv^—Ba3—O3^xxvi^	115.180 (4)	O1^viii^—O3—O3^xiv^	63.713 (3)
O2^xxv^—Ba3—O3^xv^	54.611 (3)	O1^viii^—O3—Mn2	111.515 (6)
O2^xxv^—Ba3—O3^xxxviii^	115.170 (4)	O1^viii^—O3—Nb7	111.515 (6)
O2^xxv^—Ba3—O3^xxxvii^	88.7749 (8)	O1^viii^—O3—Mn1	43.127 (3)
O2^xxv^—Ba3—O3^xix^	54.605 (3)	O1^viii^—O3—O3^xxv^	116.943 (3)
O2^xxv^—Ba3—O3^xiii^	88.7857 (8)	O1^xlvii^—O3—O1^viii^	53.918 (6)
O2^xxv^—Ba3—O3^xx^	115.177 (4)	O1^xlvii^—O3—Ba2^l^	143.497 (3)
O2^xxv^—Ba3—O1^xxxvi^	144.761 (3)	O1^xlvii^—O3—O2^xv^	114.877 (7)
O2^xxv^—Ba3—O1^xxxv^	143.162 (3)	O1^xlvii^—O3—O2^xiv^	114.886 (7)
O2^xxv^—Ba3—O1^xi^	144.772 (3)	O1^xlvii^—O3—O2^xix^	150.492 (3)
O2^xxv^—Ba3—O1^v^	143.173 (3)	O1^xlvii^—O3—O2^xxiv^	150.502 (3)
O2^xxv^—Ba3—O1^iii^	104.145 (8)	O1^xlvii^—O3—O3^xix^	63.701 (3)
O2^xxv^—Ba3—O1^ix^	104.145 (8)	O1^xlvii^—O3—O3^xxiv^	63.691 (3)
O2—Ba3—O2^xxv^	0.0109 (1)	O1^xlvii^—O3—O3^xv^	90.5867 (1)
O2—Ba3—O2^xxii^	60.602 (6)	O1^xlvii^—O3—O3^xiv^	90.6040 (1)
O2—Ba3—O2^xxvii^	60.608 (6)	O1^xlvii^—O3—Mn2	111.484 (6)
O2—Ba3—O2^xlii^	60.614 (6)	O1^xlvii^—O3—Nb7	111.484 (6)
O2—Ba3—O2^xli^	60.608 (6)	O1^xlvii^—O3—Mn1	43.110 (3)
O2—Ba3—O3^xiv^	54.608 (3)	O1^xlvii^—O3—O3^xxv^	63.025 (3)
O2—Ba3—O3^xl^	115.180 (4)	O1^i^—O3—O1^xlvii^	54.564 (6)
O2—Ba3—O3^xxxix^	88.7865 (8)	O1^i^—O3—O1^viii^	1.6847 (1)
O2—Ba3—O3^xxiv^	54.614 (3)	O1^i^—O3—Ba2^l^	144.218 (3)
O2—Ba3—O3^xi^	88.7756 (8)	O1^i^—O3—O2^xv^	149.098 (3)
O2—Ba3—O3^xxvi^	115.173 (4)	O1^i^—O3—O2^xiv^	149.107 (3)
O2—Ba3—O3^xv^	54.605 (3)	O1^i^—O3—O2^xix^	113.565 (7)
O2—Ba3—O3^xxxviii^	115.177 (4)	O1^i^—O3—O2^xxiv^	113.574 (7)
O2—Ba3—O3^xxxvii^	88.7857 (8)	O1^i^—O3—O3^xix^	89.6383 (1)
O2—Ba3—O3^xix^	54.611 (3)	O1^i^—O3—O3^xxiv^	89.6563 (1)
O2—Ba3—O3^xiii^	88.7749 (8)	O1^i^—O3—O3^xv^	62.038 (3)
O2—Ba3—O3^xx^	115.170 (4)	O1^i^—O3—O3^xiv^	62.028 (3)
O2—Ba3—O1^xxxvi^	144.772 (3)	O1^i^—O3—Mn2	109.858 (6)
O2—Ba3—O1^xxxv^	143.173 (3)	O1^i^—O3—Nb7	109.858 (6)
O2—Ba3—O1^xi^	144.761 (3)	O1^i^—O3—Mn1	42.177 (3)
O2—Ba3—O1^v^	143.162 (3)	O1^i^—O3—O3^xxv^	117.563 (3)
O2—Ba3—O1^iii^	104.145 (8)	O1—O3—O1^i^	55.157 (6)
O2—Ba3—O1^ix^	104.145 (8)	O1—O3—O1^xlvii^	1.6840 (1)
O2—Nb4—O2^xliii^	90.245 (5)	O1—O3—O1^viii^	54.563 (6)
O2^xliv^—Nb4—O2	90.235 (5)	O1—O3—Ba2^l^	144.167 (3)
O2^xliv^—Nb4—O2^xliii^	0.0152 (1)	O1—O3—O2^xv^	113.513 (7)
O2^xxv^—Nb4—O2^xliv^	90.224 (5)	O1—O3—O2^xiv^	113.522 (7)
O2^xxv^—Nb4—O2	0.0152 (1)	O1—O3—O2^xix^	149.102 (3)
O2^xxv^—Nb4—O2^xliii^	90.235 (5)	O1—O3—O2^xxiv^	149.111 (3)
O2^xlv^—Nb4—O2^xxv^	179.9848 (1)	O1—O3—O3^xix^	62.017 (3)
O2^xlv^—Nb4—O2^xliv^	89.765 (5)	O1—O3—O3^xxiv^	62.007 (3)
O2^xlv^—Nb4—O2	180.0000 (1)	O1—O3—O3^xv^	89.6378 (1)
O2^xlv^—Nb4—O2^xliii^	89.755 (5)	O1—O3—O3^xiv^	89.6559 (1)
O2^xxii^—Nb4—O2^xlv^	90.245 (5)	O1—O3—Mn2	109.827 (6)
O2^xxii^—Nb4—O2^xxv^	89.765 (5)	O1—O3—Nb7	109.827 (6)
O2^xxii^—Nb4—O2^xliv^	179.9848 (1)	O1—O3—Mn1	42.159 (3)
O2^xxii^—Nb4—O2	89.755 (5)	O1—O3—O3^xxv^	62.406 (3)
O2^xxii^—Nb4—O2^xliii^	180.0000 (1)	Ba3^liv^—O3—O1	114.709 (4)
O2^xxvii^—Nb4—O2^xxii^	0.0152 (1)	Ba3^liv^—O3—O1^i^	59.758 (2)
O2^xxvii^—Nb4—O2^xlv^	90.235 (5)	Ba3^liv^—O3—O1^xlvii^	113.951 (4)
O2^xxvii^—Nb4—O2^xxv^	89.776 (5)	Ba3^liv^—O3—O1^viii^	60.226 (2)
O2^xxvii^—Nb4—O2^xliv^	180.000	Ba3^liv^—O3—Ba2^l^	90.6515 (8)
O2^xxvii^—Nb4—O2	89.765 (5)	Ba3^liv^—O3—O2^xv^	124.700 (3)
O2^xxvii^—Nb4—O2^xliii^	179.9848 (1)	Ba3^liv^—O3—O2^xiv^	124.696 (3)
O2^xlvi^—Nb4—O2^xxvii^	90.224 (5)	Ba3^liv^—O3—O2^xix^	65.361 (4)
O2^xlvi^—Nb4—O2^xxii^	90.235 (5)	Ba3^liv^—O3—O2^xxiv^	65.365 (4)
O2^xlvi^—Nb4—O2^xlv^	0.0152 (1)	Ba3^liv^—O3—O3^xix^	123.5596 (1)
O2^xlvi^—Nb4—O2^xxv^	180.0000 (1)	Ba3^liv^—O3—O3^xxiv^	123.5930 (1)
O2^xlvi^—Nb4—O2^xliv^	89.776 (5)	Ba3^liv^—O3—O3^xv^	63.7646 (1)
O2^xlvi^—Nb4—O2	179.9848 (1)	Ba3^liv^—O3—O3^xiv^	63.7313 (1)
O2^xlvi^—Nb4—O2^xliii^	89.765 (5)	Ba3^liv^—O3—Mn2	95.4791 (4)
O2^xxix^—Nb4—O2^xlvi^	89.765 (5)	Ba3^liv^—O3—Nb7	95.4791 (4)
O2^xxix^—Nb4—O2^xxvii^	90.245 (5)	Ba3^liv^—O3—Mn1	90.2983 (7)
O2^xxix^—Nb4—O2^xxii^	90.235 (5)	Ba3^liv^—O3—O3^xxv^	174.4939 (4)
O2^xxix^—Nb4—O2^xlv^	89.776 (5)	Ba3^l^—O3—Ba3^liv^	168.9911 (9)
O2^xxix^—Nb4—O2^xxv^	90.235 (5)	Ba3^l^—O3—O1	59.728 (2)
O2^xxix^—Nb4—O2^xliv^	89.755 (5)	Ba3^l^—O3—O1^i^	114.680 (4)
O2^xxix^—Nb4—O2	90.224 (5)	Ba3^l^—O3—O1^xlvii^	60.197 (2)
O2^xxix^—Nb4—O2^xliii^	89.765 (5)	Ba3^l^—O3—O1^viii^	113.922 (4)
O2^xxx^—Nb4—O2^xxix^	0.0152 (1)	Ba3^l^—O3—Ba2^l^	90.6155 (8)
O2^xxx^—Nb4—O2^xlvi^	89.755 (5)	Ba3^l^—O3—O2^xv^	65.327 (4)
O2^xxx^—Nb4—O2^xxvii^	90.235 (5)	Ba3^l^—O3—O2^xiv^	65.330 (4)
O2^xxx^—Nb4—O2^xxii^	90.224 (5)	Ba3^l^—O3—O2^xix^	124.665 (3)
O2^xxx^—Nb4—O2^xlv^	89.765 (5)	Ba3^l^—O3—O2^xxiv^	124.661 (3)
O2^xxx^—Nb4—O2^xxv^	90.245 (5)	Ba3^l^—O3—O3^xix^	63.7290 (1)
O2^xxx^—Nb4—O2^xliv^	89.765 (5)	Ba3^l^—O3—O3^xxiv^	63.6956 (1)
O2^xxx^—Nb4—O2	90.235 (5)	Ba3^l^—O3—O3^xv^	123.5242 (1)
O2^xxx^—Nb4—O2^xliii^	89.776 (5)	Ba3^l^—O3—O3^xiv^	123.5575 (1)
O2^xlii^—Nb4—O2^xxx^	179.9848 (1)	Ba3^l^—O3—Mn2	95.4249 (4)
O2^xlii^—Nb4—O2^xxix^	180.000	Ba3^l^—O3—Nb7	95.4249 (4)
O2^xlii^—Nb4—O2^xlvi^	90.235 (5)	Ba3^l^—O3—Mn1	90.2462 (7)
O2^xlii^—Nb4—O2^xxvii^	89.755 (5)	Ba3^l^—O3—O3^xxv^	5.5028 (4)
O2^xlii^—Nb4—O2^xxii^	89.765 (5)	Ba1^l^—O3—Ba3^l^	84.4928 (4)
O2^xlii^—Nb4—O2^xlv^	90.224 (5)	Ba1^l^—O3—Ba3^liv^	84.5223 (4)
O2^xlii^—Nb4—O2^xxv^	89.765 (5)	Ba1^l^—O3—O1	60.112 (6)
O2^xlii^—Nb4—O2^xliv^	90.245 (5)	Ba1^l^—O3—O1^i^	60.120 (6)
O2^xlii^—Nb4—O2	89.776 (5)	Ba1^l^—O3—O1^xlvii^	58.432 (6)
O2^xlii^—Nb4—O2^xliii^	90.235 (5)	Ba1^l^—O3—O1^viii^	58.439 (6)
O2^xli^—Nb4—O2^xlii^	0.0152 (1)	Ba1^l^—O3—Ba2^l^	100.401 (8)
O2^xli^—Nb4—O2^xxx^	180.0000 (1)	Ba1^l^—O3—O2^xv^	144.409 (3)
O2^xli^—Nb4—O2^xxix^	179.9848 (1)	Ba1^l^—O3—O2^xiv^	144.406 (3)
O2^xli^—Nb4—O2^xlvi^	90.245 (5)	Ba1^l^—O3—O2^xix^	144.462 (3)
O2^xli^—Nb4—O2^xxvii^	89.765 (5)	Ba1^l^—O3—O2^xxiv^	144.459 (3)
O2^xli^—Nb4—O2^xxii^	89.776 (5)	Ba1^l^—O3—O3^xix^	122.020 (3)
O2^xli^—Nb4—O2^xlv^	90.235 (5)	Ba1^l^—O3—O3^xxiv^	122.008 (3)
O2^xli^—Nb4—O2^xxv^	89.755 (5)	Ba1^l^—O3—O3^xv^	122.052 (3)
O2^xli^—Nb4—O2^xliv^	90.235 (5)	Ba1^l^—O3—O3^xiv^	122.040 (3)
O2^xli^—Nb4—O2	89.765 (5)	Ba1^l^—O3—Mn2	168.3144 (1)
O2^xli^—Nb4—O2^xliii^	90.224 (5)	Ba1^l^—O3—Nb7	168.3144 (1)
O3^xix^—Mn1—O3^xxiv^	0.0523 (1)	Ba1^l^—O3—Mn1	89.058 (8)
O3^xiv^—Mn1—O3^xix^	85.035 (6)	Ba1^l^—O3—O3^xxv^	89.9836 (1)
O3^xiv^—Mn1—O3^xxiv^	85.070 (6)	O3^lv^—O3—Ba1^l^	57.980 (3)
O3^xv^—Mn1—O3^xiv^	0.0523 (1)	O3^lv^—O3—Ba3^l^	116.2710 (1)
O3^xv^—Mn1—O3^xix^	84.999 (6)	O3^lv^—O3—Ba3^liv^	56.4404 (1)
O3^xv^—Mn1—O3^xxiv^	85.035 (6)	O3^lv^—O3—O1	117.983 (3)
O3—Mn1—O3^xv^	85.035 (6)	O3^lv^—O3—O1^i^	90.3617 (1)
O3—Mn1—O3^xiv^	84.999 (6)	O3^lv^—O3—O1^xlvii^	116.299 (3)
O3—Mn1—O3^xix^	85.070 (6)	O3^lv^—O3—O1^viii^	89.4136 (1)
O3—Mn1—O3^xxiv^	85.035 (6)	O3^lv^—O3—Ba2^l^	54.727 (3)
O3^xxv^—Mn1—O3	0.0523 (1)	O3^lv^—O3—O2^xv^	118.070 (3)
O3^xxv^—Mn1—O3^xv^	85.070 (6)	O3^lv^—O3—O2^xiv^	118.060 (3)
O3^xxv^—Mn1—O3^xiv^	85.035 (6)	O3^lv^—O3—O2^xix^	88.5814 (1)
O3^xxv^—Mn1—O3^xix^	85.035 (6)	O3^lv^—O3—O2^xxiv^	88.5752 (1)
O3^xxv^—Mn1—O3^xxiv^	84.999 (6)	O3^lv^—O3—O3^xix^	180.000
O1^viii^—Mn1—O3^xxv^	94.190 (6)	O3^lv^—O3—O3^xxiv^	179.9665 (1)
O1^viii^—Mn1—O3	94.155 (6)	O3^lv^—O3—O3^xv^	120.033
O1^viii^—Mn1—O3^xv^	96.079 (6)	O3^lv^—O3—O3^xiv^	120.000
O1^viii^—Mn1—O3^xiv^	96.043 (6)	O3^lv^—O3—Mn2	131.167 (3)
O1^viii^—Mn1—O3^xix^	178.6191 (1)	O3^lv^—O3—Nb7	131.167 (3)
O1^viii^—Mn1—O3^xxiv^	178.5674 (1)	O3^lv^—O3—Mn1	132.535 (3)
O1^i^—Mn1—O1^viii^	2.7829 (2)	O3^lv^—O3—O3^xxv^	120.000
O1^i^—Mn1—O3^xxv^	96.079 (6)	O3^lvi^—O3—O3^lv^	60.027
O1^i^—Mn1—O3	96.043 (6)	O3^lvi^—O3—Ba1^l^	57.970 (3)
O1^i^—Mn1—O3^xv^	94.190 (6)	O3^lvi^—O3—Ba3^l^	56.4158 (1)
O1^i^—Mn1—O3^xiv^	94.155 (6)	O3^lvi^—O3—Ba3^liv^	116.2955 (1)
O1^i^—Mn1—O3^xix^	178.5674 (1)	O3^lvi^—O3—O1	90.3297 (1)
O1^i^—Mn1—O3^xxiv^	178.6191 (1)	O3^lvi^—O3—O1^i^	117.980 (3)
O1^xlvii^—Mn1—O1^i^	84.750 (6)	O3^lvi^—O3—O1^xlvii^	89.3821 (1)
O1^xlvii^—Mn1—O1^viii^	82.831 (6)	O3^lvi^—O3—O1^viii^	116.295 (3)
O1^xlvii^—Mn1—O3^xxv^	94.155 (6)	O3^lvi^—O3—Ba2^l^	54.716 (3)
O1^xlvii^—Mn1—O3	94.190 (6)	O3^lvi^—O3—O2^xv^	88.5487 (1)
O1^xlvii^—Mn1—O3^xv^	178.6191 (1)	O3^lvi^—O3—O2^xiv^	88.5425 (1)
O1^xlvii^—Mn1—O3^xiv^	178.5674 (1)	O3^lvi^—O3—O2^xix^	118.069 (3)
O1^xlvii^—Mn1—O3^xix^	96.079 (6)	O3^lvi^—O3—O2^xxiv^	118.058 (3)
O1^xlvii^—Mn1—O3^xxiv^	96.043 (6)	O3^lvi^—O3—O3^xix^	119.973
O1—Mn1—O1^xlvii^	2.7829 (2)	O3^lvi^—O3—O3^xxiv^	119.940
O1—Mn1—O1^i^	86.596 (6)	O3^lvi^—O3—O3^xv^	179.940
O1—Mn1—O1^viii^	84.750 (6)	O3^lvi^—O3—O3^xiv^	179.9732 (1)
O1—Mn1—O3^xxv^	96.043 (6)	O3^lvi^—O3—Mn2	131.120 (3)
O1—Mn1—O3	96.079 (6)	O3^lvi^—O3—Nb7	131.120 (3)
O1—Mn1—O3^xv^	178.5674 (1)	O3^lvi^—O3—Mn1	132.485 (3)
O1—Mn1—O3^xiv^	178.6191 (1)	O3^lvi^—O3—O3^xxv^	59.973
O1—Mn1—O3^xix^	94.190 (6)	O3^lvii^—O3—O3^lvi^	60.000
O1—Mn1—O3^xxiv^	94.155 (6)	O3^lvii^—O3—O3^lv^	0.0268 (1)
O1^iii^—Mn1—O1	84.750 (6)	O3^lvii^—O3—Ba1^l^	57.970 (3)
O1^iii^—Mn1—O1^xlvii^	86.596 (6)	O3^lvii^—O3—Ba3^l^	116.2443 (1)
O1^iii^—Mn1—O1^i^	82.831 (6)	O3^lvii^—O3—Ba3^liv^	56.4671 (1)
O1^iii^—Mn1—O1^viii^	84.750 (6)	O3^lvii^—O3—O1	117.975 (3)
O1^iii^—Mn1—O3^xxv^	178.6191 (1)	O3^lvii^—O3—O1^i^	90.3762 (1)
O1^iii^—Mn1—O3	178.5674 (1)	O3^lvii^—O3—O1^xlvii^	116.291 (3)
O1^iii^—Mn1—O3^xv^	94.155 (6)	O3^lvii^—O3—O1^viii^	89.4275 (1)
O1^iii^—Mn1—O3^xiv^	94.190 (6)	O3^lvii^—O3—Ba2^l^	54.716 (3)
O1^iii^—Mn1—O3^xix^	96.043 (6)	O3^lvii^—O3—O2^xv^	118.061 (3)
O1^iii^—Mn1—O3^xxiv^	96.079 (6)	O3^lvii^—O3—O2^xiv^	118.051 (3)
O1^ix^—Mn1—O1^iii^	2.7829 (2)	O3^lvii^—O3—O2^xix^	88.5963 (1)
O1^ix^—Mn1—O1	82.831 (6)	O3^lvii^—O3—O2^xxiv^	88.5901 (1)
O1^ix^—Mn1—O1^xlvii^	84.750 (6)	O3^lvii^—O3—O3^xix^	179.973
O1^ix^—Mn1—O1^i^	84.750 (6)	O3^lvii^—O3—O3^xxiv^	179.940
O1^ix^—Mn1—O1^viii^	86.596 (6)	O3^lvii^—O3—O3^xv^	120.060
O1^ix^—Mn1—O3^xxv^	178.5674 (1)	O3^lvii^—O3—O3^xiv^	120.027
O1^ix^—Mn1—O3	178.6191 (1)	O3^lvii^—O3—Mn2	131.181 (3)
O1^ix^—Mn1—O3^xv^	96.043 (6)	O3^lvii^—O3—Nb7	131.181 (3)
O1^ix^—Mn1—O3^xiv^	96.079 (6)	O3^lvii^—O3—Mn1	132.549 (3)
O1^ix^—Mn1—O3^xix^	94.155 (6)	O3^lvii^—O3—O3^xxv^	119.973
O1^ix^—Mn1—O3^xxiv^	94.190 (6)	O3^lviii^—O3—O3^lvii^	59.973
Mn1^viii^—Mn1—O1^ix^	51.100 (4)	O3^lviii^—O3—O3^lvi^	0.0268 (1)
Mn1^viii^—Mn1—O1^iii^	51.100 (4)	O3^lviii^—O3—O3^lv^	60.000
Mn1^viii^—Mn1—O1	51.100 (4)	O3^lviii^—O3—Ba1^l^	57.960 (3)
Mn1^viii^—Mn1—O1^xlvii^	51.100 (4)	O3^lviii^—O3—Ba3^l^	56.4425 (1)
Mn1^viii^—Mn1—O1^i^	51.100 (4)	O3^lviii^—O3—Ba3^liv^	116.2687 (1)
Mn1^viii^—Mn1—O1^viii^	51.100 (4)	O3^lviii^—O3—O1	90.3441 (1)
Mn1^viii^—Mn1—O3^xxv^	128.706 (4)	O3^lviii^—O3—O1^i^	117.972 (3)
Mn1^viii^—Mn1—O3	128.706 (4)	O3^lviii^—O3—O1^xlvii^	89.3960 (1)
Mn1^viii^—Mn1—O3^xv^	128.706 (4)	O3^lviii^—O3—O1^viii^	116.287 (3)
Mn1^viii^—Mn1—O3^xiv^	128.706 (4)	O3^lviii^—O3—Ba2^l^	54.705 (3)
Mn1^viii^—Mn1—O3^xix^	128.706 (4)	O3^lviii^—O3—O2^xv^	88.5636 (1)
Mn1^viii^—Mn1—O3^xxiv^	128.706 (4)	O3^lviii^—O3—O2^xiv^	88.5574 (1)
Nb7—Mn1—Mn1^viii^	180.000	O3^lviii^—O3—O2^xix^	118.059 (3)
Nb7—Mn1—O1^ix^	128.900 (4)	O3^lviii^—O3—O2^xxiv^	118.049 (3)
Nb7—Mn1—O1^iii^	128.900 (4)	O3^lviii^—O3—O3^xix^	120.000
Nb7—Mn1—O1	128.900 (4)	O3^lviii^—O3—O3^xxiv^	119.967
Nb7—Mn1—O1^xlvii^	128.900 (4)	O3^lviii^—O3—O3^xv^	179.9665 (1)
Nb7—Mn1—O1^i^	128.900 (4)	O3^lviii^—O3—O3^xiv^	180.000
Nb7—Mn1—O1^viii^	128.900 (4)	O3^lviii^—O3—Mn2	131.133 (3)
Nb7—Mn1—O3^xxv^	51.294 (4)	O3^lviii^—O3—Nb7	131.133 (3)
Nb7—Mn1—O3	51.294 (4)	O3^lviii^—O3—Mn1	132.500 (3)
Nb7—Mn1—O3^xv^	51.294 (4)	O3^lviii^—O3—O3^xxv^	60.000

Symmetry codes: (i) −*x*+*y*, *y*, −*z*+1/2; (ii) −*x*+*y*, −*x*, −*z*+1/2; (iii) −*y*+1, *x*−*y*+1, −*z*+1/2; (iv) *x*+1, *x*−*y*+1, −*z*+1/2; (v) −*y*+1, −*x*, −*z*+1/2; (vi) *x*+1, *y*, *z*; (vii) −*x*+*y*, *y*−1, −*z*+1/2; (viii) −*x*+*y*, −*x*+1, −*z*+1/2; (ix) *x*, *x*−*y*+1, −*z*+1/2; (x) −*y*+2, *x*−*y*+1, −*z*+1/2; (xi) *x*, *y*−1, *z*; (xii) −*y*+2, −*x*+1, −*z*+1/2; (xiii) −*x*+*y*, *y*−1, *z*; (xiv) *x*, *x*−*y*+1, *z*; (xv) −*x*+*y*, −*x*+1, *z*; (xvi) *x*, *y*−1, −*z*+1/2; (xvii) −*y*+2, *x*−*y*+1, *z*; (xviii) −*y*+2, −*x*+1, *z*; (xix) −*y*+1, −*x*+1, *z*; (xx) −*y*+1, *x*−*y*, *z*; (xxi) −*x*+*y*+1, *y*, *z*; (xxii) *x*, *x*−*y*, *z*; (xxiii) −*x*+*y*+1, −*x*+1, *z*; (xxiv) −*y*+1, *x*−*y*+1, *z*; (xxv) −*x*+*y*, *y*, *z*; (xxvi) −*y*+1, −*x*, *z*; (xxvii) −*x*+*y*, −*x*, *z*; (xxviii) *x*+1, *x*−*y*+1, *z*; (xxix) *y*, *x*, −*z*+1; (xxx) *y*, −*x*+*y*, −*z*+1; (xxxi) *x*−*y*+1, *x*, −*z*+1; (xxxii) −*x*+1, −*x*+*y*, −*z*+1; (xxxiii) −*x*+1, −*y*+1, −*z*+1; (xxxiv) *x*−*y*+1, −*y*+1, −*z*+1; (xxxv) −*x*+*y*−1, −*x*, −*z*+1/2; (xxxvi) −*x*+*y*−1, *y*−1, −*z*+1/2; (xxxvii) *x*−1, *y*−1, *z*; (xxxviii) *x*−1, *x*−*y*, *z*; (xxxix) −*x*+*y*−1, *y*−1, *z*; (xl) −*x*+*y*−1, −*x*, *z*; (xli) −*y*, *x*−*y*, *z*; (xlii) −*y*, −*x*, *z*; (xliii) −*x*, −*x*+*y*, −*z*+1; (xliv) *x*−*y*, *x*, −*z*+1; (xlv) −*x*, −*y*, −*z*+1; (xlvi) *x*−*y*, −*y*, −*z*+1; (xlvii) −*y*+1, −*x*+1, −*z*+1/2; (xlviii) *x*, *y*, −*z*+1/2; (xlix) *x*−1, *y*, *z*; (l) *x*, *y*+1, *z*; (li) −*x*+*y*−1, *y*, −*z*+1/2; (lii) −*x*+*y*−1, −*x*+1, −*z*+1/2; (liii) −*y*+1, *x*−*y*+2, −*z*+1/2; (liv) *x*+1, *y*+1, *z*; (lv) −*y*+2, −*x*+2, *z*; (lvi) −*x*+*y*, −*x*+2, *z*; (lvii) −*y*+2, *x*−*y*+2, *z*; (lviii) *x*, *x*−*y*+2, *z*.

### (barium_niobiumV_manganeseII_oxide_2) Crystal data

**Table:**

(Ba3MnNb2O_9_)_0.333_	*V* = 557.45 (11) Å^3^
*M**_r_* = 265.58	*Z* = 8
Cubic, *F**m*3*m*	*D*_x_ = 6.329 (1) Mg m^−^^3^
*a* = 8.2300 (5) Å	*T* = 300 K

### (barium_niobiumV_manganeseII_oxide_2) Data collection

**Table:**

SSRL beam line 2-1 diffractometer	

### (barium_niobiumV_manganeseII_oxide_2) Fractional atomic coordinates and isotropic or equivalent isotropic displacement parameters (Å^2^)

**Table:**

	*x*	*y*	*z*	*B*_iso_*/*B*_eq_	Occ. (<1)
Ba1	0.25	0.25	0.25	0.48341	
Nb1	0	0	0	0.48341	0.3333
Mn1	0	0	0	0.48341	0.6667
Nb2	0.5	0.5	0.5	0.48341	
O1	0.247	0	0	0.48341	

### (barium_niobiumV_manganeseII_oxide_2) Geometric parameters (Å, º)

**Table:**

Ba1—O1^i^	2.9099 (2)	Mn1—O1^xiii^	2.0328 (1)
Ba1—O1^ii^	2.9099 (2)	Mn1—O1^xiv^	2.0328 (1)
Ba1—O1^iii^	2.9099 (2)	Nb2—O1^vii^	2.0822 (1)
Ba1—O1^iv^	2.9099 (2)	Nb2—O1^viii^	2.0822 (1)
Ba1—O1^v^	2.9099 (2)	Nb2—O1^xi^	2.0822 (1)
Ba1—O1^vi^	2.9099 (2)	Nb2—O1^xv^	2.0822 (1)
Ba1—O1	2.9099 (2)	Nb2—O1^xvi^	2.0822 (1)
Ba1—O1^vii^	2.9099 (2)	Nb2—O1^xvii^	2.0822 (1)
Ba1—O1^viii^	2.9099 (2)	O1—Nb1	2.0328 (1)
Ba1—O1^ix^	2.9099 (2)	O1—Mn1	2.0328 (1)
Ba1—O1^x^	2.9099 (2)	O1—Nb2^xviii^	2.0822 (1)
Ba1—O1^xi^	2.9099 (2)	O1—O1^ix^	2.8748 (2)
Nb1—Mn1	0.00000	O1—O1^x^	2.8748 (2)
Nb1—O1^ix^	2.0328 (1)	O1—O1^xii^	2.8748 (2)
Nb1—O1^xii^	2.0328 (1)	O1—O1^xiii^	2.8748 (2)
Nb1—O1	2.0328 (1)	O1—Ba1^xix^	2.9099 (2)
Nb1—O1^x^	2.0328 (1)	O1—Ba1^xx^	2.9099 (2)
Nb1—O1^xiii^	2.0328 (1)	O1—Ba1	2.9099 (2)
Nb1—O1^xiv^	2.0328 (1)	O1—Ba1^xxi^	2.9099 (2)
Mn1—Nb1	0.00000	O1—O1^xxii^	2.9447 (2)
Mn1—O1^ix^	2.0328 (1)	O1—O1^xxiii^	2.9447 (2)
Mn1—O1^xii^	2.0328 (1)	O1—O1^i^	2.9447 (2)
Mn1—O1	2.0328 (1)	O1—O1^iv^	2.9447 (2)
Mn1—O1^x^	2.0328 (1)		
			
O1^ii^—Ba1—O1^i^	59.205	O1^xvi^—Nb2—O1^xv^	90.000
O1^iii^—Ba1—O1^ii^	179.028	O1^xvi^—Nb2—O1^xi^	90.000
O1^iii^—Ba1—O1^i^	119.998	O1^xvi^—Nb2—O1^viii^	180.000
O1^iv^—Ba1—O1^iii^	59.205	O1^xvi^—Nb2—O1^vii^	90.000
O1^iv^—Ba1—O1^ii^	119.998	O1^xvii^—Nb2—O1^xvi^	90.000
O1^iv^—Ba1—O1^i^	60.793	O1^xvii^—Nb2—O1^xv^	90.000
O1^v^—Ba1—O1^iv^	179.028	O1^xvii^—Nb2—O1^xi^	180.000
O1^v^—Ba1—O1^iii^	119.998	O1^xvii^—Nb2—O1^viii^	90.000
O1^v^—Ba1—O1^ii^	60.793	O1^xvii^—Nb2—O1^vii^	90.000
O1^v^—Ba1—O1^i^	119.998	Mn1—O1—Nb1	0.000
O1^vi^—Ba1—O1^v^	59.205	Nb2^xviii^—O1—Mn1	180.000
O1^vi^—Ba1—O1^iv^	119.998	Nb2^xviii^—O1—Nb1	180.000
O1^vi^—Ba1—O1^iii^	60.793	O1^ix^—O1—Nb2^xviii^	135.000
O1^vi^—Ba1—O1^ii^	119.998	O1^ix^—O1—Mn1	45.000
O1^vi^—Ba1—O1^i^	179.028	O1^ix^—O1—Nb1	45.000
O1—Ba1—O1^vi^	119.998	O1^x^—O1—O1^ix^	60.000
O1—Ba1—O1^v^	119.998	O1^x^—O1—Nb2^xviii^	135.000
O1—Ba1—O1^iv^	60.793	O1^x^—O1—Mn1	45.000
O1—Ba1—O1^iii^	90.004	O1^x^—O1—Nb1	45.000
O1—Ba1—O1^ii^	90.004	O1^xii^—O1—O1^x^	90.000
O1—Ba1—O1^i^	60.793	O1^xii^—O1—O1^ix^	60.000
O1^vii^—Ba1—O1	119.998	O1^xii^—O1—Nb2^xviii^	135.000
O1^vii^—Ba1—O1^vi^	119.998	O1^xii^—O1—Mn1	45.000
O1^vii^—Ba1—O1^v^	90.004	O1^xii^—O1—Nb1	45.000
O1^vii^—Ba1—O1^iv^	90.004	O1^xiii^—O1—O1^xii^	60.000
O1^vii^—Ba1—O1^iii^	119.998	O1^xiii^—O1—O1^x^	60.000
O1^vii^—Ba1—O1^ii^	59.205	O1^xiii^—O1—O1^ix^	90.000
O1^vii^—Ba1—O1^i^	59.205	O1^xiii^—O1—Nb2^xviii^	135.000
O1^viii^—Ba1—O1^vii^	60.793	O1^xiii^—O1—Mn1	45.000
O1^viii^—Ba1—O1	179.028	O1^xiii^—O1—Nb1	45.000
O1^viii^—Ba1—O1^vi^	59.205	Ba1^xix^—O1—O1^xiii^	120.397
O1^viii^—Ba1—O1^v^	59.205	Ba1^xix^—O1—O1^xii^	60.397
O1^viii^—Ba1—O1^iv^	119.998	Ba1^xix^—O1—O1^x^	120.397
O1^viii^—Ba1—O1^iii^	90.004	Ba1^xix^—O1—O1^ix^	60.397
O1^viii^—Ba1—O1^ii^	90.004	Ba1^xix^—O1—Nb2^xviii^	89.514
O1^viii^—Ba1—O1^i^	119.998	Ba1^xix^—O1—Mn1	90.486
O1^ix^—Ba1—O1^viii^	119.998	Ba1^xix^—O1—Nb1	90.486
O1^ix^—Ba1—O1^vii^	179.028	Ba1^xx^—O1—Ba1^xix^	179.028
O1^ix^—Ba1—O1	59.205	Ba1^xx^—O1—O1^xiii^	60.397
O1^ix^—Ba1—O1^vi^	60.793	Ba1^xx^—O1—O1^xii^	120.397
O1^ix^—Ba1—O1^v^	90.004	Ba1^xx^—O1—O1^x^	60.397
O1^ix^—Ba1—O1^iv^	90.004	Ba1^xx^—O1—O1^ix^	120.397
O1^ix^—Ba1—O1^iii^	60.793	Ba1^xx^—O1—Nb2^xviii^	89.514
O1^ix^—Ba1—O1^ii^	119.998	Ba1^xx^—O1—Mn1	90.486
O1^ix^—Ba1—O1^i^	119.998	Ba1^xx^—O1—Nb1	90.486
O1^x^—Ba1—O1^ix^	59.205	Ba1—O1—Ba1^xx^	89.996
O1^x^—Ba1—O1^viii^	119.998	Ba1—O1—Ba1^xix^	89.996
O1^x^—Ba1—O1^vii^	119.998	Ba1—O1—O1^xiii^	120.397
O1^x^—Ba1—O1	59.205	Ba1—O1—O1^xii^	120.397
O1^x^—Ba1—O1^vi^	90.004	Ba1—O1—O1^x^	60.397
O1^x^—Ba1—O1^v^	60.793	Ba1—O1—O1^ix^	60.397
O1^x^—Ba1—O1^iv^	119.998	Ba1—O1—Nb2^xviii^	89.514
O1^x^—Ba1—O1^iii^	119.998	Ba1—O1—Mn1	90.486
O1^x^—Ba1—O1^ii^	60.793	Ba1—O1—Nb1	90.486
O1^x^—Ba1—O1^i^	90.004	Ba1^xxi^—O1—Ba1	179.028
O1^xi^—Ba1—O1^x^	179.028	Ba1^xxi^—O1—Ba1^xx^	89.996
O1^xi^—Ba1—O1^ix^	119.998	Ba1^xxi^—O1—Ba1^xix^	89.996
O1^xi^—Ba1—O1^viii^	60.793	Ba1^xxi^—O1—O1^xiii^	60.397
O1^xi^—Ba1—O1^vii^	60.793	Ba1^xxi^—O1—O1^xii^	60.397
O1^xi^—Ba1—O1	119.998	Ba1^xxi^—O1—O1^x^	120.397
O1^xi^—Ba1—O1^vi^	90.004	Ba1^xxi^—O1—O1^ix^	120.397
O1^xi^—Ba1—O1^v^	119.998	Ba1^xxi^—O1—Nb2^xviii^	89.514
O1^xi^—Ba1—O1^iv^	59.205	Ba1^xxi^—O1—Mn1	90.486
O1^xi^—Ba1—O1^iii^	59.205	Ba1^xxi^—O1—Nb1	90.486
O1^xi^—Ba1—O1^ii^	119.998	O1^xxii^—O1—Ba1^xxi^	59.603
O1^xi^—Ba1—O1^i^	90.004	O1^xxii^—O1—Ba1	119.603
O1^ix^—Nb1—Mn1		O1^xxii^—O1—Ba1^xx^	119.603
O1^xii^—Nb1—O1^ix^	90.000	O1^xxii^—O1—Ba1^xix^	59.603
O1^xii^—Nb1—Mn1		O1^xxii^—O1—O1^xiii^	120.000
O1—Nb1—O1^xii^	90.000	O1^xxii^—O1—O1^xii^	90.000
O1—Nb1—O1^ix^	90.000	O1^xxii^—O1—O1^x^	180.000
O1—Nb1—Mn1		O1^xxii^—O1—O1^ix^	120.000
O1^x^—Nb1—O1	90.000	O1^xxii^—O1—Nb2^xviii^	45.000
O1^x^—Nb1—O1^xii^	180.000	O1^xxii^—O1—Mn1	135.000
O1^x^—Nb1—O1^ix^	90.000	O1^xxii^—O1—Nb1	135.000
O1^x^—Nb1—Mn1		O1^xxiii^—O1—O1^xxii^	60.000
O1^xiii^—Nb1—O1^x^	90.000	O1^xxiii^—O1—Ba1^xxi^	59.603
O1^xiii^—Nb1—O1	90.000	O1^xxiii^—O1—Ba1	119.603
O1^xiii^—Nb1—O1^xii^	90.000	O1^xxiii^—O1—Ba1^xx^	59.603
O1^xiii^—Nb1—O1^ix^	180.000	O1^xxiii^—O1—Ba1^xix^	119.603
O1^xiii^—Nb1—Mn1		O1^xxiii^—O1—O1^xiii^	90.000
O1^xiv^—Nb1—O1^xiii^	90.000	O1^xxiii^—O1—O1^xii^	120.000
O1^xiv^—Nb1—O1^x^	90.000	O1^xxiii^—O1—O1^x^	120.000
O1^xiv^—Nb1—O1	180.000	O1^xxiii^—O1—O1^ix^	180.0000 (1)
O1^xiv^—Nb1—O1^xii^	90.000	O1^xxiii^—O1—Nb2^xviii^	45.000
O1^xiv^—Nb1—O1^ix^	90.000	O1^xxiii^—O1—Mn1	135.000
O1^xiv^—Nb1—Mn1		O1^xxiii^—O1—Nb1	135.000
O1^ix^—Mn1—Nb1		O1^i^—O1—O1^xxiii^	60.000
O1^xii^—Mn1—O1^ix^	90.000	O1^i^—O1—O1^xxii^	90.000
O1^xii^—Mn1—Nb1		O1^i^—O1—Ba1^xxi^	119.603
O1—Mn1—O1^xii^	90.000	O1^i^—O1—Ba1	59.603
O1—Mn1—O1^ix^	90.000	O1^i^—O1—Ba1^xx^	59.603
O1—Mn1—Nb1		O1^i^—O1—Ba1^xix^	119.603
O1^x^—Mn1—O1	90.000	O1^i^—O1—O1^xiii^	120.000
O1^x^—Mn1—O1^xii^	180.000	O1^i^—O1—O1^xii^	180.0000 (1)
O1^x^—Mn1—O1^ix^	90.000	O1^i^—O1—O1^x^	90.000
O1^x^—Mn1—Nb1		O1^i^—O1—O1^ix^	120.000
O1^xiii^—Mn1—O1^x^	90.000	O1^i^—O1—Nb2^xviii^	45.000
O1^xiii^—Mn1—O1	90.000	O1^i^—O1—Mn1	135.000
O1^xiii^—Mn1—O1^xii^	90.000	O1^i^—O1—Nb1	135.000
O1^xiii^—Mn1—O1^ix^	180.000	O1^iv^—O1—O1^i^	60.000
O1^xiii^—Mn1—Nb1		O1^iv^—O1—O1^xxiii^	90.000
O1^xiv^—Mn1—O1^xiii^	90.000	O1^iv^—O1—O1^xxii^	60.000
O1^xiv^—Mn1—O1^x^	90.000	O1^iv^—O1—Ba1^xxi^	119.603
O1^xiv^—Mn1—O1	180.000	O1^iv^—O1—Ba1	59.603
O1^xiv^—Mn1—O1^xii^	90.000	O1^iv^—O1—Ba1^xx^	119.603
O1^xiv^—Mn1—O1^ix^	90.000	O1^iv^—O1—Ba1^xix^	59.603
O1^xiv^—Mn1—Nb1		O1^iv^—O1—O1^xiii^	180.000
O1^viii^—Nb2—O1^vii^	90.000	O1^iv^—O1—O1^xii^	120.000
O1^xi^—Nb2—O1^viii^	90.000	O1^iv^—O1—O1^x^	120.000
O1^xi^—Nb2—O1^vii^	90.000	O1^iv^—O1—O1^ix^	90.000
O1^xv^—Nb2—O1^xi^	90.000	O1^iv^—O1—Nb2^xviii^	45.000
O1^xv^—Nb2—O1^viii^	90.000	O1^iv^—O1—Mn1	135.000
O1^xv^—Nb2—O1^vii^	180.000	O1^iv^—O1—Nb1	135.000

Symmetry codes: (i) −*y*+1/2, −*z*, −*x*+1/2; (ii) −*x*+1/2, −*y*, *z*+1/2; (iii) −*x*+1/2, −*y*+1/2, *z*; (iv) −*y*+1/2, −*x*+1/2, −*z*; (v) −*y*, −*x*+1/2, −*z*+1/2; (vi) −*y*, −*z*+1/2, −*x*+1/2; (vii) −*y*+1/2, *x*, −*z*+1/2; (viii) *x*, *y*+1/2, *z*+1/2; (ix) −*y*, *x*, −*z*; (x) −*y*, −*z*, *x*; (xi) −*y*+1/2, −*z*+1/2, *x*; (xii) −*y*, −*z*, −*x*; (xiii) −*y*, −*x*, −*z*; (xiv) −*x*, −*y*, *z*; (xv) −*y*+1/2, −*x*+1, −*z*+1/2; (xvi) −*x*+1, −*y*+1/2, *z*+1/2; (xvii) −*y*+1/2, −*z*+1/2, −*x*+1; (xviii) *x*, *y*−1/2, *z*−1/2; (xix) *z*, *y*, −*x*; (xx) *z*, −*x*, *y*; (xxi) *z*, −*x*, −*y*; (xxii) −*y*+1/2, −*z*, *x*−1/2; (xxiii) −*y*+1/2, *x*−1/2, −*z*.
